# Establishing the South Australian Macrobenthic Traits (SAMT) database: A trait classification for functional assessments

**DOI:** 10.1002/ece3.7040

**Published:** 2020-11-27

**Authors:** Orlando Lam‐Gordillo, Ryan Baring, Sabine Dittmann

**Affiliations:** ^1^ College of Science and Engineering Flinders University Adelaide SA Australia

**Keywords:** Australia, benthos, Biological traits, ecosystem functioning, functional group, macrofauna

## Abstract

Trait‐based approaches are increasingly used as a proxy for understanding the relationship between biodiversity and ecosystem functioning. Macrobenthic fauna are considered one of the major providers of ecosystem functions in marine soft sediments; however, several gaps persist in the knowledge of their trait classification, limiting the potential use of functional assessments. While trait databases are available for the well‐studied North Atlantic benthic fauna, no such trait classification system exists for Australia. Here, we present the South Australian Macrobenthic Traits (SAMT) database, the first comprehensive assessment of macrobenthic fauna traits in temperate Australian waters. The SAMT database includes 13 traits and 54 trait‐modalities (e.g., life history, morphology, physiology, and behavior), and is based on records of macrobenthic fauna from South Australia. We provide trait information for more than 250 macrobenthic taxa, including outcomes from a fuzzy coding procedure, as well as an R package for using and analyzing the SAMT database. The establishment of the SAMT constitutes the foundation for a comprehensive macrobenthic trait database for the wider southern Australian region that could facilitate future research on functional perspectives, such as assessments of functional diversity and changes to ecosystem functioning.

## Graphical abstract

Step‐by‐step processes for assessing ecosystem functioning. The use of SAMT database is highlighted as an essential step for understanding ecosystem functioning.

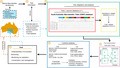

## 1 INTRODUCTION

Trait‐based approaches have become topical in ecological research for understanding the relationship between species (biodiversity) and ecosystem functioning, ecosystem processes, ecosystem services, or responses to anthropogenic disturbances (Bolam et al., 2016; Bremner et al., 2003, 2006; Cano‐Barbcil et al., 2019; Weiss & Ray, 2019). Trait‐based approaches are also used to measure several functional indices (e.g., functional diversity: functional divergence, functional redundancy, and functional richness) and can be used to perform analyses across species pools from distinct geographical areas (Degen & Faulwetter, 2019; Lam‐Gordillo et al., 2020; Mason et al., 2005; Mouchet et al., 2010). Functional approaches are based on different subsets of traits (i.e., species characteristics) as a proxy of ecosystem functioning (Bremner, 2008; Bremner et al., 2006).

Traits can be defined as properties of organisms that can be measured, usually at the organism level and used comparatively across species. Examples of traits are the life history, morphology, physiology, and behavior characteristics that species can exhibit (Bremner et al., 2006; Degen et al., 2018; Lam‐Gordillo et al., 2020; Petchey & Gaston, 2006; Reiss et al., 2009). Selection of traits is flexible and should include an appropriate range of traits relevant to the specific research question, that is, capture the characteristics of organism for the ecosystem processes under investigation (Beauchard et al., 2017; Costello et al., 2015; Lam‐Gordillo et al., 2020; Petchey & Gaston, 2006).

The use of traits has gained momentum in marine ecology with an growth in published research in recent years, which has improved the understanding of the functioning of marine ecosystems (Cano‐Barbcil et al., 2019; Castro et al., 2019; Costello et al., 2015; Lam‐Gordillo et al., 2020). The increased interest in traits has been particularly evident in the assessment of macrobenthic communities (Beauchard et al., 2017; Degen et al., 2018; Dissanayake et al., 2018; Lam‐Gordillo et al., 2020). Macrobenthic invertebrates have long been recognized as important providers of ecological processes and ecosystem functions in soft sediments due to their capability to enhance recycling of nutrients, modifying sediment properties (e.g., bioturbation, exchange processes). They are also useful bioindicators of pollution and other environmental stressors (Dissanayake et al., 2019; Liu et al., 2019; Reiss et al., 2009; Dittmann et al. 2015; Shojaei et al., 2015).

Throughout the literature, several traits have been proposed to assess the relationship between macrobenthic fauna and ecosystem functioning; however, there are no standardized definitions for traits. In addition, the deficiency on species trait information, data accessibility, and different levels of taxonomic resolution make the selection and use of traits even harder (Lam‐Gordillo et al., 2020). In order to address these issues, some frameworks for assessing biological traits in marine fauna have been suggested, as well as standardized guidelines for the analysis and interpretation of this information (Beauchard et al., 2017; Degen et al., 2018; Lam‐Gordillo et al., 2020).

The southern Australian coast is the longest east–west temperate coastline in the southern hemisphere with a diversity of sedimentary habitats (Short, 2020). However, information about traits of macrobenthic fauna from this region is scarce or nonexistent (Lam‐Gordillo et al., 2020). The limited information about traits, combined with gaps in the taxonomic knowledge of southern Australian benthic species, has limited the use of functional assessments for management and conservation purposes, as well as understanding benthic ecosystem functioning in this part of the world.

Here, we present the South Australian Macrobenthic Traits database (SAMT), to advance trait‐based approaches for southern temperate coastlines. The trait information provided is based on previous studies for comparability and presented in an easily accessible database for downloading and sharing among researchers (Beauchard et al., 2017; Costello et al., 2015; Degen et al., 2018; Lam‐Gordillo et al., 2020). In addition, we present a flow chart detailing the step‐by‐step process of assessing ecosystem functioning and highlighting the utility of the SAMT database for accomplishing this task. This is the first comprehensive assessment of traits of the South Australian macrobenthic fauna, with the aim to facilitate further research across southern Australian temperate marine waters on functional perspectives, elucidating patterns on functional diversity and detect changes in ecosystem functioning.

## 2 METHODS

### 2.1 Data acquired

**FIGURE 1:**
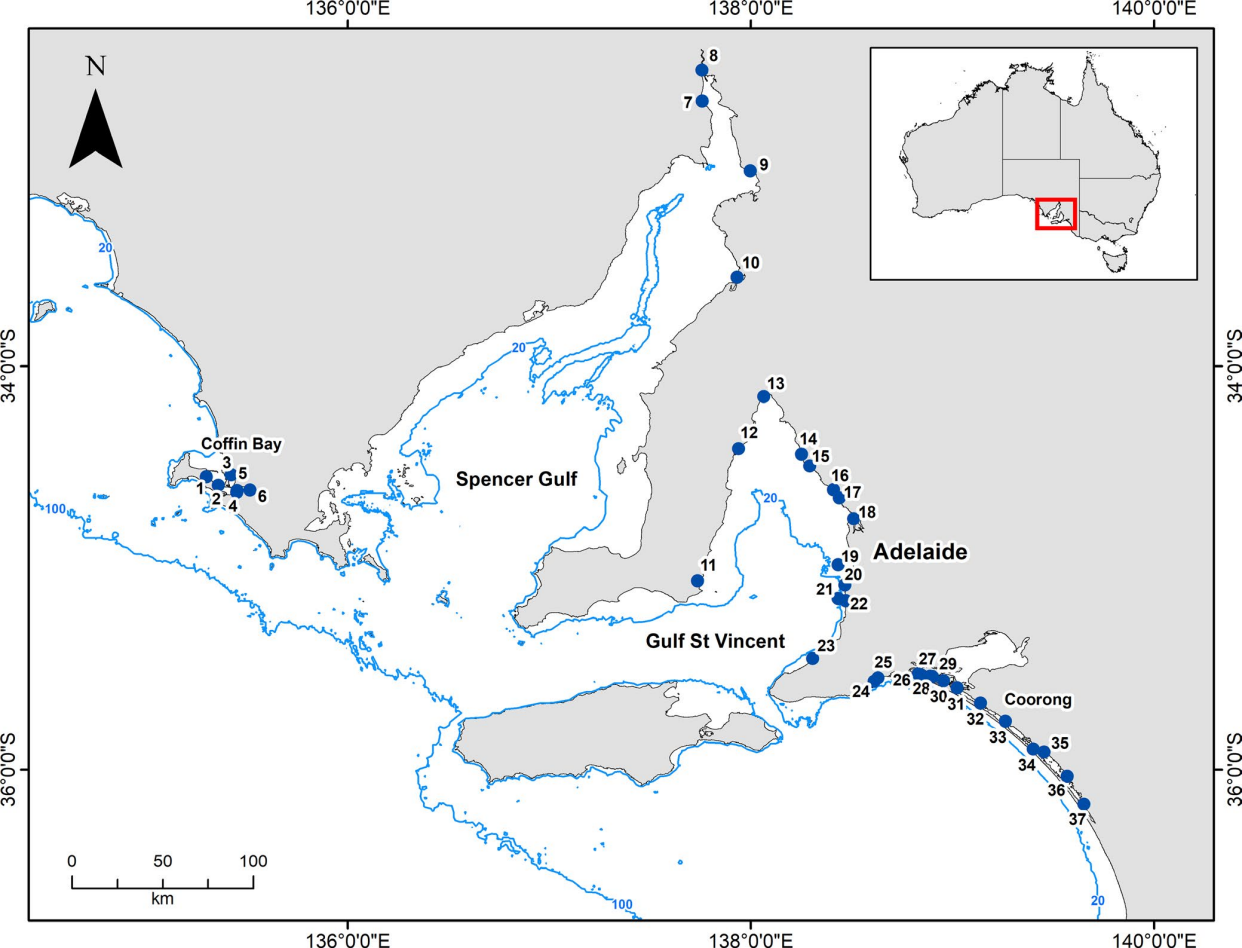
Localities of South Australia from where information about taxa traits were used in this study. (1) Port Douglas; (2) Eely Point; (3) Mount Dutton Bay; (4) Long Beach; (5) Crinoline Point; (6) Kellidie Bay; (7) Blanche Harbor; (8) Curlew Point; (9) Port Germain; (10) Fisherman Bay; (11) Coobowie; (12) Tiddy Widdy; (13) Port Arthur; (14) Port Parham; (15) Thompson's Beach; (16) Middle Beach; (17) Port Gawler; (18) Section Bank; (19) Glenelg; (20) Port Stanvac; (21) Port Noarlunga; (22) Onkaparinga; (23) Normanville; (24) Hindmarsh River; (25) Inman River; (26) Monument Rd; (27) Tarni Warra; (28) Hunters Creek; (29) Mundoo Channel; (30) Ewe Island; (31) Pelican Point; (32) Mulbin Yerrok; (33) Noonameena; (34) Parnka Point; (35) Villa de Yumpa; (36) Jack Point; (37) Loop Rd

A dataset was compiled from previous projects led by the senior author on macrobenthic fauna in soft sediments of South Australia (Table S1), from 37 different localities within this region (Figure 1). The dataset encompasses records from inter‐ and shallow subtidal soft sediments in coastal embayments, lagoons, and inverse estuaries, representative of coastal sedimentary habitats along the arid and warm temperate coastline of southern Australia.

### 2.2 Selection of traits

Selection of traits was based on the most commonly used traits for assessing macrobenthic fauna (Lam‐Gordillo et al., 2020), ensuring that the selected biological traits could be compared across studies (Degen et al., 2018), geographical areas (Bremner et al., 2006), and are applicable to most benthic taxa (Costello et al., 2015). The selected traits capture the four subject areas “Biology,” “Habitat,” “Life‐history,” and “Larval” introduced by Costello et al. (2015) to structure trait categories. In total, based on Lam‐Gordillo et al. (2020), 13 traits and 54 trait‐modalities were assessed (Table 1).

**TABLE 1:** Details of 13 traits and 54 trait‐modalities included in the South Australian Macrobenthic Traits (SAMT) database

Subject area	Traits	Modalities	Definition	Function and processes	Reference
Biology	Bioturbator	Biodiffusor	Transport processes & modification of sediments by organisms that directly/ indirectly affect sediment composition	Nutrient cycling, sediment reworking, organic matter re‐generation, influence on biogeochemistry	Kristensen et al. (2012); Queiros et al. (2013); Beauchard et al. (2017); Degen and Faulwetter (2019); Liu et al. (2019)
		Bioirrigator			
		No bioturbation			
		Surface modifier			
	Body size	Large (>20 mm)	Maximum body size as adult	Influence on productivity, habitat facilitation, sediment reworking, oxygen consumption	Costello et al. (2015); Beauchard et al. (2017); Degen and Faulwetter (2019); Liu et al. (2019)
		Medium (5–20 mm)			
		Small (0.5–5 mm)			
	Degree of attachment	None	Organism ability to attach to a substratum	Influence on metabolic production, trophic support, habitat facilitation	Bremner (2008); Liu et al. (2019)
		Permanent			
	Feeding mode	Deposit feeder	The mode of food acquisition	Nutrient cycling, resource utilization & facilitation, species demographic control, trophic support	Costello et al. (2015); Beauchard et al. (2017); van der Linden et al. (2017); Degen and Faulwetter (2019); Liu et al. (2019)
		Filter/suspension			
		Grazer/scraper			
		Omnivore			
		Predator			
		Scavenger/opportunist			
		Subsurface deposit feeder			
	Mobility	Mobile	Degree of movement	Nutrient cycling, sediment reworking, trophic support, food source	Costello et al. (2015); Degen and Faulwetter (2019); Liu et al. (2019)
		Sessile/attached			
	Morphology	Hard	External features & structural robustness of an adult organism	Sensitivity, food source, habitat facilitation, survival to disturbances, sediment reworking	Beauchard et al. (2017); Degen and Faulwetter (2019); Liu et al. (2019)
		Hard exoskeleton			
		Hard shell			
		Irregular			
		Round			
		Soft/ Fragile			
		Vermiform			
	Movement method	Burrower	Organism type of movement as an adult	Nutrient cycling, sediment transport, dispersal, recolonization, migration, ability to escape predation	Beauchard et al. (2017); Degen and Faulwetter (2019); Liu et al. (2019)
		Crawler			
		None			
		Swimmer			
	Living habit	Burrower	Organism mode living as an adult	Nutrient cycling, sediment transport, dispersal, habitat creation & facilitation	van der Linden et al. (2017); Degen and Faulwetter (2019); Liu et al. (2019)
		Free living/ Surface crawler			
		Tube dwelling			
Habitat	Sediment position	Attached	Organism relative position on the sediment	Nutrient cycling, sediment transport, habitat creation & facilitation	Costello et al. (2015); Beauchard et al. (2017); van der Linden et al. (2017); Degen and Faulwetter (2019); Liu et al. (2019)
		Bentho‐pelagic			
		Deeper than 3 cm			
		Surface shallow <3 cm			
Larval	Larval type	Pelagic ‐planktotrophic	Larval type & feeding mode	Food source, ability of species dispersal, influence in nutrient cycling	van der Linden et al. (2017); Degen and Faulwetter (2019); Liu et al. (2019)
		Pelagic lecthrotophic			
		Benthic			
		Brooder/ Direct developer			
		No larvae			
Life history	Life span	<1 year	Organism maximum life span as an adult	Community dynamics, resilience of organisms, reproduction, productivity	Beauchard et al. (2017); van der Linden et al. (2017); Degen and Faulwetter (2019); Liu et al. (2019)
		1–3 years			
		3–10 years			
	Reproductive frequency	Annual	Times that the organism reproduces over time	Demographic resilience, population stock	Beauchard et al. (2017); Degen and Faulwetter (2019)
		Continuous			
		Seasonal			
	Reproductive technique	Sexual, pelagic shed eggs	The mode organism reproduces, mechanism of fertilization & propagules released	Species dispersal, carbon transport, demographic resilience	Costello et al. (2015); Beauchard et al. (2017); Degen and Faulwetter (2019)
		Sexual, benthic shed eggs			
		Sexual, encapsulation (gelatinous mass)			
		Sexual, ovigerous, broad eggs			
		Sexual direct development			
		Asexual			

### 2.3 Trait allocation

Trait data were gathered from various published online sources, depending on the availability of information for each taxon. When trait information on a particular taxon was missing, its trait values were inferred from the nearest phylogenetic neighbor. For example, if no trait information was available at the species level, trait information was used from another species within the same genus; if information was unavailable at genus level, we considered information at family level. Additional considerations such as taxa distribution, resemblance, and expert judgment were also applied (see Tables S2 and S3).

### 2.4 Fuzzy coding of traits

Each of the taxa analyzed was scored depending on the affinity that a taxon displayed with a trait‐modality using a fuzzy coding procedure (Bremner, 2008; Bremner et al., 2006; Chevenet et al., 1994). A scoring range from 0 to 1 was used, with 0 being no affinity and 1 being high affinity to a trait. For example, coding the trait “Feeding mode” for *Aglaophamus australiensis* (Polychaeta), considered that *A. australiensis* is mostly a predatory species, however, it also exhibits some degree of subsurface deposit feeding, giving a fuzzy coding of 0.75 as predator, and 0.25 as subsurface deposit feeder, completing the full allocation of 1 for the feeding mode trait.

### 2.5 Case study: assessment of the SAMT database

To elucidate the utility of the SAMT database on the assessment of ecosystem functioning, a functional assessment encompassing four main regions across South Australia was performed. The regions selected were Coffin Bay (locality 1, 3, 4, and 6), Spencer Gulf (locality 9–10), Gulf St. Vincent (locality 14–17), and the Coorong (locality 28, 31–33) (Figure 1). For this case study, we only selected information on macrobenthic fauna from intertidal mudflats. Trait selection was made in the context of ecosystem functioning; thus, we analyzed only traits that influence the functioning of ecosystems (i.e., effect traits) that included, bioturbator, body size, feeding mode, morphology, living habit, and sediment position (Lam‐Gordillo et al., 2020).

Macrobenthic fauna were analyzed using both traditional biodiversity metric and functional approaches. The traditional biodiversity approaches included the analysis of taxonomic richness (S) and Simpson diversity index (1−λ) on macroinvertebrate abundances. For the functional approach, trait richness, Simpson index, and functional diversity (as Rao's quadratic entropy: RaoQ) were calculated on macroinvertebrate trait data. Diversity analyses and graphics were performed using R (R Core Team, 2017) and the packages “vegan” (Oksanen et al., 2019), “FD” (Laliberté et al., 2014), and “ggplot2” (Wickham, 2016). A univariate one‐factor PERMutational ANalysis Of VAriance (PERMANOVA) using Euclidean distance for the single variable (either effect traits, taxa‐ or trait‐based diversity index), permutation of residuals under a reduced model and 9,999 permutations was used to test for significant differences across regions (Anderson et al., 2008). All PERMANOVA tests were carried out using PRIMER v7 with PERMANOVA + add on.

## 3 RESULTS

### 3.1 Taxa included

**FIGURE 2:**
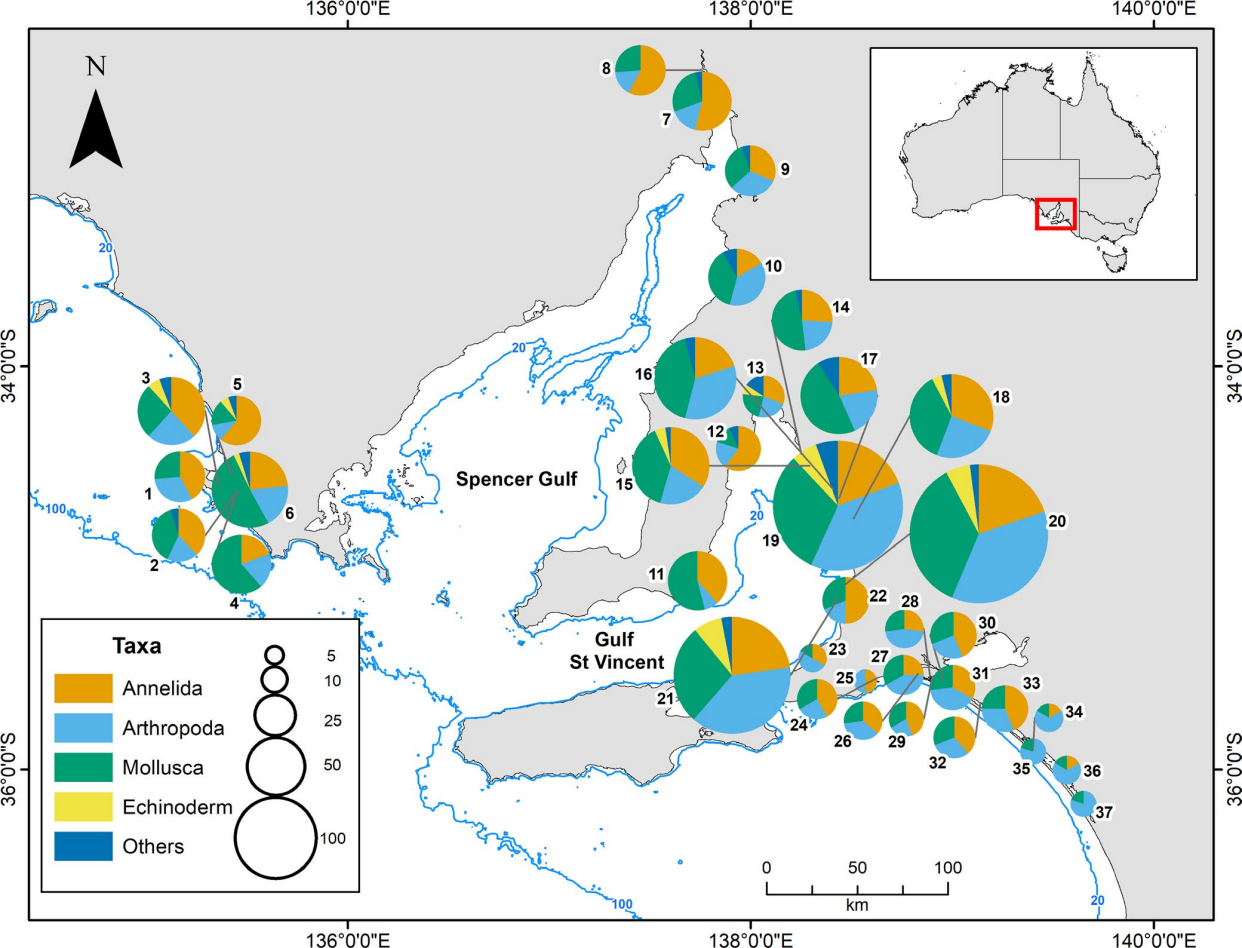
Number of taxa per locality of South Australia. Circle size is proportional to the number of taxa. (1) Port Douglas; (2) Eely Point; (3) Mount Dutton Bay; (4) Long Beach; (5) Crinoline Point; (6) Kellidie Bay; (7) Blanche Harbor; (8) Curlew Point; (9) Port Germain; (10) Fisherman Bay; (11) Coobowie; (12) Tiddy Widdy; (13) Port Arthur; (14) Port Parham; (15) Thompson's Beach; (16) Middle Beach; (17) Port Gawler; (18) Section Bank; (19) Glenelg; (20) Port Stanvac; (21) Port Noarlunga; (22) Onkaparinga; (23) Normanville; (24) Hindmarsh River; (25) Inman River; (26) Monument Rd; (27) Tarni Warra; (28) Hunters Creek; (29) Mundoo Channel; (30) Ewe Island; (31) Pelican Point; (32) Mulbin Yerrok; (33) Noonameena; (34) Parnka Point; (35) Villa de Yumpa; (36) Jack Point; (37) Loop Rd

In total, we generated trait information for 277 taxa (see Table S4 for a full list of taxa). The number of taxa varied (i.e., range from 4 to 142 per site, mean of 28) across the 37 localities of South Australia, with the greatest numbers from subtidal sediments in Gulf St Vincent (Figure S1). Different levels of taxonomic identification were assessed, 152 at the species level, followed by 28 at genus level, 86 at family level, and the remaining 11 taxa at higher levels (order, class, or phyla; Figure S2a). The phylum with most records was Mollusca (112 records, 40% of all taxa), followed by Arthropoda (94 records, 34% of all taxa) and Annelida (45 records, 16% of all taxa), with the remaining 10% belonging to other taxa (Echinodermata 15 taxa, one to three taxa each for Chordata, Sipunculida, Nemertea, Cnidaria, Porifera, and Brachiopoda; Figure S2b). Although Mollusca was the phylum with the highest number of records overall, Annelida was the phylum with the most records across localities (i.e., 43% of all sites) (Figure 2).

### 3.2 Data sources

**FIGURE 3:**
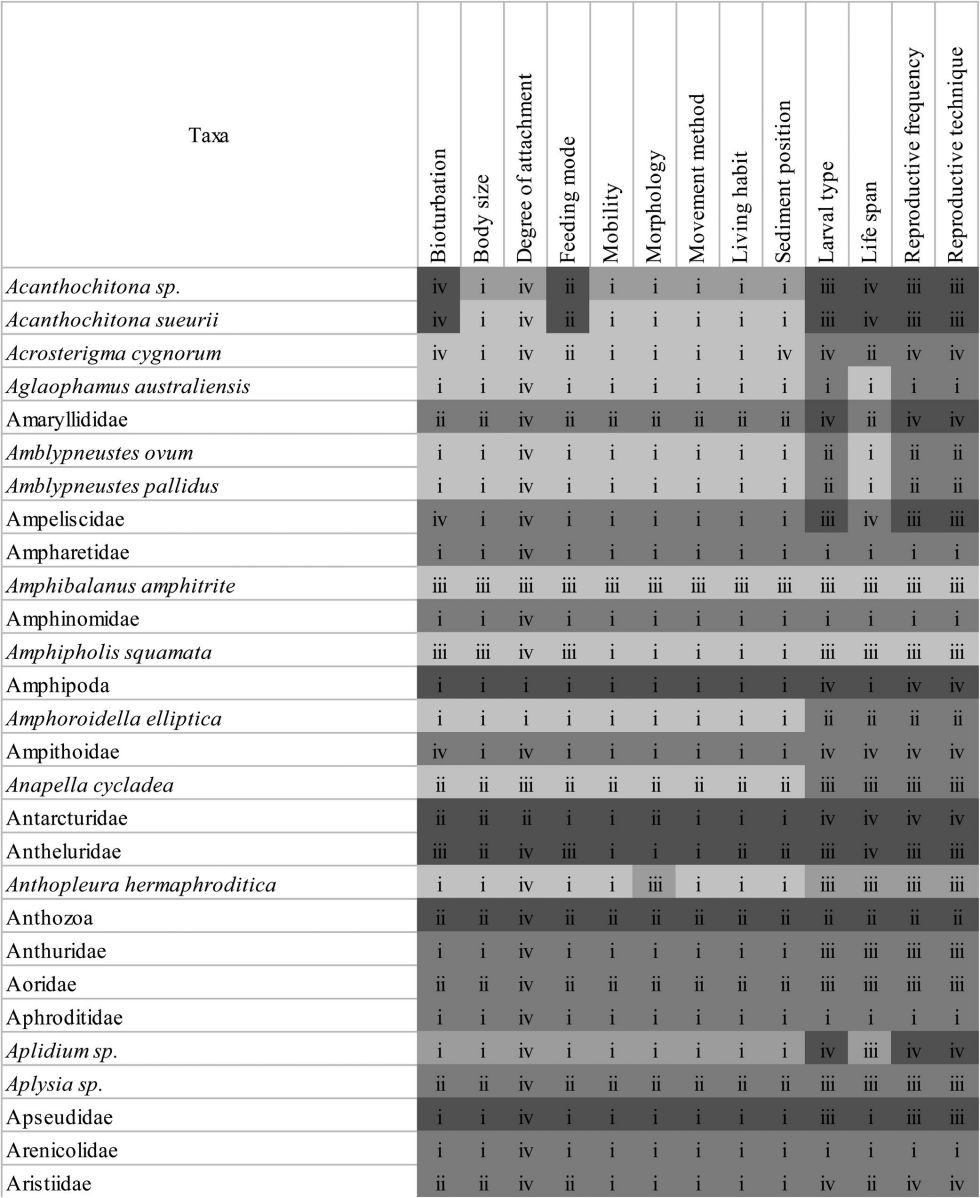
Screenshot of a section of the Traits information sources table. Roman numerals indicate sources’ origin, and cell shading specify the taxonomic level of the information. i: South Australian literature; ii: Australian literature; iii: Overseas literature; iv: online resources. Species level; Genus level; Family level; Order/Class level. Full table available in https://doi.org/10.6084/m9.figshare.12763154

**FIGURE 4:**
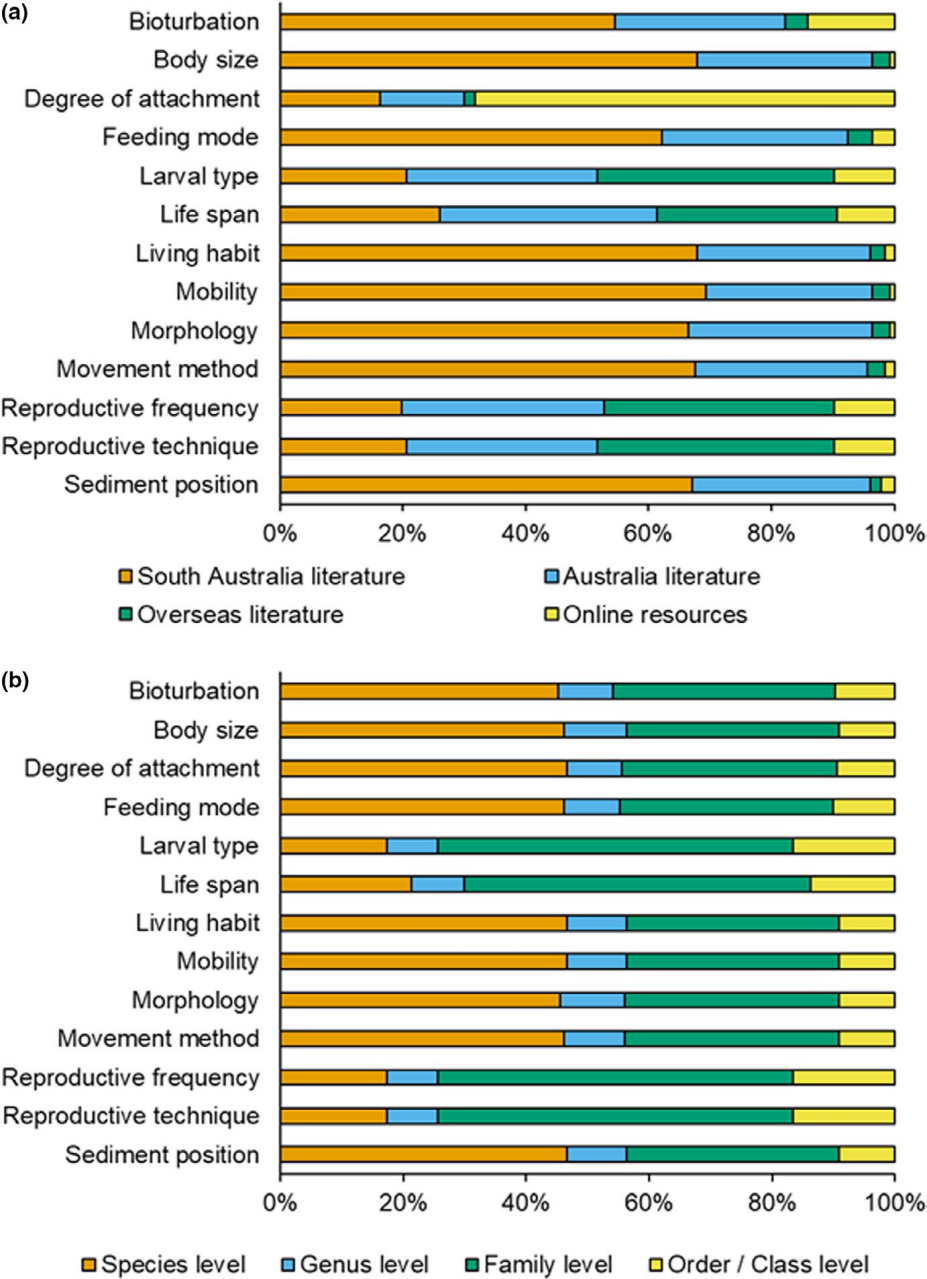
Stacked bar graphs showing (a) the cumulative percentage of trait information sources, and (b) the cumulative percentage of trait information by taxonomic level

The information on traits was retrieved from diverse peer reviewed and expert sources, and a database was generated for easy interpretation and useability (Figure 3; Trait source table in “https://doi.org/10.6084/m9.figshare.12763154”). Including all the traits assessed, 90% of the information was provided from primary literature that included 48% from South Australian literature, 29% from Australian literature, and 13% from overseas literature. The remaining 10% of information was obtained from reputable resources online (Table 2). However, the source of trait information varied between types of traits (Figure 4a). Across taxonomic levels, most of the trait information was available at the family (42%) and species (38%) levels, with proportionally less at the order/class and genus levels (11% and 9% respectively; Figure 4b). It also emerged that the traits larval type, life span, reproductive frequency, and technique are less studied for the macrobenthic fauna from Australia (Figure 4).

**TABLE 2:** Test results from univariate one‐way fixed factor PERMANOVA to compare trait expression of bioturbator, body size, feeding mode, morphology, living habit, and sediment position across regions. Significant results are shown in bold

	*df*	MS	Pseudo‐*F*	*p* (perm)
Bioturbator				
Region	3	995.08	8.3728	**.0009**
Residual	21	118.85		
Body size				
Region	3	1,635.6	9.8249	**.0011**
Residual	21	166.47		
Feeding mode				
Region	3	818.11	7.4907	**.0035**
Residual	21	109.22		
Morphology				
Region	3	1,115.00	7.0205	**.0023**
Residual	21	158.82		
Living habitat				
Region	3	1,136.70	9.0705	**.001**
Residual	21	125.32		
Sediment position				
Region	3	744.96	7.6826	**.0022**
Residual	21	96.97		

### 3.3 The South Australian Macrobenthic Traits (SAMT) database

**FIGURE 5:**
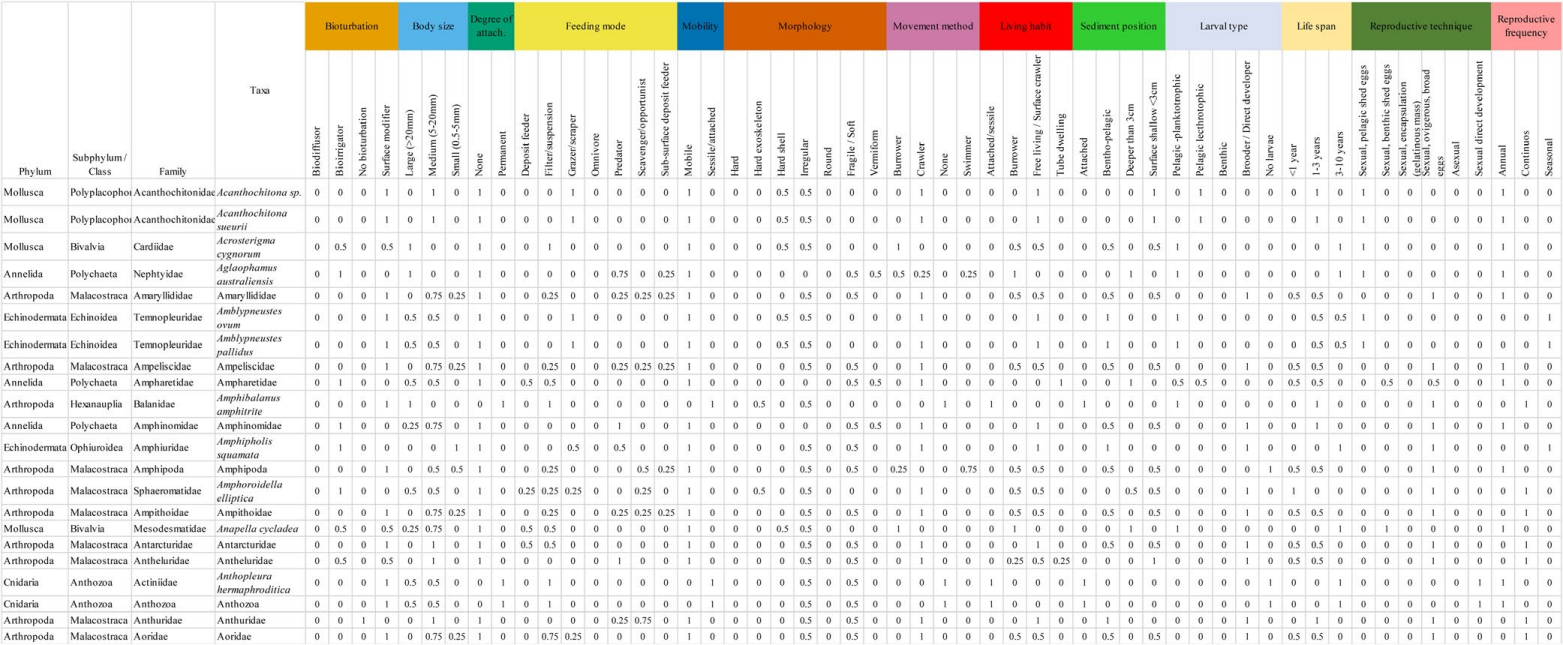
Screenshot of a section of the SAMT database (South Australia Macrobenthic Traits database). Traits are differentiated by colours. Phylum, Subphylum/ Class, Family and Taxa tabs are displayed for easy sorting and searching. Full table available in https://doi.org/10.6084/m9.figshare.12763154

Functional trait information (i.e., traits and fuzzy coding classification) for the 277 macrobenthic taxa analyzed from the South Australian region is the basis for the SAMT database, which is available as an accessible resource at “https://doi.org/10.6084/m9.figshare.12763154” (see Figure 5 for a screenshot of the SAMT database). Along with the database resource, version 1.0.0 of the SAMT R package is provided for assistance in using and analyzing the SAMT database. The SAMT v1.0.0 R package is currently available on the repository https://github.com/OrlandoLam/SAMT (see Appendix 1 for SAMT package user guide). The SAMT database is intended to progress with regular updates of new data by researchers conducting work across southern Australia for easy downloading and sharing.

**FIGURE 6:**
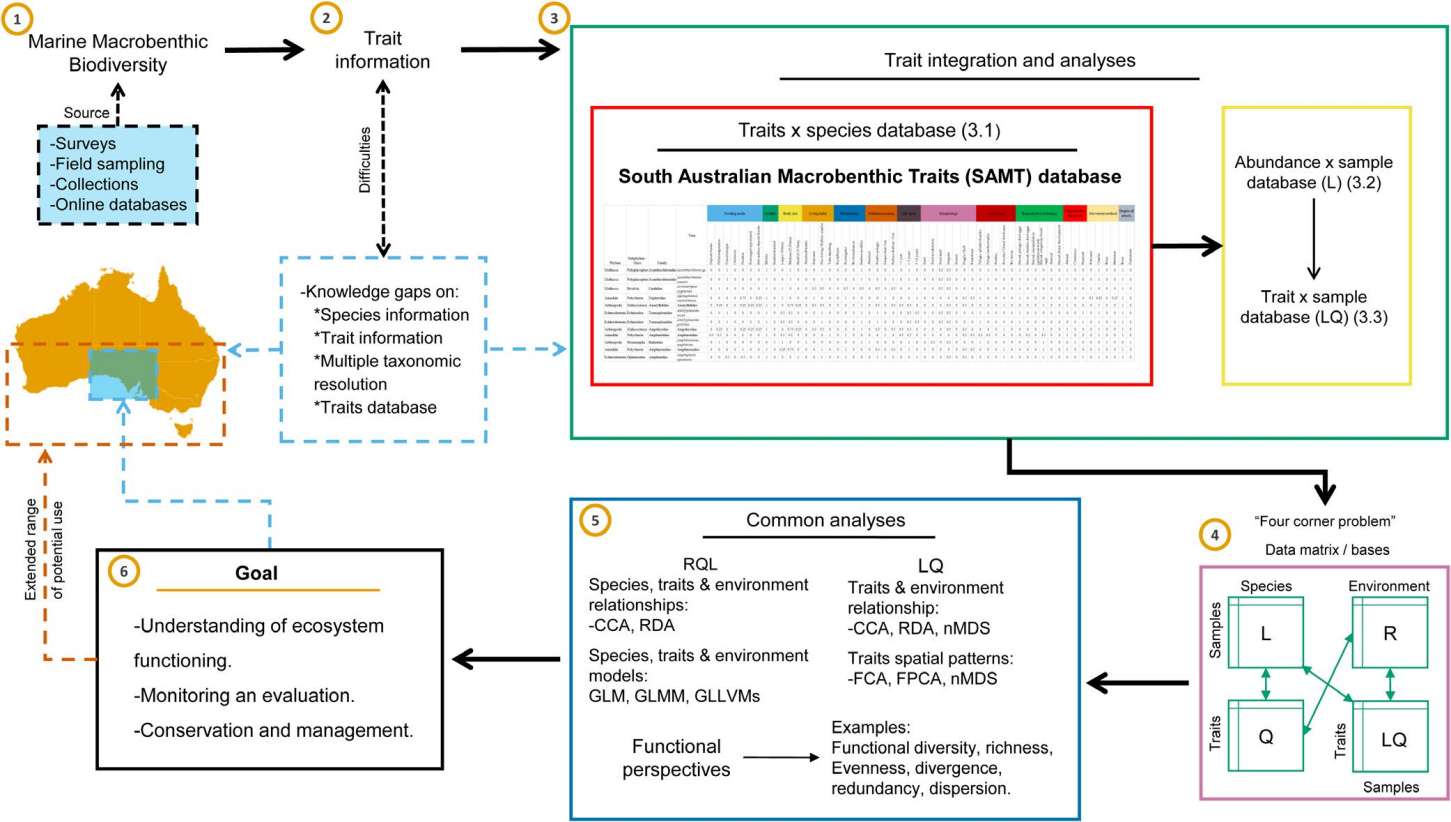
Flow chart showing step‐by‐step processes for assessing ecosystem functioning. Solid colored boxes (green, pink, blue, and black) represent the separate task for analyzing trait data, and black arrows indicate the logical order for the steps. Red box highlights the essential step for having a macrobenthic fauna trait database for southern Australia. Yellow box shows the complementary information needed. Blue dotted box and arrows show the information provided in this study, and the brown dotted box and arrow show the range of potential use of the information provided

To illustrate the utility of the SAMT database, we developed a flow chart showing the step‐by‐step process for assessing the contribution of macrobenthic fauna to ecosystem functioning (Figure 6). The first steps are to compile macrobenthic data from diverse sources (e.g., surveys, field sampling, collections, and online databases) and allocate the respective trait information to each taxon. The SAMT database reduces the time needed for gathering and finding the taxa‐trait information and provides the information in one place. Macrobenthic abundance data can be added to the database at any time, and the R package provided within SAMT database can be used for compiling a trait x sample matrix (LQ). Depending on the aim of the study, and with all the matrices compiled, different analyses can be performed using different software (e.g., R, PRIMER), from measuring trait patterns (LQ), relationships between species‐traits and the environment, or modeling the interactions between species‐traits and the environment (RQL), to calculating functional diversity as a proxy for assessing ecosystem functioning (Figure 6).

### 3.4 Case study using SAMT database: Preliminary functional perspectives for South Australia waters

**FIGURE 7:**
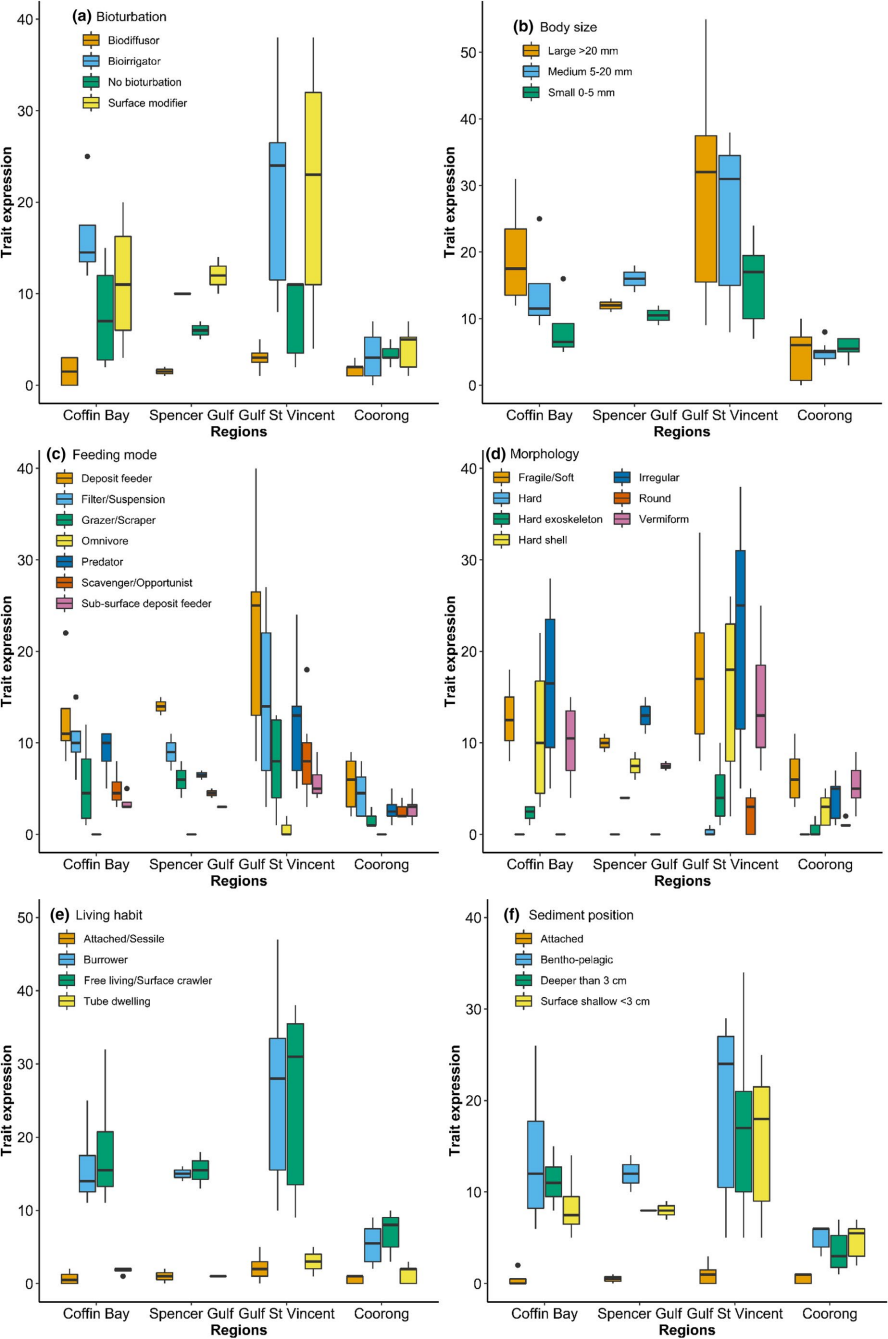
Expressed traits across four regions (Coffin Bay, Spencer Gulf, Gulf St. Vincent, and Coorong Lagoon) in South Australia. Median, percentiles, upper/lower bounds, and outliers are shown. Traits shown: (a) bioturbation, (b) body size, (c) feeding mode, (d) morphology, (e) living habit, and (f) sediment position

The analysis of data from the SAMT database included, on average, 47 of the 54 trait‐modalities across all taxa, analyzed across the 37 South Australian localities. However, based on the traits and localities analyzed, some trait‐modalities were expressed more than others due to the different number of taxa present in each locality. Based on effect traits, and grouping the localities into regions (e.g., Coffin Bay, Spencer Gulf, Gulf St. Vincent, and Coorong), the majority of the taxa recorded were surface modifiers and bioirrigators (Figure 7a), with large body size (Figure 7b) and were deposit feeders (Figure 7c). The most common morphology was irregular and fragile/soft bodies (Figure 7d). The most common living habit was free living/surface crawler and burrower (Figure 7e), and most of the organisms inhabited demersal habitats (Figure 7f).

Trait expression (i.e., the number of taxa that exhibit a determined trait) differed significantly across the regions (*p* < .01, Table 2). Considering the six effect traits analyzed (e.g., bioturbator, body size, feeding mode, morphology, living habit, and sediment position), Coffin Bay, Spencer Gulf, and Gulf St Vincent were significantly different in the number of traits present compared to the Coorong region (*p* < .01, Table 3). Greater similarities in terms of trait expression were found between Coffin Bay, Spencer Gulf, and Gulf St Vincent (Table 3).

**TABLE 3:** Results from univariate pairwise test of bioturbator, body size, feeding mode, morphology, living habit, and sediment position across regions. Significant results are shown in bold

Pairwise test	t	*p* (perm)
Bioturbator		
Coffin Bay, Spencer Gulf	0.77	.6047
Coffin Bay, Gulf St Vincent	1.11	.2997
Coffin Bay, Coorong	4.39	**.001**
Spencer Gulf, Gulf St Vincent	1.09	.3659
Spencer Gulf, Coorong	4.14	**.0106**
Gulf St Vincent, Coorong	4.78	**.0002**
Body size		
Coffin Bay, Spencer Gulf	0.85	.7364
Coffin Bay, Gulf St Vincent	1.32	.2027
Coffin Bay, Coorong	4.44	**.0006**
Spencer Gulf, Gulf St Vincent	1.22	.3086
Spencer Gulf, Coorong	4.35	**.0109**
Gulf St Vincent, Coorong	5.16	**.0001**
Feeding mode		
Coffin Bay, Spencer Gulf	0.42	.9292
Coffin Bay, Gulf St Vincent	1.17	.2447
Coffin Bay, Coorong	3.81	**.0006**
Spencer Gulf, Gulf St Vincent	0.93	.4901
Spencer Gulf, Coorong	3.81	**.0113**
Gulf St Vincent, Coorong	4.49	**.0002**
Morphology		
Coffin Bay, Spencer Gulf	0.57	.7966
Coffin Bay, Gulf St Vincent	0.86	.4178
Coffin Bay, Coorong	3.69	**.0003**
Spencer Gulf, Gulf St Vincent	1.07	.359
Spencer Gulf, Coorong	3.47	**.0114**
Gulf St Vincent, Coorong	4.50	**.0003**
Living habit		
Coffin Bay, Spencer Gulf	0.38	.9319
Coffin Bay, Gulf St Vincent	1.18	.2785
Coffin Bay, Coorong	4.32	**.0005**
Spencer Gulf, Gulf St Vincent	1.07	.3793
Spencer Gulf, Coorong	4.43	**.0105**
Gulf St Vincent, Coorong	5.01	**.0001**
Sediment position		
Coffin Bay, Spencer Gulf	0.50	.9332
Coffin Bay, Gulf St Vincent	1.13	.281
Coffin Bay, Coorong	3.91	**.0009**
Spencer Gulf, Gulf St Vincent	1.12	.3662
Spencer Gulf, Coorong	3.61	**.0111**
Gulf St Vincent, Coorong	4.61	**.0003**

**FIGURE 8:**
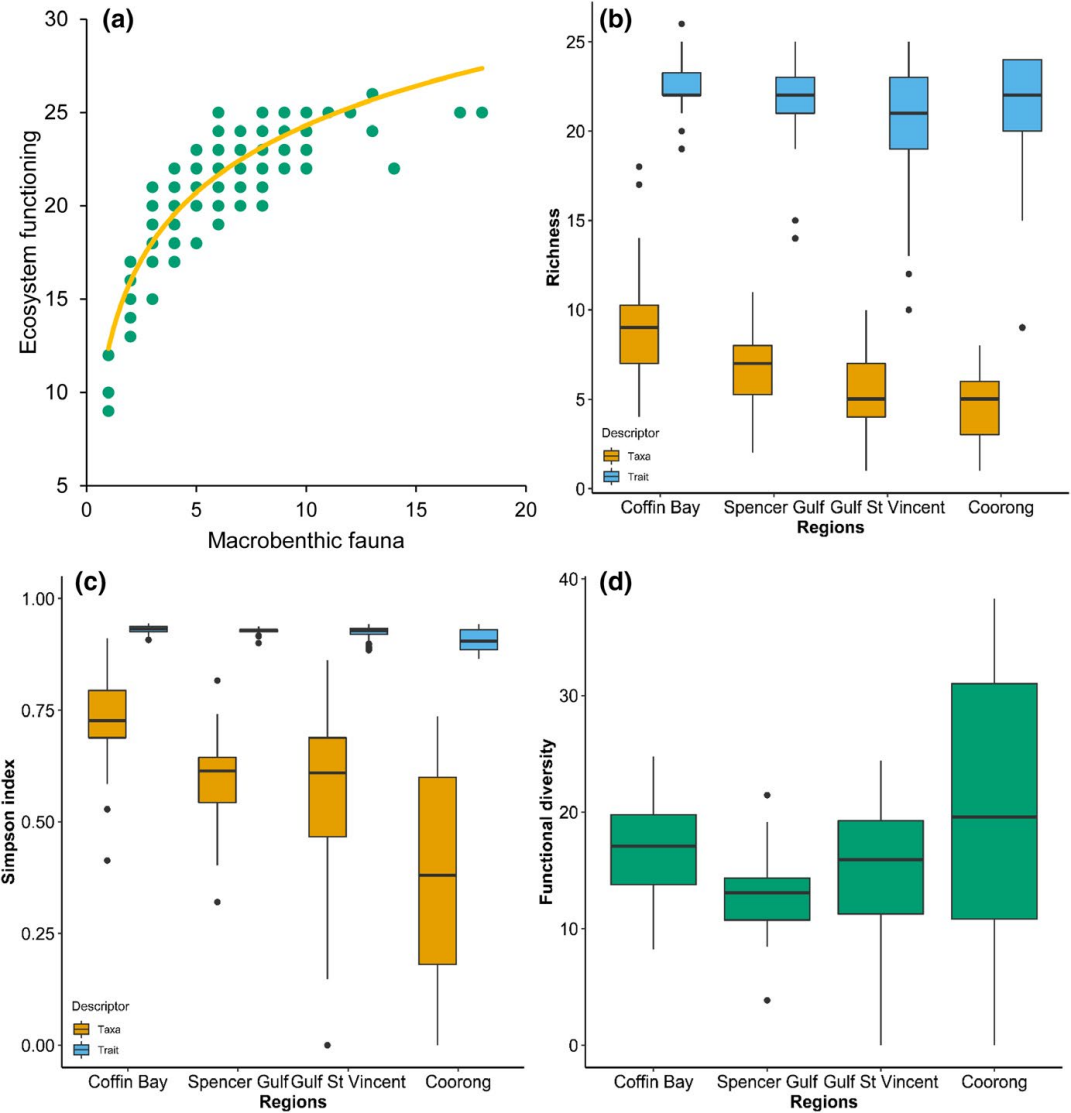
(a) Correlation and trend line between the macrobenthic fauna (number of taxa) and ecosystem functioning (trait expression) in the main four regions of South Australia. (b) Boxplots of the taxonomic and trait richness, (c) Simpson index, and (d) functional diversity across the four study regions

The relationship between the macrobenthic fauna (biodiversity) and trait expression (ecosystem functioning) was asymptotic, showing a decreasing effect of adding new species to the ecosystem (Figure 8a). Taxonomic and trait richness were significantly different across regions (*p* < .01, Table 4; Figure 8b). The pairwise tests revealed significant differences in taxa richness across all regions except for the pairing of the Gulf St Vincent and Coorong regions (*p* < .01, Table 5), while differences in trait richness were only identified between Coffin Bay and the other three regions (*p* < .01, Table 5). The example reveals that trait richness can show greater similarity, whereas macrobenthic fauna assemblages were taxonomically different between regions.

**TABLE 4:** Test results from univariate one‐way fixed factor PERMANOVA to compare Richness (S), Simpson index (1‐Lambda’), and functional diversity (FD) of macrobenthic fauna across regions. Significant results are shown in bold

	*df*	MS	Pseudo‐*F*	*p* (perm)
Richness (S)				
Taxa				
Region	3	140.32	23.803	**.0001**
Residual	203	5.89		
Trait				
Region	3	45.45	4.0587	**.0094**
Residual	203	11.20		
Simpson index				
Taxa				
Region	3	144.64	23.782	**.0001**
Residual	203	6.08		
Trait				
Region	3	0.004	21.85	**.0001**
Residual	203	0.0002		
Functional diversity				
Region	3	352.66	6.9265	**.0003**
Residual	202	50.91		

**TABLE 5:** Results from univariate pairwise test of richness (S), Simpson index (1‐Lambda’), and functional diversity (FD). Only significant differences are shown

Pairwise test	t	*p* (perm)
Richness (S)		
Taxa		
Coffin Bay, Spencer Gulf	3.15	.0028
Coffin Bay, Gulf St Vincent	7.34	.0001
Coffin Bay, Coorong	6.47	.0001
Spencer Gulf, Gulf St Vincent	3.04	.0036
Spencer Gulf, Coorong	3.51	.0010
Trait		
Coffin Bay, Spencer Gulf	2.34	.021
Coffin Bay, Gulf St Vincent	3.46	.001
Coffin Bay, Coorong	2.42	.017
Simpson index (1‐Lambda')		
Taxa		
Coffin Bay, Spencer Gulf	3.15	.0017
Coffin Bay, Gulf St Vincent	7.34	.0001
Coffin Bay, Coorong	6.48	.0001
Spencer Gulf, Gulf St Vincent	3.08	.0024
Spencer Gulf, Coorong	3.52	.0004
Trait		
Coffin Bay, Gulf St Vincent	2.30	.0232
Coffin Bay, Coorong	5.46	.0001
Spencer Gulf, Coorong	4.52	.0001
Gulf St Vincent, Coorong	6.09	.0001
Functional diversity (FD)		
Coffin Bay, Spencer Gulf	4.04	.0001
Spencer Gulf, Gulf St Vincent	2.06	.0400
Spencer Gulf, Coorong	3.01	.0038
Gulf St Vincent, Coorong	3.28	.0014

Diversity, measured using the Simpson Index (Figure 8c), revealed significant differences for taxa and traits across regions (*p* < .01, Table 4). Coffin Bay was the most significantly different region compared to the other regions based on both taxa and traits (Table 5). Based on traits, the Simpson Index was similar between Gulf St Vincent and Spencer Gulf. Based on taxa, the Simpson Index was significantly different between most region pairs except for the Gulf St Vincent and Coorong (Table 5). Functional diversity was also significantly different between regions (*p* < .01, Table 4, Figure 8d). In pairwise comparisons, functional diversity was different in Spencer Gulf compared to the other three regions, and in Gulf St Vincent compared to the Coorong (*p* < .05, Table 5). The case study demonstrated the usefulness of the SAMT database for elucidating functional similarities for taxonomically different benthic assemblages across regions.

## 4 DISCUSSION

Functional approaches have become a requisite for studying ecosystem functioning (e.g., Bolam et al., 2016; Bremner et al., 2003, 2006; Degen et al., 2018), yet, functional assessments remain hindered by a lack of taxa‐specific trait data (Lam‐Gordillo et al., 2020). Compiling trait information of marine macrobenthic fauna is often considered time‐consuming and difficult, due to knowledge gaps on the biology and ecology of many species, the lack of identification keys, as well as the scarcity of relevant data (Beauchard et al., 2017; Degen et al., 2018; Verissimo et al., 2012).

The SAMT database we present here aims to close the information gap by enabling a comprehensive assessment of traits for the South Australian macrobenthic fauna. SAMT, and the accompanying R package, will facilitate and enhance further research addressing ecosystem functioning and functional perspectives. The SAMT database provides trait information for 277 macrobenthic taxa and a trait classification for South Australian temperate marine waters. This first iteration of the SAMT database can be used as a part of the framework provided in this paper, with the aim to facilitate functional assessments along Australia's south coast.

The SAMT database is available for easy downloading, sharing, and using. However, as in any trait classification, several limitations need to be considered: (a) The structure of the database represents the current taxonomic classification at the time of the analysis, (b) the taxa included reflect the sampling design (e.g., effort, habitats sampled) of the projects from which the information was retrieved; and (c) the SAMT database is an ongoing project, with continuous updates and refinements as additional taxa and trait information becomes available, resulting in up to date versions of functional trait classifications.

We identified several knowledge gaps in the literature while building the SAMT database. For example, the majority of the information included for “Larval type” (58%, 160 of 277 taxa), “Reproduction technique” (58%, 160 of 277 taxa), “Reproduction frequency” (58%, 160 of 277 taxa), and “Life span” (56%, 156 of 277 taxa) were based on the family level taxonomic classification, highlighting that basic knowledge about macrobenthic fauna that inhabit southern Australian waters is still very limited in many cases.

The exemplary use of the SAMT database found an asymptotic pattern between the macrobenthic fauna taxa and trait expression (ecosystem functioning), which could be explained by redundancy in these regions. Redundancy can be due to (a) different species performing the same functioning in the ecosystem, and (b) adding species to the ecosystem until all functionality (functional traits) is represented (van der Linden et al., 2012; Loreau et al., 2002; Schulze & Mooney, 1993). Taxa and trait differences were found in terms of richness and diversity using the Simpson index across all regions, but for comparing particular regions, taxonomically indices varied more than those based on traits across all regions.

Functional diversity (FD), as Rao's quadratic entropy metric, was significantly different across regions, highlighting greater FD in the Coorong and the lowest FD in Spencer Gulf. This pattern could be explained by the Coorong region having the greatest abundance of individuals and the most similar community compared to the other regions, aligning with the properties of the Rao's quadratic entropy metric, that bases its calculations on the proportion of the abundance of taxa present and the measure of dissimilarities between them (Botta‐Dukát, 2005; Rao, 1982). The case study represents an example of the usefulness to combine both taxa and trait perspectives, as they give complementary insight to ecosystem functioning assessment and identify further research needs. Future targeted studies with consistent design can apply the database and framework presented here to demonstrate the ecological importance of effect traits and advance the understanding of the functionality of ecosystems along the southern Australian coast.

## 5 CONCLUSION

To date, this is the first study providing a comprehensive assessment of traits for the southern Australian macrobenthic fauna. We highlight that the South Australia Macrobenthic Traits (SAMT) database presented here is a valuable tool to enhance further research on trait‐based approaches within southern temperate Australia. The structure of the SAMT database includes 277 macrobenthic taxa so far, is very intuitive and was created for easy downloading, sharing, and using by researchers working on southern temperate benthic ecosystems. The newly developed R package for using and analyzing the SAMT database that can be applied more broadly to link trait and species data. A theoretical framework detailing the step‐by‐step process for assessing ecosystem functioning is introduced, illustrating the need for taxa‐trait information and the use of SAMT database.

The use of the SAMT database should be approached with awareness of its limitations of available taxonomic and trait‐based information, as well as ongoing changes to taxonomic nomenclature, traits information, and trait classification as the database evolves. The structure of the SAMT database will remain as simple as possible, avoiding complexity, redundancy, and duplication between traits as it expands to include more taxa, traits, and regions. The SAMT database is an ongoing project, where adding more taxa and traits will be continued with expansion into other regions within southern Australia.

## 6 CODE AVAILABILITY

Code is available on figshare (https://doi.org/10.6084/m9.figshare.12763154) and on the GitHub repository (https://github.com/OrlandoLam/SAMT).

## CONFLICT OF INTEREST

No potential conflict of interest was reported by the authors.

## AUTHOR CONTRIBUTION

**Orlando Lam‐Gordillo:** Conceptualization (lead); Data curation (lead); Formal analysis (lead); Investigation (equal); Methodology (lead); Software (lead); Validation (equal); Writing‐original draft (lead); Writing‐review & editing (equal). **Ryan Baring:** Conceptualization (supporting); Data curation (supporting); Formal analysis (supporting); Methodology (supporting); Supervision (lead); Validation (supporting); Writing‐original draft (supporting); Writing‐review & editing (equal). **Sabine Dittmann:** Conceptualization (supporting); Data curation (supporting); Formal analysis (supporting); Methodology (supporting); Supervision (lead); Validation (supporting); Writing‐original draft (supporting); Writing‐review & editing (equal).

## Supporting information

Supplementary Material

Supplementary Material

## Data Availability

Data are available on figshare (https://doi.org/10.6084/m9.figshare.12763154).
