# Supplementary material for: Establishing the South Australian Macrobenthic Traits (SAMT) database: A trait classification for functional assessments
Source: Ecol Evol. 2020 Nov 27;10(24):14372–87. doi: 10.1002/ece3.7040 (PMC7771161; doi:10.1002/ece3.7040)
Supplement: Supplementary file 1 — Supplementary Material [file ECE3-10-14372-s002.docx]

**List of Supplementary Material**

**Table S1.** Summary information of the taxa datasets used for The South Australian Macrobenthic Traits database. n: number of samples.

| Localities | Zone | | Habitat | | n |
| --- | --- | --- | --- | --- | --- |
|  | Intertidal | Subtidal | Mudflat | Mussel beds |  |
| Port Douglas |  |  |  |  |  |
| Eely Point |  |  |  |  |  |
| Mount Dutton Bay |  |  |  |  |  |
| Long Beach | x |  | x |  | 15 |
| Crinoline Point | x |  |  | x | 6 |
| Kellidie Bay | x |  | x |  | 15 |
| Blanche Harbor | x |  |  | x | 6 |
| Curlew Point | x |  |  | x | 6 |
| Port Germein | x |  | x |  | 15 |
| Fisherman Bay | x |  | x |  | 15 |
| Coobowie | x |  |  | x | 12 |
| Tiddy Widdy | x |  | x |  | 13 |
| Port Arthur | x |  |  |  | 15 |
| Port Parham | x |  | x |  | 15 |
| Thompson's Beach | x |  | x |  | 20 |
| Middle Beach | x |  | x |  | 35 |
| Port Gawler | x |  | x | x | 36 |
| Section Bank | x |  | x | x | 54 |
| Glenelg |  | x | x |  | 200 |
| Port Stanvac |  | x | x |  | 200 |
| Port Noarlunga |  | x | x |  | 200 |
| Onkaparinga | x |  | x |  | 30 |
| Normanville | x |  | x |  | 12 |
| Hindmarsh River | x |  | x |  | 18 |
| Inman River | x |  | x |  | 10 |
| Monument Rd | x |  | x |  | 30 |
| Tarni warra | x |  | x |  | 10 |
| Hunters Creek | x |  | x |  | 30 |
| Mundoo Channel | x |  | x |  | 30 |
| Ewe Island | x |  | x |  | 30 |
| Pelican Point | x |  | x |  | 45 |
| Mulbin Yerrok | x |  | x |  | 30 |
| Noonameena | x |  | x |  | 45 |
| Parnka Point | x |  | x |  | 30 |
| Villa de Yumpa | x |  | x |  | 30 |
| Jack Point | x |  | x |  | 30 |
| Loop Rd | x |  | x |  | 30 |

**Table S2.** Bibliographic references where trait information by taxa was retrieved.

| **Taxa** | **References** |
| --- | --- |
| *Acanthochitona sp.* | Glynn 1970; Edgar 2008; Gowlett-Holmes 2008. |
| *Acanthochitona sueurii* | Glynn 1970; Edgar 2008; Gowlett-Holmes 2008. |
| *Acrosterigma cygnorum* | Lamprell & Whitehead 1992; Gowlett-Holmes 2008; BIOTIC (online resource). |
| *Aglaophamus australiensis* | Fauchald 1965; Rainer 1977; Beesley et al. 2000; |
| Amaryllididae | Lowry & Stoddart 2002; Crustacea.net (online resource). |
| *Amblypneustes ovum* | Williamson & Steinber 2002; Gowlett-Holmes 2008; O'Hara & Byrne 2017. |
| *Amblypneustes pallidus* | Williamson & Steinber 2002; Gowlett-Holmes 2008; O'Hara & Byrne 2017. |
| Ampeliscidae | Lowry & Poore 1985, King 2009, Durkina et a. 2017; Crustacea.net (online resource). |
| Ampharetidae | Beesley et al. 2000 |
| *Amphibalanus amphitrite* | Satheesh & Wesley 2009; Burden et al. 2014. |
| Amphinomidae | Beesley et al. 2000 |
| *Amphipholis squamata* | Unno 2000; Boissin et al. 2010; BIOTIC (online resource). |
| Amphipoda | Hale 1927; Gowlett-Holmes 2008; Crustacea.net (online resource). |
| *Amphoroidella elliptica* | Hale 1927; Bruce 2003; Crustacea.net (online resource). |
| Ampithoidae | Poore & Lowry 1997; Peart 2007a; Peart 2007b; Hughes & Peart 2013; Crustacea.net (online resource). |
| *Anapella cycladea* | Lamprell & Whitehead 1992; Edgar 2008; Herrmann et al. 2009. |
| Antarcturidae | Hale 1927; Poore 2001; Poore 2015; Crustacea.net (online resource). |
| Antheluridae | Hale 1927; Poore & Lewton 1998; Crustaceana.net (online resource). |
| *Anthopleura hermaphroditica* | Edgar 2008; Spano et al. 2013; Spano & Haussermann 2017. |
| Anthozoa | Edgar 2008. |
| Anthuridae | Hale 1927; Cadien & Brusca 1993; Crustacea.net (online resource). |
| Aoridae | Hale 1927; Drake & Arias 1995; Myers 2005; Hughes 2017; Crustacea.net (online resource). |
| Aphroditidae | Beesley et al. 2000. |
| *Aplidium sp.* | Edgar 2008; Gowlett-Holmes 2008. |
| *Aplysia sp.* | Angeloni et al. 2002; Gowlett-Holmes 2008; Lee et al. 2014; Nimbs et al. 2017. |
| Apseudidae | Hale 1927; Blazwicz-Paszkowycz & Bamber 2007a; Blazwicz-Paszkowycz & Bamber 2007b; Rumbold et al. 2012; Ferreira et al. 2015; Rumbold et al. 2015; Crustacea.net (online resources). |
| Arenicolidae | Beesley et al. 2000. |
| Aristiidae | Hale 1927; Stoddart & Lowry 2010; Crustaceana.net (online resource). |
| *Arthritica semen* | Wells & Threlfall 1982; Jaspersen & Lutzen 2009. |
| *Ascidia sp.* | Edgar 2008; Gowlett-Holmes 2008. |
| Ascidiacea | Edgar 2008; Gowlett-Holmes 2008. |
| *Atactodea cuneata* | Hooker & Creese 1995a; Hooker & Creese 1995b; Lamprell & Whitehead 1992; https://researchspace.auckland.ac.nz/handle/2292/5758; https://www.biodiversitylibrary.org/item/28580#page/10/mode/1up. |
| *Australonereis ehlersi* | Dorsey 1981; Beesley et al. 2000; De Roach 2006. |
| *Australostichopus mollis* | Slater 2009; Zamora & Jeffs 2013 |
| *Austrocochlea constricta* | Underwood & Creese 1976; Rintala 2014 https://www.gbri.org.au/SpeciesList/AustrocochleaConstricta%7CAidanRintala.aspx?PageContentID=5174. |
| *Austrominius adelaidae* | Jones 2012. |
| *Balanus sp.* | Edgar 2008; Gowlett-Holmes 2008; Thiyagarajan et al. 2005 |
| *Bassina sp.* | Lamprell & Whitehead 1992. |
| *Bedeva paivae* | Black 1976; Gowlett-Holmes 2008. |
| *Bedeva vinosa* | Black 1976; Gowlett-Holmes 2008. |
| *Bellidilia laevis* | Hale 1927; Poore 2004; Gowlett-Holmes 2008. |
| *Bellidilia undecimspinosa* | Hale 1927; Poore 2004; Gowlett-Holmes 2008. |
| *Bembicium nanum* | Underwood 1975; Edgar 2008; Gowlett-Holmes 2008; https://seashellsofnsw.org.au/Littorinidae/Pages/Bembicium_nanum.htm. |
| *Bembicium vittatum* | Edgar 2008; Gowlett-Holmes 2008; online resource. |
| *Biffarius arenosus* | Hale 1927; Poore 2004; Stapleton et al. 2001; Butler et al. 2009. |
| *Biffarius limosus* | Hale 1927; Poore 2004. |
| *Boccardiella limnicola* | Beesley et al. 2000. |
| *Brachidontes rostratus* | Lamprell & Whitehead 1992; Gowlett-Holmes 2008; El-Deeb et al. 2018; El-Sayed et al. 2018. |
| *Brachidontes sp.* | Lamprell & Whitehead 1992; Gowlett-Holmes 2008; El-Deeb et al. 2018; El-Sayed et al. 2018. |
| *Brachynotus spinosus* | Hale 1927; Griffin 1969; Griffin1971; Poore 2004. |
| *Bulla quoyii* | Lamprell & Whitehead 1992; Gowlett-Holmes 2008; Malaquias et al. 2008. |
| *Byblis sp.* | Lowry & Poore 1985, King 2009, Durkina et a. 2017; Crustacea.net (online resource). |
| *Cacozeliana granarium* | Murray 1969; Gowlett-Holmes 2008. |
| *Cadulus vincentianus* | Lamprell & Healy 1998. |
| Callianassidae | Hale 1927; Poore 2004. |
| *Callista kingii* | Lamprell & Whitehead 1992; Beaver et al. 2016. |
| Capitellidae | Petraitis 1985; Beesley et al. 2000. https://naturalhistory2.si.edu/smsfp/irlspec/Capitella_capitata.htm |
| *Caprella danilevskii* | Hale 1927; Takeuchi & Hirano 1991; Crustacea.net (online resource). |
| Caprellidae | Hale 1927; Bynum 1978; Gowlett-Holmes 2008; Takeuchi & Hirano 1991; De Paula et al. 2016; Crustacea.net (online resource). |
| Cardiidae | Lamprell & Whitehead 1992; Edgar 2008; ter Poorten et al. 2017. |
| *Cardita crassicosta* | Lamprell & Whitehead 1992. |
| Caridea | Hale 1927; Poore 2004; Gowlett-Holmes 2008. |
| *Carpoapseudes austroafricanus* | Hale 1927; Blazwicz-Paszkowycz & Bamber 2007a; Blazwicz-Paszkowycz & Bamber 2007b; Rumbold et al. 2012; Ferreira et al. 2015; Rumbold et al. 2015; Crustacea.net (online resources). |
| *Cassidinopsis lacertosa* | Hale 1927; Bruce 2003; Crustacea.net (online resource). |
| *Cellana tramoserica* | Fletcher 1984: Bulleri et al. 2004. |
| *Centrocardita rosulenta* | Lamprell & Whitehead 1992. |
| Ceratocumatidae | Hale 1927. |
| Ceratopogonidae | Mullen & Hribar 1988; Dourado et al. 2017; https://www.mdfrc.org.au/bugguide/index.htm. |
| *Cerceis tridentata* | Hale 1927; Bruce 2003; Crustacea.net (online resource). |
| Cerithiidae | Murray 1969; Gowlett-Holmes 2008. |
| *Chama sp.* | Lamprell & Whitehead 1992; Gowlett-Holmes 2008. |
| *Chamaesipho tasmanica* | Jeffery 1997; Jeffery & Underwood 2001; Edgar 2008; Gowlett-Holmes 2008. |
| Chironomidae larvae | Coffman & Ferrington 1996 in Merritt & Cummins 1996; Foote 1987 in Stehr 1987; https://www.mdfrc.org.au/bugguide/index.htm; https://animaldiversity.org/accounts/Chironomidae/ |
| *Chitonopsis spatulifrons* | Hale 1927; Bruce 2003; Crustacea.net (online resource). |
| *Chondrochelia ignota* | Hale 1927; Blazwicz-Paszkowycz & Bamber 2007a; Blazwicz-Paszkowycz & Bamber 2007b; Rumbold et al. 2012; Ferreira et al. 2015; Rumbold et al. 2015; Crustacea.net (online resources). |
| *Cirolana cranchii* | Hale 1927; Wong & Moore 1996. |
| Cirratulidae | Beesley et al. 2000. |
| *Cirriformia sp.* | Beesley et al. 2000. |
| *Clanculus dunkeri* | Jansen 1993; Gowlett-Holmes 2008. |
| *Clanculus limbatus* | Jansen 1993; Gowlett-Holmes 2008. |
| *Clanculus philippi* | Jansen 1993; Gowlett-Holmes 2008. |
| *Clanculus plebejus* | Jansen 1993; Gowlett-Holmes 2008. |
| *Cominella lineolata* | Edgar 2008; Gowlett-Holmes 2008; https://collections.museumvictoria.com.au/species/8722 |
| Condylocardiinae | Lamprell & Whitehead 1992; Middelfart 2002. |
| *Conuber conicum* | Kingsley-Smith et al. 2005; Richardson et al. 2005; Kulikova et al. 2007; Gowlett-Holmes 2008; Huelsken et al. 2008; https://seashellsofnsw.org.au/Naticidae/Pages/Conuber_conicum.htm; https://molluscsoftasmania.org.au/project/conuber-conicum/ |
| Corophiidae | Hale 1927; Prato & Biandolino 2006; Myers 2009; Crustacea.net (online resource). |
| *Crabyzos longicaudatus* | Hale 1927; Poore & Ton 1993; Edgar 2008; Gowlett-Holmes 2008. |
| *Cryptocnemus vincentianus* | Hale 1927; Lam-Gordillo et al. 2019. |
| Cumacea | Hale 1927; Gerken 2001; Akiyama & Yamamoto 2004; Gerken 2013; Gerken 2014. |
| *Cyclaspis spilotes* | Hale 1927; Gerken 2001; Gerken 2013. |
| *Cyclaspis tribulis* | Hale 1927; Gerken 2001; Gerken 2013. |
| Cyproideidae | Hale 1927; Barnard & Karman 1991; Lowry & Azman 2008; Azman 2009; Crustacea.net (online resource). |
| Dexaminidae | Hale 1927; Barnard & Karman 1991; Crustacea.net (online resource). |
| Diastylidae | Hale 1927; Gerken 2014. |
| Dolichopodidae larvae | LaSalle & Bishop 1990; Cicero et al. 2017; https://www.mdfrc.org.au/bugguide/index.htm. |
| Dorvilleidae | Beesley et al. 2000. |
| *Dosinia sp.* | Lamprell & Whitehead 1992; Nie et al. 2016. |
| *Duplicaria kieneri* | Miller 1975; https://seashellsofnsw.org.au/Terebridae/Pages/Terebridae_intro.htm; http://www.gastropods.com/5/Shell_4905.shtml |
| *Ebalia intermedia* | Hale 1927; Poore 2004; Gowlett-Holmes 2008. |
| *Electroma papilionacea* | Gowlett-Holmes 2008; https://molluscsoftasmania.org.au/project/electroma-papilionacea/ |
| *Eoacmaea calamus* | Edgar 2008; Gowlett-Holmes 2008; online resource. |
| *Epitonium tenerum* | Kilburn 1985; Huang & Lee 2016. |
| *Equichlamys bifrons* | Lamprell & Whitehead 1992; Wolf & White 1995; Gowlett-Holmes 2008. |
| *Euchone variabilis* | Hutchings and Murray 1984; Beesley et al. 2000. |
| *Euidotea bakeri* | Hale 1927; Edgar 2008; Gowlett-Holmes 2008; https://collections.museumvictoria.com.au/species/13606; https://www.sealifebase.se/summary/Euidotea-bakeri.html |
| Eunicidae | Beesley et al. 2000. |
| Euphrosinidae | Beesley et al. 2000. |
| Eusiridae | Hale 1927; Smith & Williams 1983; Barnard & Karman 1991; Crustacea.net (online resources). |
| *Exosphaeroma alii* | Hale 1927; Wall et al. 2015; Crustacea.net (online resources). |
| *Exosphaeroma bicolor* | Hale 1927; Wall et al. 2015; Crustacea.net (online resources). |
| *Ficopomatus enigmaticus* | Dew 1959; Beesley et al. 2000; Styan et al. 2017. |
| Fissurellidae | Creese 1981; Aktipis et al. 2010; Gowlett-Holmes 2008; Leon-Cisneros et al. 2017. |
| Flabelligeridae | Beesley et al. 2000. |
| *Galathea australiens* | Hale 1927; Poore 2004; Gowlett-Holmes 2008. |
| Gammaridae | Hale 1927; Barnard & Karman 1991; Subida et al. 2005; Crustacea.net (online resource). |
| *Gazameda iredalei* | Marwick & Hutt 1956; Gowlett-Holmes 2008; Allmon 2011. |
| Glyceridae | Beesley et al. 2000. |
| *Glycymeris radians* | Iredale 1929; Gowlett-Holmes 2008; Peharda et al. 2013. |
| *Gnathia mulieraria* | Hale 1927; Cohen & Poore 1994; Tanaka 2007; Crustacea.net (online resource). |
| *Golfingia* sp. | Murina 1984; Beesley et al. 2000; Endmonds 2000; Edgar 2008; Gowlett-Holmes 2008; Adrianov & Maiorova 2010; Rice & Pilger ND. |
| *Gomeza bicornis* | Hale 1927; Poore 2004. |
| Goniadidae | Beesley et al. 2000. |
| *Granata sp.* | Gowlett-Holmes 2008; Herbet 2012. |
| *Gynodiastylis truncatifrons* | Hale 1927; Gerken 2001. |
| *Halicarcinus ovatus* | Hale 1927; Griffin & Yaldwyn 1971; Lucas 1972; Poore 2004; Gowlett-Holmes 2008. |
| *Haloniscus searlei* | Hale 1927; Bayly & Ellis 1969; Ellis & Williams 1969; Williams 1983; Blin et al. 1989. |
| *Haustorius sp.* | Hale 1927; Dennell 1932; Croker 1967; Kamihira 1981; Highsmith & Coyle 1991. |
| *Helograpsus haswellianus* | Poore 2004; Breitfuss et al. 2004; Gowlett-Holmes 2008; Katrak et al. 2008; Katrak & Dittmann 2011. |
| *Heteroserolis australiensis* | Hale 1927; Harrison & Poore 1984. |
| *Hiatella australis* | Edgar 2008; Gowlett-Holmes 2008; van der Molen et al. 2007. |
| *Hiatula alba* | Lamprell & Whitehead 1992; Matthews & Fairweather 2003; Matthews & Fairweather 2004; Gowlett-Holmes 2008. |
| *Holopneustes purpurascens* | Williamson & Steinberg 2002; Swanson et al. 2006. |
| *Holothuria (Panningothuria) austrinabassa* | Tuwo & Conand 1992; O'loughlin et al. 2007; Gowlett-Holmes 2008; Benitez-Villalobos et al. 2013. |
| Holothuroidea | Tuwo & Conand 1992; O'loughlin et al. 2007; Gowlett-Holmes 2008; Benitez-Villalobos et al. 2013. |
| Hyalidae | Lowry 1980; Tsoi 1999; Tsoi & Chu 2005; Crustacea.net (online resource). |
| Hydrobiidae | Ponder et al. 1991; Kabat and Hershler 1993; Ponder et al. 1999. |
| Isaeidae | Myers 1995; Weslawski & Legezynska 2002. |
| *Ischnochiton sp.* | Edgar 2008; Gowlett-Holmes 2008; Grayson & Chapman 2004; Liversage & Benkendorff 2017. |
| *Ischnochiton variegatus* | Smith & Robertson 1970; Edgar 2008; Gowlett-Holmes 2008; online resource. |
| Janiridae | Hale 1927; Hessler & Stromberg 1989; Wilson & Wagele 1994; Linse et al. 2014; Crustacea.net (online resource). |
| *Katelysia peronii* | Nielsen 1963; Roberts 1984; Lamprell & Whitehead 1992; Bellchambers 1998; Cantin 2010; Dent et al. 2016. |
| *Katelysia rhytiphora* | Nielsen 1963; Roberts 1984; Lamprell & Whitehead 1992; Bellchambers 1998; Edgar 2008; Dent et al. 2016. |
| *Katelysia scalarina* | Nielsen 1963; Roberts 1984; Lamprell & Whitehead 1992; Bellchambers 1998; Edgar 2008; Cantin 2010; Dent et al. 2016. |
| *Laternula sp.* | Lamprell & Whitehead 1992; Ahn 1993; Philipp et al. 2008; Kang et al. 2009. |
| Leptocheliidae | Hale 1927; Blazwicz-Paszkowycz & Bamber 2012; Rumbold et al. 2012; Ferreira et al. 2015; Rumbold et al. 2015; Crustacea.net (online resource). |
| Leucosoleniidae | Edgar 2008; Gowlett-Holmes 2008; Borojevic et al. 2000. |
| *Liloa brevis* | Burn 2006; Saunders 2009; Too et al. 2014; Nimbs & Smith 2016. |
| *Limaria orientalis* | Lamprell & Whitehead 1992; Gowlett-Holmes 2008; online resource. |
| *Limatula strangei* | Flemming 1977; Lamprell & Whitehead 1992; Gowlett-Holmes 2008. |
| *Litocheira bispinosa* | Hale 1927; Griffin & Yaldwyn 1971; Poore 2004; Gowlett-Holmes 2008. |
| *Litogynodiastylis turgida* | Hale 1927; Gerken 2001. |
| *Lophopagurus (Lophopagurus) nanus* | Hale 1927; Poore 2004; Wada et al. 2000; Mantelatto et al. 2007; Gowlett-Holmes 2008; Korn et al. 2018; Kornienko et al. 2019. |
| Lottiidae | Nakano & Ozawa 2005; Edgar 2008; Gowlett-Holmes 2008. |
| Lucinidae | Lamprell & Whitehead 1992; Glover & Taylos 2001; Gowlett-Holmes 2008; Taylor et al. 2011. |
| Lumbrineridae | Beesley et al. 2000. |
| *Lutraria rhynchaena* | Lamprell & Whitehead 1992; Gowlett-Holmes 2008; Gan et al. 2016. |
| Lysianassidae | Hale 1927; Sainte-Marie 1986; Lowry & Stoddart 1995; Kilgallen & Lowry 2013. |
| *Macomona deltoidalis* | Ponder 1975; Lamprell & Whitehead 1992; https://molluscsoftasmania.org.au/project/macomona-deltoidalis/ |
| Mactridae | Healy & Lamprell 1992; Lamprell & Whitehead 1992; Gowlett-Holmes 2008. |
| *Mactrotoma antecedens* | Healy & Lamprell 1992; Lamprell & Whitehead 1992. |
| *Magellania flavescens* | Edgar 2008; Gowlett-Holmes 2008; Baird et al. 2013. |
| Magelonidae | Beesley et al. 2000. |
| Maldanidae | Beesley et al. 2000. |
| *Mesanthura maculata* | Hale 1927; Poore & Ton 1986; Cadien & Brusca 1993; Crustacea.net (online resource). |
| *Metapenaeopsis novaeguineae* | Hale 1927; Poore 2004; Gowlett-Holmes 2008. |
| *Mimachlamys sp.* | Iredale 1929; Lamprell & Whitehead 1992; Zacharin 1995; Gowlett-Holmes 2008; Dijkstra & Beu 2018. |
| *Mitrella australis* | <https://seashellsofnsw.org.au/Columbellidae/Pages/Columbellidae_intro.htm> |
| *Mitrella lincolnensis* | <https://seashellsofnsw.org.au/Columbellidae/Pages/Columbellidae_intro.htm> |
| Mopaliidae | Edgar 2008; Gowlett-Holmes 2008; Vendrasco et al. 2008; Lord 2011; Sigwart et al. 2013. |
| *Musculus nana* | Gowlett-Holmes 2008; Ab Rahim et al. 2016. |
| *Myadora albida* | Healy et al. 2015. |
| *Mysella sp.* | Passos et al. 2004. |
| Mysidae | Fenton 1986; Panampunnayil 1986; Jocque & Blom 2009; Lill et al. 2010. |
| Nassariidae | McKillup & Butler 1979; Chan & Morton 2005; Edgar 2008; Gowlett-Holmes 2008. |
| *Nassarius pauperatus* | McKillup & Butler 1979; Chan & Morton 2005; Edgar 2008; Gowlett-Holmes 2008. |
| *Nassarius pyrrhus* | Chan & Morton 2005; Edgar 2008; Gowlett-Holmes 2008. |
| *Natatolana vieta* | Hale 1927; Bruce 1986; Keable 2006; Crustacea.net (online resource). |
| *Natica sp.* | Kingsley-Smith et al. 2005; Richardson et al. 2005; Kulikova et al. 2007; Edgar 2008; Gowlett-Holmes 2008; Huelsken et al. 2008. |
| *Neanthes vaalii* | Beesley et al. 2000; Baken 2002. |
| *Neastacilla deducta* | Hale 1927; King 2003; Crustacea.net (online resource). |
| Nebaliidae | Hale 1927; Vetter 1996; Walker-Smith & Poore 2001. |
| Nemertea | Egan & Anderson 1979; Edgar 2008; Gowlett-Holmes 2008. |
| *Nemocardium sp.* | Lamprell & Whitehead 1992; ter Poorten 2013. |
| *Neocallichirus angelikae* | Sakai 2000; Poore 2004. |
| *Neotrigonia sp.* | Tevesz 1975; Morton 1987; Lamprell & Whitehead 1992; Glavinic 2010. |
| Nephtyidae | Fauchald 1963; Rainer & Hutchings 1977; Beesley et al. 2000. |
| Nereididae | Beesley et al. 2000. |
| *Nerita atramentosa* | Underwood 1975; Gowlett-Holmes 2008; Edgar 2008; Przeslawski 2011. |
| *Notoacmea flammea* | Ponder & Creese 1980; Gowlett-Holmes 2008; Edgar 2008; https://www.sealifebase.ca/summary/Notoacmea-flammea.html |
| *Notospermus sp.* | Egan & Anderson 1979; Gowlett-Holmes 2008; Edgar 2008. |
| *Nototeredo edax* | McKoy 1980; Rayner 1983; Mann & Gallager 1985; MacIntosh et al. 2014. |
| Oenonidae | Beesley et al. 2000. |
| Oligochaeta | Parish 1981; Bonomi & Erseus 1984; Giere 2006; Edgar 2008; Pinder 2010; Lobo & de Gama 2011. |
| Onuphidae | Beesley et al. 2000. |
| Opheliidae | Beesley et al. 2000. |
| *Ophiactis tricolor* | McGovern 2002a; McGovern 2002b; Edgar 2008. |
| *Ophiomyxa australis* | Edgar 2008; Franklin & O'Hara 2008; Gowlett-Holmes 2008. |
| *Ophionereis sp.* | Selvakumaraswamy & Byrne 1995; Edgar 2008; Gowlett-Holmes 2008; Yokoyama & Amaral 2011. |
| *Ophiothrix caespitosa* | Selvakumaraswamy & Byrne 2000; Edgar 2008; Gowlett-Holmes 2008. |
| Ophiuroidea | Selvakumaraswamy & Byrne 1995; Selvakumaraswamy & Byrne 2000; Edgar 2008; Franklin & O'Hara 2008; Gowlett-Holmes 2008; Yokoyama & Amaral 2011. |
| Orbiniidae | Beesley et al. 2000. |
| Oweniidae | Beesley et al. 2000. |
| Paguridae | Hale 1927; Poore 2004; Wada et al. 2000; Mantelatto et al. 2007; Gowlett-Holmes 2008; Korn et al. 2018; Kornienko et al. 2019. |
| *Palaemon intermedius* | Hale 1927; Walker & Poore 2003; Poore 2004; Bilgin & Samsun 2006; Gowlett-Holmes 2008; Mortari et al. 2010. |
| *Paradentalium intercalatum* | Lamprell & Healy 1998. |
| *Paragrapsus gaimardii* | Hale 1927; Poore 2004; Gowlett-Holmes 2008. |
| Paraonidae | Beesley et al. 2000. |
| *Paraproto spinosa* | Hale 1927; Takeuchi & Hirano 1991; Guerra-Garcia & Takeuchi 2004. |
| Parapseudidae | Hale 1927; Blazwicz-Paszkowycz & Bamber 2007a; Blazwicz-Paszkowycz & Bamber 2007b; Ferreira et al. 2015; Rumbold et al. 2015; Crustacea.net (online resource). |
| *Parastacilla bakeri* | Hale 1927; King 2000; Crustacea.net (online resource). |
| Pardaliscidae | Hale, 1927; Barnard & Karaman 1991; Crustacea.net (online resource). |
| *Parvulastra exigua* | Edgar 2008; Gowlett-Holmes 2008; Roediger & Bolton 2008; Barbosa et al. 2012; Nguyen & Byrne 2014. |
| Patellidae | Woods 1876; Ridgway et al. 1998; Edgar 2008; Zegaoula et al. 2016; online resource. |
| Pectinariidae | Beesley et al. 2000; Hutchings et al. 2002. |
| *Peculator porphyria* | Bouchet & Kantor 2000; Bouchet & Kantor 2004; Harasewych & Kantor 2005; https://seashellsofnsw.org.au/Volutomitridae/Pages/volutomitridae_intro.htm; https://molluscsoftasmania.org.au/project/peculator-porphyria/ |
| *Penaeus latisulcatus* | Hale 1927; Gordon 1979; Penn 1980; Poore 2004; Hackett 2017. |
| Perthiidae | Hale 1927; Gowlett-Holmes 2008; Crustacea.net (online resource). |
| *Phasianotrochus eximius* | Edgar 2008; Gowlett-Holmes 2008; Bell et al. 2014; https://molluscsoftasmania.org.au/project/phasianotrochus-eximius/ |
| *Philine angasi* | Rudman 1972; Edgar 2008; Gowlett-Holmes 2008; Price et al. 2011; Nimbs & Smith 2016. |
| Phoratopodidae | Hale 1927; Bruce 1981. |
| Phoxocephalidae | Barnard & Drummond 1978; Slattery 1985; Barnard & Karaman 1991. |
| *Phyllodoce novaehollandiae* | Beesley et al. 2000. |
| Phyllodocidae | Beesley et al. 2000. |
| *Pilumnopeus serratifrons* | Hale 1927; Greenwood & Fielder 1984; Davie 2002b; Poore 2004; Gowlett-Holmes 2008. |
| *Pinna bicolor* | Butler 1987; Lamprell & Whitehead 1992; Gowlett-Holmes 2008; Idris et al. 2012. |
| *Placamen flindersi* | Lamprell & Whitehead 1992. |
| Plakarthriidae | Hale 1927; Wilson et al. 1976; Poore & Brandt 2001. |
| *Platynympha longicaudata* | Hale 1927; Bruce 2003; Crustacea.net (online resource). |
| Polynoidae | Beesley et al. 2000. |
| Polyplacophora | Edgar 2008; Gowlett-Holmes 2008; Sigwart et al. 2013. |
| *Portunus pelagicus* | Hale 1927; Poore 2004; Gowlett-Holmes 2008; Johnson et al. 2010. |
| *Propefusus australis* | Edgar 2008; Gowlett-Holmes 2008; https://www.sealifebase.ca/summary/Fusinus-australis.html |
| Pteriidae | Southgate & Lucas 2008; Milione & Southgate 2012. |
| *Pterochelus triformis* | Vokes 1993; Edgar 2008; Gowlett-Holmes 2008. |
| *Rathbunaria orientalis* | Davie 2002a; Poore 2004; NG 2010. |
| *Rhyssoplax sp.* | Edgar 2008; Gowlett-Holmes 2008; Sigwart et al. 2013. |
| *Rissoina crassa* | Ponder 1984; Gowlett-Holmes 2008. |
| *Rissoina fasciata* | Ponder 1984; Gowlett-Holmes 2008. |
| Sabellariidae | Beesley et al. 2000; Hutchings et al. 2012. |
| Sabellidae | Beesley et al. 2000. |
| *Sabia australis* | Morton & Jones 2000; https://seashellsofnsw.org.au/Hipponicidae/Pages/Hipponix_australis.htm |
| *Saccella crassa* | Hutchings & Haedrich 1984; Morton 2012. |
| *Salinator fragilis* | Golding et al. 2007; Golding et al. 2008; https://seashellsofnsw.org.au/Amphibolidae/Pages/Amphibolidae_intro.htm. |
| *Scissurella cyprina* | Geiger & Jansen 2004; https://seashellsofnsw.org.au/Scissurellidae/Pages/scissurellidae_intro.htm |
| Serpulidae | Dew 1959; Beesley et al. 2000. |
| Sigalionidae | Beesley et al. 2000. |
| *Simplisetia aequisetis* | Beesley et al. 2000. |
| Sipuncula | Beesley et al. 2000; Edgar 2008; Gowlett-Holmes 2008. |
| *Solemya australis* | Reid & Brand 1987; Pennec & Beninger 2000. |
| *Sphaerosyllis sp.* | Beesley et al. 2000. |
| Spionidae | Beesley et al. 2000. |
| *Spisula trigonella* | Murawski & Serchuk 1982; Cerrato & Keith 1992; Gaspar et al. 1995. |
| *Stenochiton cymodocealis* | Ashby 1923; Gowlett-Holmes 2008. |
| *Stenochiton pilsbryanus* | Ashby 1923; Gowlett-Holmes 2008. |
| *Stenochiton sp.* | Ashby 1923; Gowlett-Holmes 2008. |
| *Stimdromia lateralis* | Jamieson et al. 1993; Poore 2004; Gowlett-Holmes 2008. |
| Syllidae | Beesley et al. 2000. |
| *Synischia levidensis* | Hale 1927; Poore & Ton 1993. |
| Tanaidacea | Hale 1927; Blazwicz-Paszkowycz & Bamber 2007a; Blazwicz-Paszkowycz & Bamber 2007b; Ferreira et al. 2015; Rumbold et al. 2015; Crustacea.net (online resource). |
| *Tanea sagittata* | Kingsley-Smith et al. 2005; Richardson et al. 2005; Kulikova et al. 2007; Edgar 2008; Gowlett-Holmes 2008; Huelsken et al. 2008. |
| *Tasmanoplax latifrons* | Nye 1974; Simons & Jones 1981; Pasupathi & Kannupandi 1988a; Pasupathi & Kannupandi 1988b; Poore 2004. |
| *Tawera lagopus* | Nielsen 1963; Roberts 1984; Lamprell & Whitehead 1992; Bellchambers 1998; Edgar 2008; Dent et al. 2016; https://molluscsoftasmania.org.au/project/tawera-lagopus/ |
| *Tellinides margaritinus* | Lamprell & Whitehead 1992; http://portphillipmarinelife.net.au/species/5648; http://www.marlin.ac.uk/biotic/browse.php?sp=4354 |
| *Tellina sp.* | Lamprell & Whitehead 1992; http://portphillipmarinelife.net.au/species/5648; http://www.marlin.ac.uk/biotic/browse.php?sp=4354 |
| *Temnopleurus michaelseni* | Williamson & Steinberg 2002; Gowlett-Holmes 2008; https://molluscsoftasmania.org.au/project/tellinides-margaritinus/ |
| Terebellidae | Beesley et al. 2000. |
| Trapezidae | Lamprell & Whitehead 1992; Tan & Low 2013. |
| Trichobranchidae | Beesley et al. 2000; Hutchings & Peart 2000. |
| *Tucetona sp.* | Iredale 1929; Lamprell & Whitehead 1992; Crncevic et al. 2013. |
| Turritellidae | Marwick & Hutt 1956; Garrad 1982; Allmon 2011. |
| *Uniophora granifera* | Edgar 2008; Gowlett-Holmes 2008; O'Hara & Byrne 2017. |
| Veneridae | Nielsen 1963; Roberts 1984; Lamprell & Whitehead 1992; Bellchambers 1998; Edgar 2008; Dent et al. 2016; online resource. |
| *Venerupis anomala* | Lamprell & Whitehead 1992; Mohammad et al. 2014. |
| Vitrinellidae | Bartsch 1952; Pilsbry & Olsson 1952; Bieler & Mikkelsen 1988; Marshall 1988; Rolan & Sellanes 2004. |
| *Xenostrobus inconstans* | Gowlett-Holmes 2008; Colgan & da Costa 2013. |
| *Zeacumantus diemenensis* | Adachi & Wada 1999; Gowlett-Holmes 2008. |
| *Zeacumantus plumbeus* | Adachi & Wada 1999; Gowlett-Holmes 2008. |
|  |  |
| **Online resources** |  |
| Crustacea.net | http://www.crustacea.net/crustace/amphipoda/index.htm |
| BIOTIC | http://www.marlin.ac.uk/biotic/ |
| WoRMS | http://www.marinespecies.org |
| ATLAS of Living Australia | http://www.ala.org.au |
| Sealife Base | https://www.sealifebase.ca/ |
| Bug guide | https://www.mdfrc.org.au/bugguide/index.htm |

**Table S3.** Full reference list.

1. Ab Rahim, E. S., T. T. T. Nguyen, B. Ingram, C. Riginos, K. J. Weston, and C. D. H. Sherman. 2016. Species composition and hybridisation of mussel species (Bivalvia: Mytilidae) in Australia. Marine and Freshwater Research. 67: 1955–1963. <https://doi.org/10.1071/MF15307>
2. Adachi, N. and Wada, K. 1999. Distribution in relation to life history in the direct-developing gastropod *Batillaria cumingi* (Batillariidae) on two shores of contrasting substrata. J. Moll. Stud. 65: 275-287. <https://doi.org/10.1093/mollus/65.3.275>
3. Adrianov, A. V., and A. S. Maiorova. 2010. Reproduction and development of common species of peanut worms (Sipuncula) from the Sea of Japan. Russian Journal of Marine Biology 36:1-15. <https://doi.org/10.1134/S1063074010010013>
4. Ahn, I.Y. 1993. Enhanced particle flux through the biodeposition by the Antarctic suspension-feeding bivalve *Laternula elliptica* in Marian Cove, King George Island. J. Exp. Mar. Biol. Ecol. 171: 75-90. <https://doi.org/10.1016/0022-0981(93)90141-A>
5. Akiyama, T., and Yamamoto, M. 2004. Life history of *Nippoleucon hinumensis* (Crustacea: Cumacea: Leuconidae) in Seto Inland Sea of Japan. I. Summer diapause and molt cycle. Marine Ecology Progress Series. 284: 211-225. <https://doi.org/10.3354/meps284211>
6. Aktipis, S. W., E. Boehm, and G. Giribet. 2011. Another step towards understanding the slit-limpets (Fissurellidae, Fissurelloidea, Vetigastropoda, Gastropoda): a combined five-gene molecular phylogeny. Zoologica Scripta 40:238-259. <https://doi.org/10.1111/j.1463-6409.2010.00468.x>
7. Allmon, W. D. 2011. Natural History of Turritelline Gastropods (Cerithiodea: Turritellidae): A Status Report. Malacologia 54:159-202. <https://doi.org/10.4002/040.054.0107>
8. Angeloni, L., Bradbury, J.W., Burton, R.S., 2002. Multiple mating, paternity, and body size in a simultaneous hermaphrodite, *Aplysia California*. Behavioral Ecology. 4: 554-560. <https://doi.org/10.1093/beheco/arg033>
9. Ashby, E. 1923. Notes on the genus *Stenochiton* and the discovery and recognition of the type of Blainville’s *Chiton longicymba* in *Stenochiton juloides*, Adams and Angas. Proceedings of the Malacological Society. 15: 260-265.
10. Azman, B.A.R. 2009. Cyproideidae. Lowry, J.K. & Myers, A.A. (Eds) (2009) Benthic Amphipoda (Crustacea: Peracarida) of the Great Barrier Reef, Australia. Zootaxa. 2260: 1–930. <https://doi.org/10.11646/zootaxa.2260.1.1>
11. Baird, M., Lee, D.E., Lamare, M.D. 2013. Reproduction and growth of the Terebratulid Brachiopod *Liothyrella neozelanica* Thomson, 1918 from Doubtful Sound, New Zealand. Biol. Bull. 225: 125-136. <https://doi.org/10.1086/BBLv225n3p125>
12. Bakken, T. 2002. A new species of *Neanthes* (Polychaeta: Nereididae) from Southern Australia. Memoirs of Museum of Victoria. 59: 327-331. <https://doi.org/10.24199/j.mmv.2002.59.4>
13. Barbosa, S. S., O. Selma Klanten, H. Jones, and M. Byrne. 2012. Selfing in *Parvulastra exigua*: an asterinid sea star with benthic development. Marine Biology 159:1071-1077. <https://doi.org/10.1007/s00227-012-1887-8>
14. Barnard, J. L., and G. S. Karaman. 1991. The families and genera of marine gammaridean Amphipoda (except marine gammaroids). Part 2. Records of the Australian Museum, Supplement 13:419-866. <https://doi.org/10.3853/j.0812-7387.13.1991.367>
15. Barnard, J., and Drummond, M.M. 1978. Gammaridean Amphipoda of Australia, Part III: The Phoxocephalidae. Smithsonian Contributions to Zoology. Number 245, 1-551. <https://doi.org/10.5479/si.00810282.245>
16. Barnard, J.L. and Karaman, G.S. 1991. The families and genera of marine Gammaridean Amphipoda (except Marine Gammaroids). Records of the Australian Museum. 2: 419-866. <https://doi.org/10.3853/j.0812-7387.13.1991.367>
17. Bartsch, P. 1952. New mollusks of the Family Vitrinellidae from the West Coast of America. Proceedings U.S. National Museum. 32: 167-177. <https://doi.org/10.5479/si.00963801.32-1520.167>
18. Bayle, I.A.E. and Ellis, P. 1969. *Haloniscus searlei* Chilton: An aquatic ‘Terrestial’ isopod with remarkable powers of osmotic regulation. Comp. Biochem. Physiol. 31: 523-528. <https://doi.org/10.1016/0010-406X(69)90032-2>
19. Beaver, P. E., D. J. Bucher, and R. Joannes-Boyau. 2016. Growth patterns of three bivalve species targeted by the Ocean Cockle Fishery, southern New South Wales: *Eucrassatella kingicola* (Lamarck, 1805); *Glycymeris grayana* (Dunker, 1857); and *Callista (Notocallista) kingii* (Gray, 1827). Molluscan Research 37:104-112. <https://doi.org/10.1080/13235818.2016.1253430>
20. Beesley. P.L., Ross, G.J.B., Glasby, C.J. 2000. Polychaetes & Allies: The Southern Synthesis. Fauna of Australia 4A. Polychaeta, Myzostomida, Pogonophora, Echiura, Sipuncula. CSIRO Publ, Melbourne, 465 pp.
21. Bell, J. E., M. J. Bishop, R. B. Taylor, and J. E. Williamson. 2014. Facilitation cascade maintains a kelp community. Marine Ecology Progress Series 501:1-10. <https://doi.org/10.3354/meps10727>
22. Bellchambers, L.M. 1998. Ecology and Ecophysiology of *Katelysia scalarina* (Bivalvia: Veneridae), a commercially exploited clam. PhD Thesis. University of Tasmania.
23. Benítez-Villalobos, F., O. H. Avila-Poveda, and I. S. Gutiérrez-Méndez. 2013. Reproductive biology of *Holothuria fuscocinerea* (Echinodermata: Holothuroidea) from Oaxaca, Mexico. Sexuality and Early Development in Aquatic Organisms 1:13-24. <https://doi.org/10.3354/sedao00003>
24. Bieler, R., and Mikkelsen, P.M. 1988. Anatomy and reproductive biology of two western Atlantic species of Vitrinellidae, with a case of protandrous hermaphroditism in the Rissoacea. The Nautilus. 102: 1-29.
25. Bilgin, S., and Samsun, O. 2006. Fecundity and egg size of three shrimp species, *Crangon crangon, Palaemon adspersus*, and *Palaemon elegans* (Crustacea: Decapoda: Caridea), off Sinop Peninsula (Turkey) in the Black Sea. Turk J. Zool. 30: 413-421.
26. Black, J. H. 2013. Spawning and Development of *Bedeva paivae* (Crosse, 1864) (Gastropoda: Muricidae), Compiled from Notes and Observations by Florence V. Murray and G. Prestedge. Journal of the Malacological Society of Australia 3:215-221. <https://doi.org/10.1080/00852988.1976.10673897>
27. Blazewicz-Paszkowycz, M., and Bamber, R. 2007a. Parapseudid tanaidaceans (Crustacea: Tanaidacea: Apseudomorpha) from Eastern Australia. Zootaxa. 1401: 1-32. <https://doi.org/10.11646/zootaxa.1401.1.1>
28. Blazewicz-Paszkowycz, M., and Bamber, R. 2007b. New apseudomorph tanaidaceans (Crustacea: Peracarida: Tanaidacea) from eastern Australia: Apseudidae, Whiteleggiidae, Metapseudidae and Pagurapseudidae. Memoirs of Museum Victoria. 64: 107-148. <https://doi.org/10.24199/j.mmv.2007.64.11>
29. Blazewicz-Paszkowycz, M., R. Bamber, and G. Anderson. 2012. Diversity of Tanaidacea (Crustacea: Peracarida) in the world's oceans--how far have we come? PLoS One 7: e33068. <https://doi.org/10.1371/journal.pone.0033068>
30. Blinn, D.W., Blinn, S.L., Bayly, A.E. 1989. Feeding ecology of *Haloniscus searlei* Chilton, an Oniscoid Isopod living in Athalassic saline waters. Aust. J. Mar. Freshwater R. 40: 295-301. <https://doi.org/10.1071/MF9890295>
31. Boissin, E., Chenuil, A., Feral, J.-P. 2010. Species of the complex A*mphipholis squamata* (Ophiuroidae) from Marseilles. Echinoderms: Durham – Harris et al. (eds). Taylor & Francis Group, London, ISBN 978-0-415-40819-6. pp. 135-138.
32. Bonomi, G. and Erseus, C. 1984. Aquatic Oligochaeta. Proceedings of the Second International Symposium on Aquatic Oligochaete Biology, held in Pallanza, Italy, September 21-24. <https://doi.org/10.1007/978-94-009-6563-8>
33. Borojevic, R., Boury-Esnault, N., Vacelet, J. 2000. A revision of the supraspecific classification of the subclass Calcaronea (Porifera, class Calcarea). Zoosystema. 22: 203-263.
34. Bouchet, P., and Kantor, Y.I. 2000. A new species of *Volutomitra* (Gastropoda: Volutomitridae) from New Caledonia. Venus (Jap. Jour. Malac.). 59: 181-190.
35. Bouchet, P., and Y. I. Kantor. 2004. New Caledonia: The major centre of biodiversity for volutomitrid molluscs (Mollusca: Neogastropoda: Volutomitridae). Systematics and Biodiversity 1:467-502. <https://doi.org/10.1017/S1477200003001282>
36. Breitfuss, M.J., Connolly, R.M., Dale, P.E.R. 2004. Densities and aperture sizes of burrows constructed by *Helograpsus haswellianus* (Decapoda: Varunidae) in saltmarshes with and without mosquito-control runnels. Wetlands. 24: 14-22. [https://doi.org/10.1672/0277-5212(2004)024[0014:DAASOB]2.0.CO;2](https://doi.org/10.1672/0277-5212(2004)024%5b0014:DAASOB%5d2.0.CO;2)
37. Bruce, N. L. 1986. Cirolanidae (Crustacea: Isopoda) of Australia. Records of the Australian Museum. Supplement 6:1-239. <https://doi.org/10.3853/j.0812-7387.6.1986.98>
38. Bruce, N.L. 2003. New genera and species of sphaeromatid isopod crustaceans from Australian marine coastal waters. Memoirs of Museum Victoria. 60: 309-369. <https://doi.org/10.24199/j.mmv.2003.60.28>
39. Bruce. N.L. 1981. Redescription of the isopod (Crustacea) Family Phoratopodidae. Beaufortia. 31: 109-110.
40. Bulleri, F., M. G. Chapman, and A. J. Underwood. 2004. Patterns of movement of the limpet *Cellana tramoserica* on rocky shores and retaining seawalls. Marine Ecology Progress Series. 281:121-129. <https://doi.org/10.3354/meps281121>
41. Burden, D. K., C. M. Spillmann, R. K. Everett, D. E. Barlow, B. Orihuela, J. R. Deschamps, K. P. Fears, D. Rittschof, and K. J. Wahl. 2014. Growth and development of the barnacle *Amphibalanus amphitrite*: time and spatially resolved structure and chemistry of the base plate. Biofouling. 30:799-812. <https://doi.org/10.1080/08927014.2014.930736>
42. Burn, R. 2006. A checklist and bibliography of the Opisthobranchia (Mollusca: Gastropoda) of Victoria and the Bass Strait area, south-eastern Australia. Museum Victoria Science Reports. 10: 1-42. <https://doi.org/10.24199/j.mvsr.2006.10>
43. Butler, S.N., Reid, M., Bird, F.L. 2009. Population biology of the ghost shrimp, *Trypaea australiensis* and *Biffarius arenosus* (Decapoda: Thalassinidae), in Western Port, Victoria. Memoirs of Museum Victoria. 66: 43-59. <https://doi.org/10.24199/j.mmv.2009.66.6>
44. Butler, A.J. 1987. Ecology of *Pinna bicolor* Gmelin (Mollusca: Bivalvia) in Gulf St Vincent, South Australia: density, reproductive cycle, recruitment, growth and mortality at three sites. Aust. J. Mar. Freshwater. Res. 38: 743-769. <https://doi.org/10.1071/MF9870743>
45. Bynum, K.H. 1978. Reproductive biology of *Caprella penantis* Leach, 1814 (Amphipoda: Caprellidae) in North Carolina, U.S.A. Estuarine and Coastal Marine Science. 7: 473-485. <https://doi.org/10.1016/0302-3524(78)90124-X>
46. Cadien, D. and Brusca, C. 1993. Anthuridean isopods (Crustacea) of California and the temperate Northeast Pacific. SCAMIT Newslett 12: 1–26.
47. Cantin, Agnes. 2010. Population biology of two sympatric mud cockles, *Katelysia peronii* and *K. scalarina* (Bivalvia: Veneridae), with implications for their management. PhD thesis. Flinders University. Adelaide.
48. Cerrato, R.M. and Keith, D.L. 1992. Age structure, growth, and morphometric variations in the Atlantic surf clam, *Spisula solidissima*, from estuarine and inshore waters. Marine Biology, 114: 581-593. <https://doi.org/10.1007/BF00357255>
49. Chan, K., and B. Morton. 2005. The reproductive biology of *Nassarius festivus* (Powys, 1835) (Gastropoda: Nassariidae) in relation to seasonal changes in temperature and salinity in subtropical Hong Kong. Aquatic Ecology 39:213-228. <https://doi.org/10.1007/s10452-004-6144-x>
50. Cicero, J. M., M. M. Adair, R. C. Adair, W. B. Hunter, P. B. Avery, and R. F. Mizell. 2017. Predatory Behavior of Long-Legged Flies (Diptera: Dolichopodidae) and Their Potential Negative Effects on the Parasitoid Biological Control Agent of the Asian Citrus Psyllid (Hemiptera: Liviidae). Florida Entomologist 100:485-487. <https://doi.org/10.1653/024.100.0243>
51. Coffman, W., L. Ferrington Jr.. 1996. Chironomidae. I*n* R Merritt, K Cummins (eds). An Introduction to the Aquatic Insects of North America. Dubuque, Iowa, USA: Kendall/Hunt Publishing Company. Pp. 591-754.
52. Cohen, B. F., and G. C. B. Poore. 1994. Phylogeny and biogeography of the Gnathiidae (Crustacea: Isopoda) with descriptions of new genera and species, most from southeastern Australia. Memoirs of the Museum of Victoria 54:271-397. <https://doi.org/10.24199/j.mmv.1994.54.13>
53. Colgan, D. J., and P. da Costa. 2013. Invasive and non-invasive lineages in *Xenostrobus* (Bivalvia: Mytilidae). Molluscan Research 33:272-280. <https://doi.org/10.1080/13235818.2013.826574>
54. Croker, R.A. 1967. Diversity in five sympatric species of intertidal amphipods (Crustacea: Haustoriidae). Ecological Monographs. 37: 173-200. <https://doi.org/10.2307/1948437>
55. Davie, P. J. F. 2002a. Crustacea: Malacostraca: Phyllocarida, Hoplocarida, Eucarida (Part 1). In: A. Wells, & W. W. K. Houston, (Eds) *Zoological Catalogue of Australia.* CSIRO Publishing, Melbourne. Pp. 551.
56. Davie, P. J. F. 2002b. Crustacea: Malacostraca: Eucarida (Part 2). Decapoda – Anomura, Brachyura. In: A. Wells, & W. W. K. Houston, (Eds) *Zoological Catalogue of Australia.* CSIRO Publishing, Melbourne. Pp. 637.
57. De Paula, D. R., A. C. Almeida, and G. B. Jacobucci. 2016. Reproductive features of sympatric species of *Caprella* (Amphipoda) on the Southeastern Brazilian coast: a comparative study. Crustaceana 89:933-947. <https://doi.org/10.1163/15685403-00003566>
58. De Roach, J. 2006. The polychaetes *Australonereis ehlersi* (Augener) and *Simplisetia aequisetis* (Augener) within the eutrophic Swan River Estuary, Western Australia: Life history, population structure and effects on sedimentary microbial nitrogen cycling. Honours thesis. The University of Western Australia.
59. Dennell, R. 1932. The habits and feeding mechanism of the amphipod *Haustorius arenarius* Slabber. Journal of the Linnean Society of London, Zoology. 38: 363– 388. <https://doi.org/10.1111/j.1096-3642.1933.tb00066.x>
60. Dent, J., Mayfield, S., Carrol, J. 2016. Harvestable biomass of *Katelysia* spp. In the South Australian commercial mud cockle fishery. Report to PIRSA Fisheries and Aquaculture. South Australian Research and Development Institute (Aquatic Sciences), Adelaide. SARDI Publication Number F2014/000191-2.
61. Dew, B. 1959. Serpulidae (Polychaeta) from Australia. Records of the Australian Museum 25:19-56. <https://doi.org/10.3853/j.0067-1975.25.1959.654>
62. Dijkstra, H. H., and A. G. Beu. 2018. Living scallops of Australia and adjacent waters (Mollusca: Bivalvia: Pectinoidea: Propeamussiidae, Cyclochlamydidae and Pectinidae). Records of the Australian Museum 70:113-330. <https://doi.org/10.3853/j.2201-4349.70.2018.1670>
63. Dittmann, S., Cantin, A., Noble, W. & Pocklington J. 2006. Macrobenthic Survey 2004 in the Murray Mouth, Coorong and Lower Lakes Ramsar Site, with an evaluation of food availability for shorebirds and possible indicator functions of benthic species. Department for Environment and Heritage, Adelaide.
64. Dorsey, J.H. 1981. The ecology of *Australonereis ehlersi* (Augener, 1913) and *Ceratonereis erythraeensis* Fauvel, 1919 (Polychaeta, Nereidae) living offshore from the Werribee Sewage-treatment farm, Port Phillip Bay, Victoria, Australia. PhD Thesis. University of Melburne.
65. Dourado, E. D. R., R. L. Ferreira-Keppler, R. T. Martins, and M. M. Ronderos. 2017. Biting midges (Diptera: Ceratopogonidae) from an urban forest fragment in Central Amazon (Brazil): Effects of opening areas on abundance, richness, and composition. An Acad Bras Cienc 89:2757-2770. <https://doi.org/10.1590/0001-3765201720170370>
66. Drake, P., and Arias, A.M. 1995. Distribution and production of *Microdeutopus Gryllotalpa* (Amphipoda: Aoridae) in a shallow coastal lagoon in the bay of Cadiz, Spain. Journal if Crustacean Biology. 15: 454-465. <https://doi.org/10.2307/1548767>
67. Durkina, V. B., J. W. Chapman, and N. L. Demchenko. 2018. *Ampelisca eschrichtii* Kroyer, 1842 (Ampeliscidae) of the Sakhalin Shelf in the Okhotsk Sea starve in summer and feast in winter. PeerJ 6: e4841. <https://doi.org/10.7717/peerj.4841>
68. Edgar, G. J. 2008. Australian Marine Life: The Plants and Animals of Temperate Waters. Reed New Holland.
69. Egan, E.A. and Anderson, D.T. 1979. The Reproduction of the Entozoic Nemertean *Gononemertes australiensis* Gibson (Nemertea: Hoplonemertea: Monostylifera) – Gonads, Gametes, Embryonic development and Larval development. Aust. J. Mar. Freshwater. Res. 30: 661-81. <https://doi.org/10.1071/MF9790661>
70. El-Deeb, R. S., F. A. Abdel Razek, H. A. Omar, A. R. Khafage, and K. K. Abdul-Aziz. 2018. The gametogenic cycle and spawning of the mussel *Brachidontes pharaonis* (Fischer, 1876) (Bivalvia: Mytilidae) from Alexandria Coast, Egypt. The Egyptian Journal of Aquatic Research 44:353-359. <https://doi.org/10.1016/j.ejar.2018.10.002>
71. Ellis, P. and Williams, W.D. 1969. The biology of *Haloniscus searlei* Chilton, an Oniscoid Isopod living in Australian salt lakes. Aust. J. Mar. Freshwater R. 21: 51-69. <https://doi.org/10.1071/MF9700051>
72. El-Sayed, A.A.M., El-Mekawy, H.A., Al-Hammady, M.A., Owen, N.A., Nassef, A.M. 2018. Aspects of reproductive biology of the mussel, *Brachidontes pharaonic* (Fisher, 1876) (Mytilidae: Bivalvia: Mollusca) From the Northestern coast of Suez Gulf, Egypt. Al Azhar Bulletin of Science. 29: 11-24. <https://doi.org/10.21608/absb.2018.33815>
73. Fauchald, K. 1965. Some Nephtyidae (Polychaeta) from Australian waters. Records of the Australian Museum 26: 333-339. <https://doi.org/10.3853/j.0067-1975.26.1965.682>
74. Fenton, G.E. 1986. Ecology and taxonomy of Mysids (Mysidacea: Crustacea). PhD thesis. University of Tasmania.
75. Ferreira, A. C., E. S. Ambrosio, and A. Rodrigues. 2015. Population ecology of *Sinelobus stanfordi* (Crustacea: Tanaidacea) in a temperate southern microtidal estuary. New Zealand Journal of Marine and Freshwater Research 49:462-471. <https://doi.org/10.1080/00288330.2015.1089914>
76. Fleming, C. A. 1977. The bivalve mollusc genus *Limatula*: A list of described species and a review of living and fossil species in the Southwest Pacific. Journal of the Royal Society of New Zealand 8:17-91. <https://doi.org/10.1080/03036758.1978.10419418>
77. Fletcher, W.J. 1984. Intraspecific variation in the population dynamics and growth of the limpet, *Cellana tramoserica*. Oecologia. 63: 110-121. <https://doi.org/10.1007/BF00379792>
78. Foote, B. 1987. Chironomidae (Chironomoidea). *In* F Stehr, ed. Immature Insects, Vol. 2. Dubuque, Iowa, USA: Kendall/Hunt Publishing Company. Pp. 762-764.
79. Franklin, A.M. and O'Hara, T.D. 2008. A new species in the genus *Ophiomyxa* from South-west Australian waters (Echinodermata: Ophiuroidea: Ophiomyxidae). Memoirs of Museum Victoria 65: 57–62. <https://doi.org/10.24199/j.mmv.2008.65.5>
80. Gan, H. M., M. H. Tan, B. T. Thai, and C. M. Austin. 2016. The complete mitogenome of the marine bivalve *Lutraria rhynchaena* Jonas 1844 (Heterodonta: Bivalvia: Mactridae). Mitochondrial DNA A DNA Mapp Seq Anal 27:335-336. <https://doi.org/10.3109/19401736.2014.892104>
81. Garrard, T. A. 1982. A supplement to Australian Recent Turritellidae (Gastropoda: Mollusca). Journal of the Malacological Society of Australia 5:195-200. <https://doi.org/10.1080/00852988.1982.10673950>
82. Gaspar, M.B., Castro, M., Monteiro, C.C. 1995. Age and growth rate of the clam, *Spisula solida* L., from a site off Vilamoura, south Portugal, determined from acetate replicas of shell sections. Scientia Marina. 59: 87-93.
83. Geiger, D. L., and P. Jansen. 2004. New species of Australian Scissurellidae (Mollusca: Gastropoda: Vetigastropoda) with remarks on Australian and Indo-Malayan species. Zootaxa 714: 1-72. <https://doi.org/10.11646/zootaxa.714.1.1>
84. Gerken, S. 2000. The Gynodiastylidae (Crustacea: Cumacea). Memoirs of the Museum of Victoria 59: 1-276. <https://doi.org/10.24199/j.mmv.2001.59.1>
85. Gerken, S. 2013. New Zealand Bodotriidae (Crustacea: Cumacea). Zootaxa 3630: 1-38. <https://doi.org/10.11646/zootaxa.3630.1.1>
86. Gerken, S. 2014. Eleven new species and a new genus of Diastylidae (Crustacea: Cumacea) from Australia and one new species from Canada. Records of the Australian Museum 66: 1-62. <https://doi.org/10.3853/j.2201-4349.66.2014.1601>
87. Giere, O. 2006. Ecology and Biology of Marine Oligochaeta – an Inventory rather than another Review. Hydrobiologia 564:103-116. <https://doi.org/10.1007/s10750-005-1712-1>
88. Glavinic, A. 2010. Systematics, Phylogeny, Phylogeography and Reproduction of Neotrigonia (Bivalvia: Palaeoheterodonta). PhD thesis. Flinders University.
89. Glover, E.A. and Taylor, J.D. 2001. Systematic revision of Australian and Indo-Pacific Lucinidae (Mollusca: Bivalvia): *Pillucina, Walucina* and descriptions of two new genera and four new species. Records of the Australian Museum. 53: 263-292. <https://doi.org/10.3853/j.0067-1975.53.2001.1349>
90. Glynn, P.W. 1970. On the Ecology of the Caribbean Chitons *Acanthopleura granulate* Gmelin and *Chiton tuberculatus*  Linne: Density, Mortality, Feeding, Reproduction, and Growth. Smithsonian Contributions to Zoology. Number 66. Washington. <https://doi.org/10.5479/si.00810282.66>
91. Golding, R.E., Byrne, M., Ponder, W.F. 2008. Novel copulatory structures and reproductive functions in Amphiboloidea (Gastropoda: Heterobranchia: Pulmonata). Invertebrate Biology. 127: 168-180. <https://doi.org/10.1111/j.1744-7410.2007.00120.x>
92. Golding, R.E., Ponder, W.F., Byrne, M. 2007. Taxonomy and anatomy of Amphiboloidea (Gastropoda: Heterobranchia: Archaeopulmonata). Zootaxa 1476: 1-50. <https://doi.org/10.11646/zootaxa.1476.1.1>
93. Gowlett-Holmes, K. 2008. A Field Guide to the Marine Invertebrates of South Australia. Notomares, Sandy Bay, Tasmania, Australia, 333 pp.
94. Grayson, J.E. and Chapman, M.G. 2004. Patterns of distribution and abundance of chitons of the genus *Ischnochiton* in intertidal boulder field. Austral Ecology. 29: 363-373. <https://doi.org/10.1111/j.1442-9993.2004.01375.x>
95. Greenwood, J. G., and D. R. Fielder. 1984. The zoeal stages of *Pilumnopeus serratifrons* (Kinahan, 1856) (Brachyura: Xanthidae) reared under laboratory conditions. Journal of Natural History 18: 31-40. <https://doi.org/10.1080/00222938400770051>
96. Griffin, D. J. G., and J. C. Yaldwin. 1971. Port Phillip Bay Survey 2. Brachyura (Crustacea, Decapoda). Memoirs of the National Museum of Victoria 32: 43-63. <https://doi.org/10.24199/j.mmv.1971.32.05>
97. Griffin, D.J.G. 1969. Breeding and Moulting cycles of two Tasmanian Grapsid crabs (Decapoda, Brachyura). Crustaceana. 16: 88-94. <https://doi.org/10.1163/156854068X00223>
98. Griffin, D.J.G. 1969. The ecological distribution of Grapsid and Ocypodid shore crabs (Decapoda, Brachyura) in Tasmania. Journal of Animal Ecology. 40: 597-621. <https://doi.org/10.2307/3440>
99. Guerra-García, J. M., and I. Takeuchi. 2004. The Caprellidea (Crustacea: Amphipoda) from Tasmania. Journal of Natural History 38: 967-1044. <https://doi.org/10.1080/0022293021000054497>
100. Hackett, N.E. 2017. Reproductive biology of the western king prawn *Penaeus (Melicertus) lastisulcatus* (Kishinouye 1896) in Spencer Gulf and Gulf St Vincent, South Australia. PhD thesis. Flinders University.
101. Hale, H. M. 1927. The Crustaceans of South Australia. Handbooks of the Flora and Fauna of South Australia Ft. II., pp. 201-380. Adelaide.
102. Harasewych, M.G. and Kantor, Y.I. 2005. *Daffymitra lindae*, a new genus and species of Volutomitridae (Neogastropoda) from the Bellingshausen Abyssal Plain. The Nautilus. 119: 149-152.
103. Harrison, K., and G. C. B. Poore. 1984. *Serolis* (Crustacea, Isopoda, Serolidae) from Australia, with a new species from Victoria. Memoirs of the Museum of Victoria. 45: 13-31. <https://doi.org/10.24199/j.mmv.1984.45.03>
104. Healy, J. M., P. M. Mikkelsen, and R. Bieler. 2015. Spermatogenic ultrastructure in the anomalodesmatan bivalve *Myochama anomioides* (Mollusca: Myochamidae) - does the nucleus help position the ‘temporary’ acrosome? Acta Zoologica 96: 487-496. <https://doi.org/10.1111/azo.12093>
105. Healy, J., and K. Lamprell. 1992. New species of Veneridae, Cardiidae, Crassatellidae, Tellinidae and Mactridae from Australia (Veneroida, Bivalvia, Mollusca). Journal of the Malacological Society of Australia 13: 75-97. <https://doi.org/10.1080/00852988.1992.10674037>
106. Herbert, D. G. 2012. A Revision of the Chilodontidae (Gastropoda: Vetigastropoda: Seguenzioidea) of Southern Africa and the South-Western Indian Ocean. African Invertebrates 53: 381-502. <https://doi.org/10.5733/afin.053.0209>
107. Herrmann, M., J. E. F. Alfaya, M. L. Lepore, P. E. Penchaszadeh, and J. Laudien. 2009. Reproductive cycle and gonad development of the Northern Argentinean *Mesodesma mactroides* (Bivalvia: Mesodesmatidae). Helgoland Marine Research 63: 207-218. <https://doi.org/10.1007/s10152-009-0150-2>
108. Hessler, R. R., and J.-O. Strömberg. 1989. Behavior of janiroidean isopods (Asellota), with special reference to deep-sea genera. Sarsia 74: 145-159. <https://doi.org/10.1080/00364827.1989.10413424>
109. Highsmith, R.C. and Coyle, K.O. 1991. Amphipod life histories: community structure, impact of temperature on decoupled growth and maturation rates, productivity, and P:B ratios. Amer. Zool. 31: 861-873. <https://doi.org/10.1093/icb/31.6.861>
110. Hooker, S.H., and Creese, R.G. 1995a. The reproductive biology of Pipi, *Paphies australis* (Gmelin, 1790) (Bivalvia: Mesodesmatidae). I. Temporal patterns of the reproductive cycle. Journal of Selfish Research. 14: 7-15.
111. Hooker, S.H., and Creese, R.G. 1995b. The reproductive biology of Pipi, *Paphies australis* (Gmelin, 1790) (Bivalvia: Mesodesmatidae). II. Temporal patterns of the reproductive cycle. Journal of Selfish Research. 14: 17-24.
112. Huang, C. W., and Y. C. Lee. 2016. Checklist of the family Epitoniidae (Mollusca: Gastropoda) in Taiwan with description of a new species and some new records. Biodivers Data J: e5653. <https://doi.org/10.3897/BDJ.4.e5653>
113. Huelsken, T., Marek, C., Schreiber, S., Schmidt, I., Holl-Mann, M. 2008. The Naticidae (Mollusca: Gastropoda) of Giglio Island (Tuscany, Italy): Shell characters, live animals, and molecular analysis of egg masses. Zootaxa. 1770: 1-40. <https://doi.org/10.11646/zootaxa.1770.1.1>
114. Hughes, L. E. 2017. Review of *Xenocheira* Haswell, 1879 (Crustacea: Amphipoda: Aoridae). Records of the Australian Museum 69: 223-236. <https://doi.org/10.3853/j.2201-4349.69.2017.1664>
115. Hughes, L.E. and Peart, R.A. 2013. New species and new records of Ampithoidae (Peracarida: Amphipoda) from Australian Waters. Zootaxa. 3719: 001-102. <https://doi.org/10.11646/zootaxa.3719.1.1>
116. Hutchings, J. A., and R. L. Haedrich. 1984. Growth and population structure in two species of bivalves (Nuculanidae) from the deep sea. Marine Ecology Progress Series 17: 135-142. <https://doi.org/10.3354/meps017135>
117. Hutchings, P. A. T., M. Capa, and R. Peart. 2012. Revision of the Australian Sabellariidae (Polychaeta) and description of eight new species. Zootaxa 3306: 1-60. <https://doi.org/10.11646/zootaxa.3306.1.1>
118. Hutchings, P. and Peart, R. 2000. A revision of the Australian Trichobranchidae (Polychaeta). Invertebrate Systematics. 14: 225-272. <https://doi.org/10.1071/IT98005>
119. Hutchings, P., and A. Murray. 1984. Taxonomy of polychaetes from the Hawkesbury River and the southern estuaries of New South Wales, Australia. Records of the Australian Museum, Supplement 3:1-118. <https://doi.org/10.3853/j.0812-7387.3.1984.101>
120. Idris, M. H., A. Arshad, S. M. N. Amin, S. B. Japar, S. K. Daud, A. G. Mazlan, M. S. Zakaria, and F. M. Yusoff. 2012. Age, growth and length-weight relationships of *Pinna bicolor* Gmelin (Bivalvia: Pinnidae) in the seagrass beds of Sungai Pulai Estuary, Johor, Peninsular Malaysia. Journal of Applied Ichthyology 28: 597-600. <https://doi.org/10.1111/j.1439-0426.2011.01807.x>
121. Iredale, T. 1929. Mollusca from the Continental Shelf of eastern Australia. No. 2. Records of the Australian Museum 17: 157-189. <https://doi.org/10.3853/j.0067-1975.17.1929.759>
122. Jamieson, B.G.M., Tudge, C.C., Scheltinga, D.M. 1993. The ultrastructure of the spermatozoon of *Dromidiopsis edwardsi* Rathbun, 1919 (Crustacea: Brachyura: Dromiidae): confirmation of a Dromiid sperm type. Aust. J. Zool. 41: 537-48. <https://doi.org/10.1071/ZO9930537>
123. Jansen, P. 1993. The family Trochidae (Mollusca: Gastropoda) in the Sydney metropolitan area and adjacent coast. Australian Zoologist. 29: 1-13. <https://doi.org/10.7882/AZ.1993.004>
124. Jeffery, C.J. 1997. The ecology of the rocky shore intertidal barnacle *Chamaesipho tasmanica* in New South Wales. PhD Thesis. The University of Sydney.
125. Jeffery, C.J. and Underwood, A.J. 2001. Longevity determines sizes of an adult intertidal barnacle. Journal of Experimental Marine Biology and Ecology. 256: 85-97. <https://doi.org/10.1016/S0022-0981(00)00307-5>
126. Jespersen, A., and Lützen, J. 2009. Structure of sperm. Spermatozeugmata and ‘lateral organs’ in the bivalve *Arthritica* (Galeommatoidea: Leptonidae). Acta Zoologica. 90: 51-67. <https://doi.org/10.1111/j.1463-6395.2008.00332.x>
127. Jocque, M., and W. Blom. 2009. Mysidae (Mysida) of New Zealand; a checklist, identification key to species and an overview of material in New Zealand collections. Zootaxa 2304: 1-20. <https://doi.org/10.11646/zootaxa.2304.1.1>
128. Johnson, D. D., C. A. Gray, and W. G. Macbeth. 2010. Reproductive Biology of *Portunus pelagicus* in a South-East Australian Estuary. Journal of Crustacean Biology 30: 200-205. <https://doi.org/10.1651/08-3076.1>
129. Jones, D. S. 2012. Australian barnacles (Cirripedia: Thoracica), distributions and biogeographical affinities. Integr Comp Biol 52: 366-387. <https://doi.org/10.1093/icb/ics100>
130. Kabat, A.R., and Hershler, R. 1993. The Prosobranch snail family Hydrobiidae (Gastropoda: Rissooidea): Review of classification and supraspecific taxa. Smithsonian contributions to zoology. Number 547. <https://doi.org/10.5479/si.00810282.547>
131. Kamihira, Y. 1981. Life history of sand-burrowing amphipod H*austorioides japonicus* (Crustacea: Dogielinotidae). Bull. Fac. Fish. Hokkaido. Univ. 32: 338-348.
132. Kang, D.-H., I.-Y. Ahn, and K.-S. Choi. 2009. The annual reproductive pattern of the Antarctic clam, *Laternula elliptica* from Marian Cove, King George Island. Polar Biology 32: 517-528. <https://doi.org/10.1007/s00300-008-0544-7>
133. Katrak G, Dittmann S, Seuront L. 2008. Spatial variation in burrow morphology of the mud shore crab, *Helograpsus haswellianus* (Brachyura, Grapsidae), in South Australian saltmarshes. Mar. Freshw. Res. 59: 902–911. <https://doi.org/10.1071/MF08044>
134. Katrak, G., and S. Dittmann. 2011. Site specific distribution of the mud shore crab *Helograpsus haswellianus* in temperate wetlands. Wetlands Ecology and Management 19: 433-448. <https://doi.org/10.1007/s11273-011-9227-1>
135. Keable, S.J. 2006. Taxonomic revision of *Natatolana* (Crustacea: Isopoda: Cirolanidae). Records of the Australian Museum. 58: 133-244. <https://doi.org/10.3853/j.0067-1975.58.2006.1469>
136. Kilburn, R.N. 1985. The family Epitoniidae (Mollusca: Gastropoda) in southern Africa and Mozambique. Ann. Natal. Mus. 27: 239-337.
137. Kilgallen, N. M., and J. K. Lowry. 2013. The lysianassid genus Pseudambasia in Australian waters (Crustacea, Amphipoda, Lysianassidae, Lysianassinae). Zootaxa 3710: 301-321. <https://doi.org/10.11646/zootaxa.3710.4.1>
138. King, R. 2009. Ampeliscidae. In: Lowry, J.K. & Myers, A.A. (Eds) (2009) Benthic Amphipoda (Crustacea: Peracarida) of the Great Barrier Reef, Australia. Zootaxa, 2260: 1–930. <https://doi.org/10.11646/zootaxa.2260.1.2>
139. King, R.A. 2003. *Neastacilla* Tattersall, 1921 redefined, with eight new species from Australia (Crustacea: Isopoda: Arcturidae). Memoirs of Museum Victoria. 60: 371-416. <https://doi.org/10.24199/j.mmv.2003.60.29>
140. King, R.A., 2000. Rediagnosis of the endemic southern Australian genus *Parastacilla* Hale, 1924 (Crustacea: Isopoda: Arcturidae) with descriptions of two new species. Memoirs of Museum Victoria 58: 125–136. <https://doi.org/10.24199/j.mmv.2000.58.6>
141. Kingsley-Smith, P. R., C. A. Richardson, and R. Seed. 2005. Growth and development of the veliger larvae and juveniles of *Polinices pulchellus* (Gastropoda: Naticidae). Journal of the Marine Biological Association of the United Kingdom 85: 171-174. <https://doi.org/10.1017/S0025315405011008h>
142. Korn, O. M., E. S. Kornienko, and N. I. Selin. 2018. Population biology and reproductive characteristics of the hermit crab *Pagurus minutus* Hess, 1865 (Decapoda: Anomura: Paguridae) in the northern part of the species range (Peter the Great Bay, the Sea of Japan). Marine Biology Research 14: 846-855. <https://doi.org/10.1080/17451000.2018.1503685>
143. Kornienko, E. S., N. I. Selin, and O. M. Korn. 2019. Population and reproductive characteristics of the hermit crab *Pagurus proximus* Komai, 2000 (Decapoda: Anomura: Paguridae) in the northern part of the species range. Journal of the Marine Biological Association of the United Kingdom 99: 101-109. <https://doi.org/10.1017/S0025315417001679>
144. Kulikova, V. A., K. G. Kolbin, and N. K. Kolotukhina. 2007. Reproduction and larval development of the gastropod *Cryptonatica janthostoma* (Gastropoda: Naticidae). Russian Journal of Marine Biology 33: 324-328. <https://doi.org/10.1134/S1063074007050094>
145. Lam-Gordillo, O., R. Baring, and S. Dittmann. 2019. Rediscovering the tortoise-crab, *Cryptocnemus vincentianus* Hale, 1927 (Brachyura: Leucosiidae). Transactions of the Royal Society of South Australia 143: 235-243. <https://doi.org/10.1080/03721426.2019.1655935>
146. Lamprell, K. and Whitehead, T. 1992. Bivalves of Australia, Vol. 1, Crawford House Press, Bathurst, NSW. pp. 182.
147. Lamprell, K.L. and Healy, J.M. 1998. A revision of the Scaphopoda from Australian waters. Records of the Australian Museum. 24: 1-189. <https://doi.org/10.3853/j.0812-7387.24.1998.1267>
148. LaSalle, M.W. and Bishop, D.T. 1990. Food habits of two larval files (Dolichopodidae: Diptera) in two Gulf coast oligohaline tidal marshes. Estuaries. 13: 341-348. <https://doi.org/10.2307/1351926>
149. Lee, C. H., B. K. Kaang, and Y. D. Lee. 2014. Spawning Behavior and Egg Development of *Aplysia kurodai* Inhabiting the Coastal Waters of Jeju Island, Korea. Dev Reprod. 18: 25-31. <https://doi.org/10.12717/DR.2014.18.1.025>
150. Leon-Cisneros, K., Mazariegos-Villareal, A., Miranda-Saucedo, C.M., Argumedo-Hernandez, U., Siqueiros-Beltrones, D., Serviere-Zaragoza, E. 2017. Diet of the volcano keyhle limpet *Fissurella volcano* (Gastropoda: Fissurellidae) in the subtropical rocky reefs of the Baja California Peninsula. Pacific Science. 71: 57-66. <https://doi.org/10.2984/71.1.5>
151. Lill, A.W., Lal, A., Closs, G.P. 2010. Life history and reproduction of two abundant mysids (Mysidacea: Mysidae) in an intermittently open New Zealand estuary. Marine and Freshwater Research. 61: 633-641. <https://doi.org/10.1071/MF09085>
152. Linse, K., J. A. Jackson, M. V. Malyutina, and A. Brandt. 2014. Shallow-water northern hemisphere *Jaera* (Crustacea, Isopoda, Janiridae) found on whale bones in the Southern Ocean deep sea: ecology and description of *Jaera tyleri* sp. nov. PLoS One 9: e93018. <https://doi.org/10.1371/journal.pone.0093018>
153. Liversage, K., and K. Benkendorff. 2017. The first observations of Ischnochiton (Mollusca, Polyplacophora) movement behaviour, with comparison between habitats differing in complexity. PeerJ 5: e4180. <https://doi.org/10.7717/peerj.4180>
154. Lobo, H., and R. d. G. Alves. 2011. Reproductive cycle of *Branchiura sowerbyi* (Oligochaeta: Naididae: Tubificinae) cultivated under laboratory conditions. Zoologia (Curitiba) 28:427-431. <https://doi.org/10.1590/S1984-46702011000400003>
155. Lord, J. P. 2011. Larval development, metamorphosis and early growth of the gumboot chiton *Cryptochiton stelleri* (Middendorff, 1847) (Polyplacophora: Mopaliidae) on the Oregon coast. Journal of Molluscan Studies 77: 182-188. <https://doi.org/10.1093/mollus/eyr004>
156. Lowry J.K. & Poore, G.C.B. (1985) The Ampeliscid Amphipods of South-eastern Australia (Crustacea). Records of the Australian Museum. 36: 259–298. <https://doi.org/10.3853/j.0067-1975.36.1985.348>
157. Lowry, J.K. and Azman, B.A.R. 2008. A new genus and species of cyproideid amphipod associated with unstalked crinoids on the Great Barrier Reef, Australia. Zootaxa. 1760: 59-68. <https://doi.org/10.11646/zootaxa.1760.1.5>
158. Lowry, J.K. and Stoddart, H.E. 1995. The Amphipoda (Crustacea) of Madang lagoon: Lysianassidae, Opisidae, Uristidae, Wandinidae and Stegocephalidae. Records of the Australian Museum. <https://doi.org/10.3853/j.0812-7387.22.1995.122>
159. Lowry, J.K. and Stoddart, H.E. 2002. The Amaryllididae of Australia (Crustacea: Amphipoda: Lysianassoidea). Records of the Australian Museum. 54: 129-214. <https://doi.org/10.3853/j.0067-1975.54.2002.1363>
160. Lucas, J.S. 1972. The larval stages of some Australian species of *Halicarcinus* (Crustacea, Brachyura, Hymenosomatidae). Bulletin of Marine Science. 22: 1-17.
161. MacIntosh, H., R. de Nys, and S. Whalan. 2014. Contrasting life histories in shipworms: Growth, reproductive development and fecundity. Journal of Experimental Marine Biology and Ecology 459: 80-86. <https://doi.org/10.1016/j.jembe.2014.05.015>
162. Malaquias, M. A. E., E. Berecibar, and D. G. Reid. 2009. Reassessment of the trophic position of Bullidae (Gastropoda: Cephalaspidea) and the importance of diet in the evolution of cephalaspidean gastropods. Journal of Zoology 277: 88-97. <https://doi.org/10.1111/j.1469-7998.2008.00516.x>
163. Mann, R. and Gallager, S.M. Growth, Morphometry and biochemical composition of the wood boring molluscs *Teredo navalis* L., *Bankia gouldi* (Bartsch), and *Nototeredo knoxi* (Bartsch) (Bivalvia: Teredinidae). J. Exp. Mar. Biol. Ecol. 85: 229-251. <https://doi.org/10.1016/0022-0981(85)90160-1>
164. Mantelatto, F.L., Faria, F.C.R., Iossi, C.L., Biagi, R. 2007. Population and reproductive features of the western Atlantic hermit crab *Pagurus criniticornis* (Anomura, Paguridae) from Anchieta Island, southeastern Brazil. Iheringia, Ser. Zool. Porto Alegre. 97: 314-320. <https://doi.org/10.1590/S0073-47212007000300016>
165. Marshall, B. A. 1988. Skeneidae, Vitrinellidae and Orbitestellidae (Mollusca: Gastropoda) associated with biogenic substrata from bathyal depths off New Zealand and New South Wales. Journal of Natural History 22: 949-1004. <https://doi.org/10.1080/00222938800770631>
166. Marwick, J., and Hutt, L. 1956. Generic revision of the Turritellidae. Proceedings of the Malacological Society. 32: 144-166.
167. Matthews, T.G. and Fairweather, P.G. 2003. Grow rates of the infaunal bivalve *Soletellina alba* (Lamarck, 1818) (Bivalvia: Psammobiidae) in an intermittent estuary of southern Australia. Estuarine Coastal and Shelf Science. 58: 873-885. <https://doi.org/10.1016/j.ecss.2003.07.003>
168. Matthews, T.G. and Fairweather, P.G. 2004. Effect of lowered salinity on the survival, condition and reburial of *Soletellina alba* (Lamarck, 1818) (Bivalvia: Psammobiidae). Austral Ecology. 29: 250-257. <https://doi.org/10.1111/j.1442-9993.2004.01345.x>
169. McGovern, T.M. 2002a. Patterns of sexual and asexual reproduction in the brittle star *Ophiactis savignyi* in the Florida Keys. Marine Ecology Progress Series. 230: 119-126. <https://doi.org/10.3354/meps230119>
170. McGovern, T.M. 2002b. Sex-ratio bias and clonal reproduction in the brittle star *Ophiactis savignyi*. Evolution. 56: 511-517. <https://doi.org/10.1111/j.0014-3820.2002.tb01362.x>
171. Mckillup S.C. and Butler A.J. 1979. Modification of egg production and packaging in response to food availability by *Nassarius pauperatus.* Oecologia 43: 221–231. <https://doi.org/10.1007/BF00344772>
172. McKoy, J. L. 1980. Distribution of shipworms (Bivalvia: Teredinidae) in the New Zealand region. New Zealand Journal of Marine and Freshwater Research 14: 263-275. <https://doi.org/10.1080/00288330.1980.9515869>
173. Middelfart, P. 2002. A revision of the Australian Condylocardiinae (Bivalvia: Carditoidea: Condylocardiidae). Molluscan Research. 22: 23-85.
174. Milione, M., and P. C. Southgate. 2012. Reproductive cycle of the winged pearl oyster, *Pteria penguin* (Röding 1793) (Pteriidae) in north-eastern Australia. Invertebrate Reproduction & Development 56: 164-171. <https://doi.org/10.1080/07924259.2011.583693>
175. Miller, B.A. 1975. The biology of *Terebra gouldi* Deshayes, 1859, and a discussion of life history similarities among other Terebrids of similar proboscis type. Pacific Science. 29: 227-241.
176. Mohammad, S.H., Belal, A.A.M., Hassan, S.S.Z. 2014. Growth, age and reproduction of the commercially clams *Venerupis aurea* and *Ruditapes decussatus* in Timsah Lake, Suez Canal, Egypt. Indian Journal of Geo-Marine Sciences. 43: 598-600.
177. Mortari, R. C., B. G. Nunes Pralon, and M. L. Negreiros-Fransozo. 2009. Reproductive biology of *Palaemon pandaliformis* (Stimpson, 1871) (Crustacea, Decapoda, Caridea) from two estuaries in southeastern Brazil. Invertebrate Reproduction & Development 53: 223-232. <https://doi.org/10.1080/07924259.2009.9652308>
178. Morton, B. 1987. The functional morphology of *Neotrigonia margaritacea* (Bivalvia: Trigoniacea), with a discussion of phylogenetic affinities. Records of the Australian Museum 39: 339-354. <https://doi.org/10.3853/j.0067-1975.39.1987.173>
179. Morton, B. 2000. The biology and functional morphology of *Nucula pusilla* (Bivalvia: Protobranchia: Nuculidae) from Western Australia, Australia: primitive or miniature simplicity? Records of the Western Australian Museum. 27: 85-100. <https://doi.org/10.18195/issn.0312-3162.27(2).2012.085-100>
180. Morton, B. and Jones, D.S. 2000. The biology of *Hipponix australis* (Gastropoda: Hipponicidae) on *Nassarius pauperatus* (Nassaridae) in Princess Royal Harbour, Western Australia. J. Moll. Stud. 67: 247-255. <https://doi.org/10.1093/mollus/67.3.247>
181. Mullen, G.R., and Hribar, L.J. 1988. Biology and feeding behaviour of ceratopogonid larvae (diptera: Ceratopogonidae) in North America. Bull. Soc. Vector Ecol. 13: 60-81.
182. Murawski, S. and Serchuk, F.M. 1982. Assessments and current status of offshore surf clam, *Spisula solidissima*, populations off Middle Atlantic coast of the United States. U.S. Dep. Comm., Nat. Mar. Fish. Serv., Woods Hole Lab. 82-43, 59 pp.
183. Murina, G.-V.V. 1984. Ecology of Sipuncula. Marine Ecology Progress Series. 17: 1-7. <https://doi.org/10.3354/meps017001>
184. Murray, F.V. 1969. The spawn and early life history of *Cacozeliana granaria* (Kiener 1842) (Gastropoda: Cerithiidae). Memoirs of the National Museum of Victoria. 29: 111-114. <https://doi.org/10.24199/j.mmv.1969.29.09>
185. Myers, A. 2009. Corophiidae. In: Lowry, J.K. & Myers, A.A. (Eds) (2009) Benthic Amphipoda (Crustacea: Peracarida) of the Great Barrier Reef, Australia. Zootaxa, 2260: 1–930. <https://doi.org/10.11646/zootaxa.2260.1.2>
186. Nakano, T., and T. Ozawa. 2005. Systematic Revision of *Patelloida pygmaea* (Dunker, 1860) (Gastropoda: Lottiidae), with a Description of a New Species. Journal of Molluscan Studies 71: 357-370. <https://doi.org/10.1093/mollus/eyi039>
187. Ng, Peter K. L., 2010, On the Planopilumnidae Serène, 1984 (Crustacea: Brachyura: Pseudozioidea), with diagnoses of two new pilumnoid genera for species previously assigned to *Planopilumnus* Balss, 1933, Zootaxa 2392, pp. 33-61: 34-35. <https://doi.org/10.11646/zootaxa.2392.1.2>
188. Nguyen, H. D., and M. Byrne. 2014. Early benthic juvenile *Parvulastra exigua* (Asteroidea) are tolerant to extreme acidification and warming in its intertidal habitat. Journal of Experimental Marine Biology and Ecology 453: 36-42. <https://doi.org/10.1016/j.jembe.2013.12.007>
189. Nie, H., Y. Lu, H. Liu, H. Yan, L. Zhao, F. Yang, and X. Yan. 2016. Seasonal Variations in Biochemical Composition of the Clam *Dosinia corrugatein* Relation to the Reproductive Cycle and Environmental Conditions. Journal of Shellfish Research 35: 369-377. <https://doi.org/10.2983/035.035.0211>
190. Nielsen, B.J. 1963. Studies of the genus *Katelysia* Romer 1857 (Mollusca, Lamellibranchiata). Mem. Nat. Mus. Vict. 26: 219-257. <https://doi.org/10.24199/j.mmv.1964.26.12>
191. Nimbs, M. J., and S. D. A. Smith. 2016. An illustrated inventory of the sea slugs of New South Wales, Australia (Gastropoda: Heterobranchia). The Royal Society of Victoria. 128: 44-113. <https://doi.org/10.1071/RS16011>`
192. Nimbs, M. J., R. C. Willan, and S. D. A. Smith. 2017. A Historical Summary of the Distribution and Diet of Australian Sea Hares (Gastropoda: Heterobranchia: Aplysiidae). Zool Stud 56: e35.
193. Nye, P. A. 1974. Burrowing and burying by the crab *Macrophthalmus hirtipes*. New Zealand Journal of Marine and Freshwater Research 8: 243-254. <https://doi.org/10.1080/00288330.1974.9515502>
194. O’loughlin, P.M., Paulay, G., Vandenspiegel, Samyn, Y. 2007. New *Holothuria* species from Australia (Echinodermata: Holothuroidea: Holothuriidae), with comments on the origin of deep and cool holothuriids. Memoirs of Museum Victoria. 64: 35-32. <https://doi.org/10.24199/j.mmv.2007.64.5>
195. O'Hara, T., and M. Byrne. 2017. Australian Echinoderms: Biology, Ecology and Evolution. CSIRO Publishing and ABRS, Melbourne and Canberra. Pp. 612.
196. Panampunnayil, S.U. 1986. New mysids from the South Australian coastal waters: *Paranchialina secunda* sp. nov.; *Leptomysis longisquama sp.* nov. and *Doxomysis johnsoni* sp. nov. Journal of Plankton Research. 8: 1183-1195. <https://doi.org/10.1093/plankt/8.6.1183>
197. Parish, J. 1981. Reproductive ecology of Naididae (Oligochaeta). Hydrobiologia. 83: 115-123. <https://doi.org/10.1007/BF02187156>
198. Passos, F. v. D., O. Domaneschi, and A. F. Sartori. 2004. Biology and functional morphology of the pallial organs of the Antarctic bivalve *Mysella charcoti* (Lamy, 1906) (Galeommatoidea: Lasaeidae). Polar Biology 28: 372-380. <https://doi.org/10.1007/s00300-004-0702-5>
199. Pasupathi, K., and T. Kannupandi. 1988a. The complete larval development of the Mangrove Ocypodid crab *Macrophthalmus depressus* Ruppell, 1830 (Brachyura: Macrophthalminae) reared in the laboratory. Journal of Natural History 22: 1533-1544. <https://doi.org/10.1080/00222938800770951>
200. Pasupathi, K., and T. Kannupandi. 1988b. Larval development of *Macrophthalmus erato* De man, 1887 (Brachyura: Ocypodidae). Hydrobiologia. 169: 327-338. <https://doi.org/10.1007/BF00007556>
201. Peart, R.A. 2007a. A review of Australian Cymadusa (Crustacea: Amphipoda: Ampithoidae) with descriptions of eight new species. Zootaxa, 1540: 1–53. <https://doi.org/10.11646/zootaxa.1540.1.1>
202. Peart, R.A. 2007b. A review of Australian species of Ampithoe Leach, 1814 (Crustacea: Amphipoda: Ampithoidae) with descriptions of seventeen new species. Zootaxa, 1566: 1–95. <https://doi.org/10.11646/zootaxa.1566.1.1>
203. Peharda, M., M. Crnčević, D. Ezgeta-Balić, and M. Pećarević. 2013. Reproductive cycle of *Glycymeris nummaria* (Mollusca: Bivalvia) from Mali Ston Bay, Adriatic Sea, Croatia. Scientia Marina 77: 293-300. <https://doi.org/10.3989/scimar.03722.10A>
204. Penn, J. 1980. Spawning and fecundity of the western king prawn, *Penaeus lastisulcatus* Kishinouye, in Western Australia Waters. Aust. J. Mar. Freshwater. Res. 31: 21-35. <https://doi.org/10.1071/MF9800021>
205. Pennec, M.L. and Beninger, P.G. 2000. Reproductive characteristics and strategies of reducing-system bivalves. Comparative biochemistry and physiology Part A. 126: 1-16. <https://doi.org/10.1016/S0742-8413(00)00100-6>
206. Petraitis, P.S. 1985. Females inhibit males’ propensity to develop into simultaneous hermaphrodites in *Capitella* species I (Polychaeta). Biological Bulletin. 168: 395-402. <https://doi.org/10.2307/1541520>
207. Philipp, E., Brey, T., Voigt, M., Abele., D. 2008. Growth and age of *Laternula elliptica* populations in Potter Cove, King-George Island. Reports on Polar in Marine Research. 571: 216-222.
208. Pilsbry, H.A. and Olsson, A.A. 1952. Vitrinellidae of the Panamic Province: II. Proceedings of the Academy of Natural Sciences of Philadelphia. 104: 35-88.
209. Pinder, A. 2010. Tools for identifying selected Australian aquatic oligochaetes (Clitellata: Annelida). Museum Victoria Science Reports 13: 1-26. <https://doi.org/10.24199/j.mvsr.2010.13>
210. Ponder, W. F. 1975. Notes on The Synonymy of Four Australian Tellinids (Mollusca: Bivalvia). Journal of the Malacological Society of Australia 3:111-119. <https://doi.org/10.1080/00852988.1975.10673887>
211. Ponder, W. F., and R. G. Creese. 2013. A revision of the Australian species of *Notoacmea*, *Collisella* and *Patelloida* (Mollusca: Gastropoda: Acmaeidae). Journal of the Malacological Society of Australia 4: 167-208. <https://doi.org/10.1080/00852988.1980.10673927>
212. Ponder, W.F. 1984. A review of the genera of the Rissoidae (Mollusca: Mesogastropoda: Rissoacea). Records of the Australian Museum. Suppl. 4: 1-221. <https://doi.org/10.3853/j.0812-7387.4.1985.100>
213. Ponder, W.F., Clark, G.A., Miller, A.C. 1999. A new genus and two new species of Hydrobiidae (Mollusca: Gastropoda: Caenogastropoda) from south Western Australia. Journal of the Royal Society of Western Australia. 82: 109-120.
214. Ponder, W.F., Colgan, D.J., Clark, G.A. 1991. The morphology, taxonomy and genetic structure of *Tatea* (Mollusca: Gastropoda: Hydrobiidae), estuarine snails from temperate Australia. Aust. J. Zool. 39: 447-97. <https://doi.org/10.1071/ZO9910447>
215. Poore, C.G.B. and Brandi, A. 2001. *Plakarthrium australiense*, a third species of Plakarthriidae (Crustacea: Isopoda). Memoirs of Museum Victoria. 58: 373-382. <https://doi.org/10.24199/j.mmv.2001.58.20>
216. Poore, G. C. B., and H. M. L. Ton. 1986. *Mesanthura* (Crustacea: Isopoda: Anthuridae) from southeastern Australia. Memoirs of the Museum of Victoria 47: 87-104. <https://doi.org/10.24199/j.mmv.1986.47.04>
217. Poore, G. C. B., and H. M. L. Ton. 1993. Idoteidae of Australia and New Zealand (Crustacea: Isopoda: Valvifera). Invertebr. Taxon. 7: 197-278. <https://doi.org/10.1071/IT9930197>
218. Poore, G. C. B., and H. M. Lewton. 1988. Antheluridae, a new family of Crustacea (Isopoda: Anthuridea) with new species from Australia. Journal of Natural History 22: 489-506. <https://doi.org/10.1080/00222938800770341>
219. Poore, G.B. and Lowry J.K. 1997. New Ampithoid Amphipods from Port Jackson, New South Wales, Australia (Crustacea: Amphipoda: Ampithoidae). Invertebrate Taxonomy. 11: 897-941. <https://doi.org/10.1071/IT95045>
220. Poore, G.C.B. 2001. Isopoda Valvifera: Diagnoses and relationship of the families. Journal of Crustacean Biology. 21: 205-230. <https://doi.org/10.1163/20021975-99990118>
221. Poore, G.C.B. 2015. *Halearcturus*, a new genus of Antarcturidae Poore, 2001 (Crustacea: Isopoda: Valvifera) with a key to genera of the family. Memoirs of Museum Victoria. 73: 13-18. <https://doi.org/10.24199/j.mmv.2015.73.02>
222. Prato, E., and Biandolino, F. 2006. Life history of the amphipod *Corophium insidiosum* (Crustacea: Amphipoda) from Mar Piccolo (Ionian Sea, Italy). Scientia Marina. 70: 355-362. <https://doi.org/10.3989/scimar.2006.70n3355>
223. Price, R.M., Gosliner, T.M., Valdes, A. 2011. Systematics and phylogeny of *Philine* (Gastropoda: Opisthobranchia), with emphasis on the *Philine aperta* species complex. The Veliger. 51: 1-58.
224. Przeslawski, R. 2011. Notes on the egg capsule and variable embryonic development of *Nerita melanotragus* (Gastropoda: Neritidae). Molluscan Research. 31: 152-158.
225. Rainer, S., and P. A. Hutchings, 1977. Nephtyidae (Polychaeta: Errantia) from Australia. Records of the Australian Museum. 31: 307–347. <https://doi.org/10.3853/j.0067-1975.31.1977.216>
226. Rayner, S. M. 1983. Distribution of teredinids (Mollusca: Teredinidae) in Papua New Guinea. Records of the Australian Museum 35: 61-76. <https://doi.org/10.3853/j.0067-1975.35.1983.302>
227. Reid, R. G. B., and D. G. Brand. 1987. Observations on Australian Solemyidae. Journal of the Malacological Society of Australia 8: 41-50. <https://doi.org/10.1080/00852988.1987.10673991>
228. Rice, M.E. and Pilger, J.F. ND. 1975. Chapter 10 Sipuncula. *In:* Reproductive Biology of Invertebrates, A. C. Giese and J. C. Pearse (eds.). Academic Press, London. Pp. 297-310.
229. Richardson, C. A., P. R. Kingsley-Smith, R. Seed, and E. Chatzinikolaou. 2005. Age and growth of the naticid gastropod *Polinices pulchellus* (Gastropoda: Naticidae) based on length frequency analysis and statolith growth rings. Marine Biology 148:319-326. <https://doi.org/10.1007/s00227-005-0072-8>
230. Ridgway, S.A., Reid, D.G., Taylor, J.D., Branch, G.M., Hodgson, A.N. 1998. A cladistic phylogeny of the family Patellidae (Mollusca: Gastropoda). Phil. Trans. R. Soc. Lond. B. 353: 1645-1671. <https://doi.org/10.1098/rstb.1998.0316>
231. Roberts, D. 1984. The genus *Katelysia* (Bivalvia: Veneridae) in southern Australia. Journal of the Malacological Society of Australia 6: 191-204. <https://doi.org/10.1080/00852988.1984.10673969>
232. Roediger, L.M. and Bolton, T.F. 2008. Abundance and distribution of South Australia’s endemic sea star, *Parvulastra parvivipara* (Asteroidea: Asterinidae). Marine and Freshwater Research. 59: 205-213. <https://doi.org/10.1071/MF07084>
233. Rolan, E. and Sellanes, J. 2004. Una nueva especie del genero *Vitrinella* de Chile (Gastropoda, Vitrinellidae). Iberus. 22: 167-172.
234. Rudman, W.B. 1972. The genus *Philine* (Opistobranchia, Gastropoda). Proc. Malac. Soc. Lond. 40: 171-187.
235. Rumbold, C. E., E. D. Spivak, and S. M. Obenat. 2012. Life history of *Tanais dulongii* (Tanaidacea: Tanaidae) in an intertidal flat in the southwestern Atlantic. Journal of Crustacean Biology 32: 891-898. <https://doi.org/10.1163/1937240X-00002094>
236. Rumbold, C. E., S. M. Obenat, and E. D. Spivak. 2015. Comparison of life history traits of *Tanais dulongii* (Tanaidacea: Tanaididae) in natural and artificial marine environments of the south-western Atlantic. Helgoland Marine Research 69: 231-242. <https://doi.org/10.1007/s10152-015-0432-9>
237. Sainte-Marie, B. 1986. Feeding and swimming of lysianassid amphipods in a shallow cold-water bay. Marine Biology. 91: 219-229. <https://doi.org/10.1007/BF00569437>
238. Sakai, K. 2000. A new species of *Neocallichirus, N. angelikae*, from South Australia (Decapoda: Callianassidae). Mitt. Hamb. Zool. Mus. Inst. 97: 91-98.
239. Satheesh, S., and S. G. Wesley. 2009. Breeding biology of the barnacle *Amphibalanus amphitrite* (Crustacea: Cirripedia): influence of environmental factors in a tropical coast. Journal of the Marine Biological Association of the United Kingdom 89:1203-1208. <https://doi.org/10.1017/S0025315409000228>
240. Saunders, B. 2009. Shores and shallows of Coffin Bay: An identification guide. 2^nd^ edition. Eyre Peninsula Natural Resources. Australia.
241. Selvakumaraswamy, P. and Byrne, M. 1995. Reproductive cycle of two populations of *Ophionereis schayeri* (Ophiuroidea) in New South Wales. Marine biology. 124: 85-97. <https://doi.org/10.1007/BF00349150>
242. Selvakumaraswamy, P. and Byrne, M. 2000. Reproduction, spawning, and development of 5 ophiuroids from Australia and New Zealand. Invertebrate Biology. 119: 394-402. <https://doi.org/10.1111/j.1744-7410.2000.tb00109.x>
243. Sigwart, J. D., I. Stoeger, T. Knebelsberger, and E. Schwabe. 2013. Chiton phylogeny (Mollusca: Polyplacophora) and the placement of the enigmatic species *Choriplax grayi* (H. Adams & Angas). Invertebrate Systematics 27. <https://doi.org/10.1071/IS13013>
244. Simons, M. J., and M. B. Jones. 1981. Population and reproductive biology of the mud crab, *Macrophthalmus hirtipes* (Jacquinot, 1853) (Ocypodidae), from marine and estuarine habitats. Journal of Natural History 15:981-994. <https://doi.org/10.1080/00222938100770731>
245. Slater, J.M. 2009. The sea cucumber *Australostichopus mollis*: Juvenile feeding ecology habitat. PhD thesis. University of Auckland.
246. Slattery, P.N. 1985. Life history of infaunal amphipods from subtidal sands of Monterey Bay California. Journal of Crustacean Biology. 5: 635-649. <https://doi.org/10.2307/1548241>
247. Smith, B. J., and R. C. Robertson. 1970. Catalogue of chiton (Amphineura, Mollusca) types in the National Museum of Victoria, Australia. Memoirs of the National Museum of Victoria 31:81-89. <https://doi.org/10.24199/j.mmv.1970.31.09>
248. Smith, M.J. and Williams, W.D. 1983. Reproduction cycles in some freshwater amphipods in Southern Australia. Australian Museum Memoir. 18: 183-194. <https://doi.org/10.3853/j.0067-1967.18.1984.384>
249. Southgate, P.C. and Lucas, J.L. 2008. The Pearl Oyster. Elsevier, Amsterdam. 554 pp. <https://doi.org/10.1016/B978-0-444-52976-3.00007-3>
250. Spano, C., N. and Häussermann, V. 2017. *Anthopleura radians*, a new species of sea anemone (Cnidaria: Actiniaria: Actiniidae) from northern Chile, with comments on other species of the genus from the South Pacific Ocean. Biodiversity and Natural History. 3: 1-11.
251. Spano, C., N. Rozbaczylo, V. Häussermann, and R. Bravo. 2013. Redescription of the sea anemones *Anthopleura hermaphroditica* and *Bunodactis hermaphroditica* (Cnidaria: Anthozoa: Actiniaria) from Chile. Revista de biología Marina y Oceanografía 48: 521-534. <https://doi.org/10.4067/S0718-19572013000300010>
252. Stapleton, K. L., M. Long, and F. L. Bird. 2001. Comparative feeding ecology of two spatially coexisting species of ghost shrimp, *Biffarius arenosus* and *Trypaea australiensis* (Decapoda: Callianassidae). Ophelia 55: 141-150. <https://doi.org/10.1080/00785236.2001.10409481>
253. Styan, C.A., McCluskey, C.F., Sun, Y., Kupriyanova, E.K. 2017. Cryptic sympatric species across the Australian range of the global estuarine invader *Ficopomatus enigmatus* (Fauvel, 1923) (Serpulidae, Annelida). Aquatic Invasions. 12: 53-65. <https://doi.org/10.3391/ai.2017.12.1.06>
254. Stoddart, H.E., and Lowry, J.K. 2010. The family Aristiidae (Crustacea: Amphipoda: Lysianassoidea) in Australian waters. Zootaxa. 2549: 31-53. <https://doi.org/10.11646/zootaxa.2549.1.2>
255. Subida, M. D., M. R. Cunha, and M. H. Moreira. 2005. Life history, reproduction, and production of *Gammarus chevreuxi* (Amphipoda: Gammaridae) in the Ria de Aveiro, northwestern Portugal. Journal of the North American Benthological Society 24: 82-100. [https://doi.org/10.1899/0887-3593(2005)024<0082:LHRAPO>2.0.CO;2](https://doi.org/10.1899/0887-3593(2005)024%3C0082:LHRAPO%3E2.0.CO;2)
256. Swanson, E.L., de Nys, R., Huggett, M.J., Green, J.K., Steinberg, P.D. 2006. *In situ* quantification of natural settlement cue and recruitment of the Australian sea urchin *Holopneustes purpurascens*. Marine Ecology Progress Series. 314: 1-14. <https://doi.org/10.3354/meps314001>
257. Takeuchi, I., and Hirano, R. 1991. Growth and reproduction of *Caprella danilevskii* (Crustacea: Amphipoda) reared in the laboratory. Marine Biology. 110: 391-397. <https://doi.org/10.1007/BF01344358>
258. Tan, S.K. and Low, M.E. 2013. Singapore Mollusca: 2. The Family Trapezidae with a new record of *Glossocardia obesa* (Bivalvia: Veneroidea: Arcticoidea). Nature in Singapore. 6: 247-256.
259. Tanaka, K. 2007. Life history of gnathiid isopods-current knowledge and future directions. Plankton and Benthos Research 2: 1-11. <https://doi.org/10.3800/pbr.2.1>
260. Taylor, J. D., E. A. Glover, L. Smith, P. Dyal, and S. T. Williams. 2011. Molecular phylogeny and classification of the chemosymbiotic bivalve family Lucinidae (Mollusca: Bivalvia). Zoological Journal of the Linnean Society 163: 15-49. <https://doi.org/10.1111/j.1096-3642.2011.00700.x>
261. ter Poorten, J. J., L. A. Kirkendale, and J.-M. Poutiers. 2017. The Cardiidae (Mollusca: Bivalvia) of tropical northern Australia: A synthesis of taxonomy, biodiversity and biogeography with the description of four new species. Records of the Western Australian Museum 32: 101-190. <https://doi.org/10.18195/10.18195/issn.0312-3162.32(2).2017.101-190>
262. ter Poorten, J.J. 2013. Revision of the recent species of the genus *Nemocardium* Meek, 1876 (Bivalvia, Cardiidae) with the descriptions of three new species. Basteria. 77: 45-73.
263. Tevesz, M.J.S. 1975. Structure and habits of the ‘living fossil’ pelecypod *Neotrigonia*. Lethaia. 8: 321-327. <https://doi.org/10.1111/j.1502-3931.1975.tb00937.x>
264. Thiyagarajan, V., O. S. Hung, J. M. Y. Chiu, R. S. S. Wu, and P. Y. Qian. 2005. Growth and survival of juvenile barnacle *Balanus amphitrite*: interactive effects of cyprid energy reserve and habitat. Marine Ecology Progress Series 299 :229-237. <https://doi.org/10.3354/meps299229>
265. Too, C. C., C. Carlson, P. J. Hoff, and M. A. Malaquias. 2014. Diversity and systematics of Haminoeidae gastropods (Heterobranchia: Cephalaspidea) in the tropical West Pacific Ocean: new data on the genera *Aliculastrum, Atys, Diniatys* and *Liloa*. Zootaxa 3794: 355-392. <https://doi.org/10.11646/zootaxa.3794.3.3>
266. Tsoi, K.-H. 1999. Biology of the amphipod *Hyale* sp. (Gammaridea: Hyalidae). Master thesis. The Chinese University of Hong Kong.
267. Tsoi, K.-H. and Chu, K.-H. 2005. Sexual dimorphism and reproduction of the amphipod *Hyale crassicornis* Haswell (Gammaridea: Hyalidae). Zoological Studies. 44: 382-392.
268. Tuwo, A., and C. Conand. 1992. Reproductive biology of the holothurian *Holothuria forskali* (Echinodermata). Journal of the Marine Biological Association of the United Kingdom 72: 745-758. <https://doi.org/10.1017/S0025315400060021>
269. Underwood, A.J. 1975. Comparative studies on the biology of *Nerita atramentosa* Reeve, *Bembicium nanum* (Lamarck) and *Cellana tramoserica* (Sowerby) (Gastropoda: Prosobranchia) in S. E. Australia. J. Exp. Mar. Biol. Ecol. 18: 153-172. <https://doi.org/10.1016/0022-0981(75)90071-4>
270. Underwood, A.J., Creese, R.G. 1976. Observations on the biology of the trochid gastropod *Austrocochlea constricta* (Lamarck) (Prosobranchia). 2. The effects of available food on shellbanding pattern. J. Exp. Mar. Biol. Ecol. 23: 229-240. <https://doi.org/10.1016/0022-0981(76)90022-8>
271. Unno, J. 2000. Occurrence of *Amphipholis quamata* (Echinodermanta: Ophiuroidea) in relation to habitat in the Leschenault Inlet estuary. Journal of the Royal Society of Western Australia. 83: 475-480.
272. Van Der Molen, S., M. Kroeck, and N. Ciocco. 2007. Reproductive cycle of the southern geoduck clam, *Panopea abbreviate* (Bivalvia: Hiatellidae), in north Patagonia, Argentina. Invertebrate Reproduction & Development. 50: 75-84. <https://doi.org/10.1080/07924259.2007.9652230>
273. Vendrasco, M.J., Fernandez., C.Z., Eernisse, D.J., Runnegar, B. 2008. Aesthete canal morphology in the Mopaliidae (Polyplacophora). Amer. Malac. Bull. 25: 51-69. <https://doi.org/10.4003/0740-2783-25.1.51>
274. Vokes, E. H. 1993. Review of the muricine subgenus *Pterynotus* (Pterochelus) in Australia. Journal of the Malacological Society of Australia 14: 83-105. <https://doi.org/10.1080/00852988.1993.10674045>
275. Wada, S., Kitaoka, H., Goshima, S. 2000. Reproduction of the hermit crab *Pagurus lanuginosus* and comparison of reproductive traits among sympatric species. Journal of Crustacean Biology. 20: 474-478. [https://doi.org/10.1651/0278-0372(2000)020[0474:ROTHCP]2.0.CO;2](https://doi.org/10.1651/0278-0372(2000)020%5b0474:ROTHCP%5d2.0.CO;2)
276. Walker, T.M., and Poore, G.C.B. 2003. Rediagnosis of *Palaemon* and differentiation of southern Australian species (Crustacea: Decapoda: Palaemonidae). Memoirs of Museum Victoria 60: 243–256. <https://doi.org/10.24199/j.mmv.2003.60.25>
277. Walker-Smith, G.K. and Poore, G.C.B. 2001. A phylogeny of the Leptostraca (Crustacea) with keys to families and genera. Memoirs of Museum Victoria. 58: 383-410. <https://doi.org/10.24199/j.mmv.2001.58.21>
278. Wall, A. R., N. L. Bruce, and R. Wetzer. 2015. Status of *Exosphaeroma amplicauda* (Stimpson, 1857), *E. aphrodita* (Boone, 1923) and description of three new species (Crustacea, Isopoda, Sphaeromatidae) from the north-eastern Pacific. Zookeys: 11-58. <https://doi.org/10.3897/zookeys.504.8049>
279. Wells, F. E., and T. J. Threlfall. 1982. Reproductive strategies of *Hydrococcus brazieri* (Tenison Woods, 1876) and *Arthritica semen* (Menke, 1843) in Peel Inlet, Western Australia. Journal of the Malacological Society of Australia 5: 157-166. <https://doi.org/10.1080/00852988.1982.10673947>
280. Weslawski, J.M. and Legezynska, J.M. 2002. Life cycles of some Arctic amphipods. Polish Polar Research. 23: 253-264.
281. Williams, W.D. 1983. On the ecology of *Haloniscus searlei* (Isopoda: Oniscoidea), an inhabitant of Australian salt lakes. Hydrobiologia. 105: 137-142. <https://doi.org/10.1007/BF00025183>
282. Williamson, J.E., and Steinberg, P.D. 2002. Reproductive cycle of the sea urchin *Holopneustes purpurascens* (Temnopleuridae: Echinodermata). Marine Biology. 140: 519-532. <https://doi.org/10.1007/s00227-001-0716-2>
283. Wilson, G.D., Thistle, D. and Hessler, R.R. 1976. The Plakarthriidae (Isopoda: Flabellifera): déjà vu. Zoological Journal of the Linnean Society. 58: 331-343. <https://doi.org/10.1111/j.1096-3642.1976.tb01003.x>
284. Wilson, G.D.F. and Wagele, J.-W. 1994. Review of the Family Janiridae (Crustacea: Isopoda: Asellota). Invertebr. Taxon. 8: 683-747. <https://doi.org/10.1071/IT9940683>
285. Wolf, B.M. and White, R.W.G. 1995. Age and growth of the queen scallop, *Equichlamys bifrons*, in the D’Entrecasteaux Channel and Huon River Estuary, Tasmania. Mar. Freshwater Res. 46: 1127-35. <https://doi.org/10.1071/MF9951127>
286. Wong, Y.M. and More, P.G. 1996. Observations on the activity and life history of the scavenging isopod *Natatolana borealis* Lilljeborg (Isopoda: Cirolanidae) from Loch Fyne, Scotland. Estuarine, Coastal and Shelf Science. 42: 247-262. <https://doi.org/10.1006/ecss.1996.0018>
287. Woods, J.E.T. 1987. On some Tasmanian Patellidae. Proc. Roy. Soc. Tasm. 43-58.
288. Yokoyama, L. Q., and A. C. Z. Amaral. 2011. Recruitment and growth variation of *Ophionereis reticulata* (Echinodermata: Ophiuroidea). Invertebrate Reproduction & Development 55: 73-81. <https://doi.org/10.1080/07924259.2011.553402>
289. Zacharin, W.F. 1995. Growth, reproduction, and recruitment of the Doughboy scallop, *Mimachlamys asperrimus* (Lamarck) in the D’entrecasteaux Channel, Tasmania, Australia. Master thesis. University of Tasmania.
290. Zamora, L. N., and A. G. Jeffs. 2013. A Review of the Research on the Australasian Sea Cucumber, *Australostichopus mollis* (Echinodermata: Holothuroidea) (Hutton 1872), with Emphasis on Aquaculture. Journal of Shellfish Research 32: 613-627. <https://doi.org/10.2983/035.032.0301>
291. Zegaoula, B., Beldi, H., Draredja, B., Soltani, N. 2016. Reproduction of *Patella rustica* (Mollusca, Gastropoda) in the gulf of Annaba (Algeria, Mediterranean South Western). Advances in Environmental Biology. 10: 42-50.

**Table S4.** List of the taxa included in The South Australian Macrobenthic Traits database.

| Phylum | Class | Order | Family | Species | AphiaID |
| --- | --- | --- | --- | --- | --- |
| Annelida | Oligochaeta |  |  |  | 2036 |
| Annelida | Polychaeta | Amphinomida | Amphinomidae |  | 960 |
| Annelida | Polychaeta | Amphinomida | Euphrosinidae |  | 961 |
| Annelida | Polychaeta | Eunicida | Dorvilleidae |  | 971 |
| Annelida | Polychaeta | Eunicida | Eunicidae |  | 966 |
| Annelida | Polychaeta | Eunicida | Lumbrineridae |  | 967 |
| Annelida | Polychaeta | Eunicida | Onuphidae |  | 965 |
| Annelida | Polychaeta | Phyllodocida | Aphroditidae |  | 938 |
| Annelida | Polychaeta | Phyllodocida | Glyceridae |  | 952 |
| Annelida | Polychaeta | Phyllodocida | Goniadidae |  | 953 |
| Annelida | Polychaeta | Phyllodocida | Nephtyidae |  | 956 |
| Annelida | Polychaeta | Phyllodocida | Nephtyidae | *Aglaophamus australiensis* | 547399 |
| Annelida | Polychaeta | Phyllodocida | Nereididae |  | 22496 |
| Annelida | Polychaeta | Phyllodocida | Nereididae | *Australonereis ehlersi* | 333062 |
| Annelida | Polychaeta | Phyllodocida | Nereididae | *Neanthes vaalii* | 334112 |
| Annelida | Polychaeta | Phyllodocida | Nereididae | *Simplisetia aequisetis* | 334804 |
| Annelida | Polychaeta | Phyllodocida | Phyllodocidae |  | 931 |
| Annelida | Polychaeta | Phyllodocida | Phyllodocidae | *Phyllodoce novaehollandiae* | 330618 |
| Annelida | Polychaeta | Phyllodocida | Polynoidae |  | 939 |
| Annelida | Polychaeta | Phyllodocida | Sigalionidae |  | 943 |
| Annelida | Polychaeta | Phyllodocida | Syllidae |  | 948 |
| Annelida | Polychaeta | Phyllodocida | Syllidae | *Sphaerosyllis sp.* | 129677 |
| Annelida | Polychaeta | Sabellida | Oweniidae |  | 975 |
| Annelida | Polychaeta | Sabellida | Sabellidae | *Euchone variabilis* | 327558 |
| Annelida | Polychaeta | Sabellida | Sabellidae |  | 985 |
| Annelida | Polychaeta | Sabellida | Serpulidae |  | 988 |
| Annelida | Polychaeta | Sabellida | Serpulidae | *Ficopomatus enigmaticus* | 130988 |
| Annelida | Polychaeta | Spionida | Spionidae | *Boccardiella limnicola* | 872564 |
| Annelida | Polychaeta | Spionida | Spionidae |  | 913 |
| Annelida | Polychaeta | Terebellida | Ampharetidae |  | 981 |
| Annelida | Polychaeta | Terebellida | Cirratulidae |  | 919 |
| Annelida | Polychaeta | Terebellida | Cirratulidae | *Cirriformia sp.* | 129245 |
| Annelida | Polychaeta | Terebellida | Flabelligeridae |  | 976 |
| Annelida | Polychaeta | Terebellida | Pectinariidae |  | 980 |
| Annelida | Polychaeta | Terebellida | Terebellidae |  | 982 |
| Annelida | Polychaeta | Terebellida | Trichobranchidae |  | 983 |
| Annelida | Polychaeta |  | Arenicolidae |  | 922 |
| Annelida | Polychaeta |  | Capitellidae |  | 921 |
| Annelida | Polychaeta |  | Magelonidae |  | 914 |
| Annelida | Polychaeta |  | Maldanidae |  | 923 |
| Annelida | Polychaeta |  | Oenonidae |  | 22610 |
| Annelida | Polychaeta |  | Opheliidae |  | 924 |
| Annelida | Polychaeta |  | Orbiniidae |  | 902 |
| Annelida | Polychaeta |  | Paraonidae |  | 903 |
| Annelida | Polychaeta |  | Sabellariidae |  | 979 |
| Arthropoda | Hexanauplia | Sessilia | Austrobalanidae | *Austrominius adelaidae* | 535009 |
| Arthropoda | Hexanauplia | Sessilia | Balanidae | *Amphibalanus amphitrite* | 421137 |
| Arthropoda | Hexanauplia | Sessilia | Balanidae | *Balanus sp.* | 106122 |
| Arthropoda | Hexanauplia | Sessilia | Chthamalidae | *Chamaesipho tasmanica* | 733183 |
| Arthropoda | Insecta | Diptera | Ceratopogonidae |  | 150940 |
| Arthropoda | Insecta | Diptera | Chironomidae |  | 118100 |
| Arthropoda | Insecta | Diptera | Dolichopodidae |  | 150930 |
| Arthropoda | Malacostraca | Amphipoda |  |  | 1135 |
| Arthropoda | Malacostraca | Amphipoda | Amaryllididae |  | 236743 |
| Arthropoda | Malacostraca | Amphipoda | Ampeliscidae |  | 101364 |
| Arthropoda | Malacostraca | Amphipoda | Ampeliscidae | *Byblis sp.* | 101446 |
| Arthropoda | Malacostraca | Amphipoda | Ampithoidae |  | 101366 |
| Arthropoda | Malacostraca | Amphipoda | Aoridae |  | 101368 |
| Arthropoda | Malacostraca | Amphipoda | Aristiidae |  | 236740 |
| Arthropoda | Malacostraca | Amphipoda | Caprellidae |  | 101361 |
| Arthropoda | Malacostraca | Amphipoda | Caprellidae | *Caprella danilevskii* | 101827 |
| Arthropoda | Malacostraca | Amphipoda | Caprellidae | *Paraproto spinosa* | 431139 |
| Arthropoda | Malacostraca | Amphipoda | Corophiidae |  | 101376 |
| Arthropoda | Malacostraca | Amphipoda | Cyproideidae |  | 236744 |
| Arthropoda | Malacostraca | Amphipoda | Dexaminidae |  | 101378 |
| Arthropoda | Malacostraca | Amphipoda | Eusiridae |  | 101380 |
| Arthropoda | Malacostraca | Amphipoda | Gammaridae |  | 101383 |
| Arthropoda | Malacostraca | Amphipoda | Haustoriidae | *Haustorius sp.* | 101546 |
| Arthropoda | Malacostraca | Amphipoda | Hyalidae |  | 101385 |
| Arthropoda | Malacostraca | Amphipoda | Isaeidae |  | 101388 |
| Arthropoda | Malacostraca | Amphipoda | Lysianassidae |  | 101395 |
| Arthropoda | Malacostraca | Amphipoda | Pardaliscidae | Pardaliscidae | 101401 |
| Arthropoda | Malacostraca | Amphipoda | Perthiidae |  | 548463 |
| Arthropoda | Malacostraca | Amphipoda | Phoxocephalidae |  | 101403 |
| Arthropoda | Malacostraca | Cumacea |  |  | 1137 |
| Arthropoda | Malacostraca | Cumacea | Bodotriidae | *Cyclaspis spilotes* | 181750 |
| Arthropoda | Malacostraca | Cumacea | Bodotriidae | *Cyclaspis tribulis* | 181762 |
| Arthropoda | Malacostraca | Cumacea | Ceratocumatidae |  | 110379 |
| Arthropoda | Malacostraca | Cumacea | Diastylidae |  | 110380 |
| Arthropoda | Malacostraca | Cumacea | Gynodiastylidae | *Gynodiastylis truncatifrons* | 182189 |
| Arthropoda | Malacostraca | Cumacea | Gynodiastylidae | *Litogynodiastylis turgida* | 489289 |
| Arthropoda | Malacostraca | Decapoda | Callianassidae | *Biffarius arenosus* | 477612 |
| Arthropoda | Malacostraca | Decapoda | Callianassidae | *Biffarius limosus* | NA |
| Arthropoda | Malacostraca | Decapoda | Callianassidae | *Neocallichirus angelikae* | 477724 |
| Arthropoda | Malacostraca | Decapoda | Callianassidae |  | 106800 |
| Arthropoda | Malacostraca | Decapoda | Caridea |  | 106674 |
| Arthropoda | Malacostraca | Decapoda | Corystidae | *Gomeza bicornis* | 209576 |
| Arthropoda | Malacostraca | Decapoda | Dromiidae | *Stimdromia lateralis* | 440108 |
| Arthropoda | Malacostraca | Decapoda | Galatheidae | *Galathea australiens* | 210092 |
| Arthropoda | Malacostraca | Decapoda | Hymenosomatidae | *Halicarcinus ovatus* | 441784 |
| Arthropoda | Malacostraca | Decapoda | Leucosiidae | *Bellidilia laevis* | 441104 |
| Arthropoda | Malacostraca | Decapoda | Leucosiidae | *Bellidilia undecimspinosa* | 441105 |
| Arthropoda | Malacostraca | Decapoda | Leucosiidae | *Cryptocnemus vincentianus* | 441066 |
| Arthropoda | Malacostraca | Decapoda | Leucosiidae | *Ebalia intermedia* | 441125 |
| Arthropoda | Malacostraca | Decapoda | Litocheiridae | *Litocheira bispinosa* | 441005 |
| Arthropoda | Malacostraca | Decapoda | Macrophthalmidae | *Tasmanoplax latifrons* | 558102 |
| Arthropoda | Malacostraca | Decapoda | Paguridae | *Lophopagurus (Lophopagurus) nanus* | 366494 |
| Arthropoda | Malacostraca | Decapoda | Paguridae |  | 106738 |
| Arthropoda | Malacostraca | Decapoda | Palaemonidae | *Palaemon intermedius* | 514452 |
| Arthropoda | Malacostraca | Decapoda | Penaeidae | *Metapenaeopsis novaeguineae* | 377510 |
| Arthropoda | Malacostraca | Decapoda | Penaeidae | *Penaeus latisulcatus* | 210372 |
| Arthropoda | Malacostraca | Decapoda | Piluminidae | *Pilumnopeus serratifrons* | 395025 |
| Arthropoda | Malacostraca | Decapoda | Planopilumnidae | *Rathbunaria orientalis* | 558010 |
| Arthropoda | Malacostraca | Decapoda | Portunidae | *Portunus pelagicus* | 1061754 |
| Arthropoda | Malacostraca | Decapoda | Varunidae | *Brachynotus spinosus* | 444761 |
| Arthropoda | Malacostraca | Decapoda | Varunidae | *Helograpsus haswellianus* | 444729 |
| Arthropoda | Malacostraca | Decapoda | Varunidae | *Paragrapsus gaimardii* | 444743 |
| Arthropoda | Malacostraca | Isopoda | Antarcturidae |  | 174627 |
| Arthropoda | Malacostraca | Isopoda | Antheluridae |  | 118243 |
| Arthropoda | Malacostraca | Isopoda | Anthuridae |  | 118244 |
| Arthropoda | Malacostraca | Isopoda | Anthuridae | *Mesanthura maculata* | 211375 |
| Arthropoda | Malacostraca | Isopoda | Arcturidae | *Neastacilla deducta* | 261418 |
| Arthropoda | Malacostraca | Isopoda | Arcturidae | *Parastacilla bakeri* | 261924 |
| Arthropoda | Malacostraca | Isopoda | Cirolanidae | *Cirolana cranchii* | 118839 |
| Arthropoda | Malacostraca | Isopoda | Cirolanidae | *Natatolana vieta* | 256708 |
| Arthropoda | Malacostraca | Isopoda | Gnathiidae | *Gnathia mulieraria* | 257195 |
| Arthropoda | Malacostraca | Isopoda | Idoteidae | *Crabyzos longicaudatus* | 259908 |
| Arthropoda | Malacostraca | Isopoda | Idoteidae | *Euidotea bakeri* | 260323 |
| Arthropoda | Malacostraca | Isopoda | Idoteidae | *Synischia levidensis* | 263447 |
| Arthropoda | Malacostraca | Isopoda | Janiridae |  | 118258 |
| Arthropoda | Malacostraca | Isopoda | Philisciidae | *Haloniscus searlei* | 260492 |
| Arthropoda | Malacostraca | Isopoda | Phoratopodidae |  | 248305 |
| Arthropoda | Malacostraca | Isopoda | Plakarthriidae |  | 248307 |
| Arthropoda | Malacostraca | Isopoda | Serolidae | *Heteroserolis australiensis* | 260632 |
| Arthropoda | Malacostraca | Isopoda | Sphaeromatidae | *Amphoroidella elliptica* | 258839 |
| Arthropoda | Malacostraca | Isopoda | Sphaeromatidae | *Cassidinopsis lacertosa* | 258141 |
| Arthropoda | Malacostraca | Isopoda | Sphaeromatidae | *Cerceis tridentata* | 259708 |
| Arthropoda | Malacostraca | Isopoda | Sphaeromatidae | *Chitonopsis spatulifrons* | 259775 |
| Arthropoda | Malacostraca | Isopoda | Sphaeromatidae | *Exosphaeroma alii* | 257012 |
| Arthropoda | Malacostraca | Isopoda | Sphaeromatidae | *Exosphaeroma bicolor* | 257018 |
| Arthropoda | Malacostraca | Isopoda | Sphaeromatidae | *Platynympha longicaudata* | 262176 |
| Arthropoda | Malacostraca | Leptostraca | Nebaliidae |  | 147029 |
| Arthropoda | Malacostraca | Mysida | Mysidae |  | 119822 |
| Arthropoda | Malacostraca | Tanaidacea |  |  | 1133 |
| Arthropoda | Malacostraca | Tanaidacea | Apseudidae |  | 136153 |
| Arthropoda | Malacostraca | Tanaidacea | Apseudidae | *Carpoapseudes austroafricanus* | 247122 |
| Arthropoda | Malacostraca | Tanaidacea | Leptocheliidae |  | 136160 |
| Arthropoda | Malacostraca | Tanaidacea | Parapseudidae |  | 136156 |
| Arthropoda | Malacostraca | Tanaidacea | Paratanaidae | *Chondrochelia ignota* | 880869 |
| Brachiopoda | Rhynchonellata | Terebratulida | Terebratellidae | *Magellania flavescens* | 235686 |
| Chordata | Ascidiacea |  |  |  | 1839 |
| Chordata | Ascidiacea | Aplousobranchia | Polyclinidae | *Aplidium sp.* | 103474 |
| Chordata | Ascidiacea | Phlebobranchia | Ascidiidae | *Ascidia sp.* | 103483 |
| Cnidaria | Anthozoa |  |  |  | 1292 |
| Cnidaria | Anthozoa | Actiniaria | Actiniidae | *Anthopleura hermaphroditica* | 283353 |
| Echinodermata | Asteroidea | Forcipulatida | Asteriidae | *Uniophora granifera* | 292860 |
| Echinodermata | Asteroidea | Valvatida | Asterinidae | *Parvulastra exigua* | 459556 |
| Echinodermata | Echinoidea | Camarodonta | Temnopleuridae | *Amblypneustes ovum* | 513100 |
| Echinodermata | Echinoidea | Camarodonta | Temnopleuridae | *Amblypneustes pallidus* | 513102 |
| Echinodermata | Echinoidea | Camarodonta | Temnopleuridae | *Holopneustes purpurascens* | 569179 |
| Echinodermata | Echinoidea | Camarodonta | Temnopleuridae | *Temnopleurus michaelseni* | 513594 |
| Echinodermata | Holothuroidea |  |  |  | 123083 |
| Echinodermata | Holothuroidea | Holothuriida | Holothuriidae | *Holothuria (Panningothuria) austrinabassa* | 529055 |
| Echinodermata | Holothuroidea | Synallactida | Stichopodidae | *Australostichopus mollis* | 529443 |
| Echinodermata | Ophiuroidea |  |  |  | 123084 |
| Echinodermata | Ophiuroidea | Amphilepidida | Amphiuridae | *Amphipholis squamata* | 125064 |
| Echinodermata | Ophiuroidea | Amphilepidida | Ophiactidae | *Ophiactis tricolor* | 243472 |
| Echinodermata | Ophiuroidea | Amphilepidida | Ophionereididae | *Ophionereis sp.* | 123553 |
| Echinodermata | Ophiuroidea | Amphilepidida | Ophiotrichidae | *Ophiothrix caespitosa* | 244975 |
| Echinodermata | Ophiuroidea | Ophiacanthida | Ophiomyxidae | *Ophiomyxa australis* | 212437 |
| Mollusca | Bivalvia | Adapedonta | Hiatellidae | *Hiatella australis* | 545939 |
| Mollusca | Bivalvia | Arcida | Glycymerididae | *Glycymeris radians* | 213522 |
| Mollusca | Bivalvia | Arcida | Glycymerididae | *Tucetona sp.* | 204544 |
| Mollusca | Bivalvia | Cardiida | Cardiidae |  | 229 |
| Mollusca | Bivalvia | Cardiida | Cardiidae | *Acrosterigma cygnorum* | 381166 |
| Mollusca | Bivalvia | Cardiida | Cardiidae | *Nemocardium sp.* | 152920 |
| Mollusca | Bivalvia | Cardiida | Carditidae | *Cardita crassicosta* | 207679 |
| Mollusca | Bivalvia | Cardiida | Carditidae |  | 391090 |
| Mollusca | Bivalvia | Cardiida | Psammobiidae | *Hiatula alba* | 747137 |
| Mollusca | Bivalvia | Cardiida | Tellinidae | *Macomona deltoidalis* | 711134 |
| Mollusca | Bivalvia | Cardiida | Tellinidae | *Tellinides margaritinus* | 710898 |
| Mollusca | Bivalvia | Cardiida | Tellinidae | *Tellina sp.* | 138533 |
| Mollusca | Bivalvia | Carditida | Carditidae | *Centrocardita rosulenta* | 504887 |
| Mollusca | Bivalvia | Galeommatida | Lasaeidae | *Arthritica semen* | 754846 |
| Mollusca | Bivalvia | Galeommatida | Lasaeidae | *Mysella sp.* | 138186 |
| Mollusca | Bivalvia | Limida | Limidae | *Limaria orientalis* | 397092 |
| Mollusca | Bivalvia | Limida | Limidae | *Limatula strangei* | 505534 |
| Mollusca | Bivalvia | Lucinida | Lucinidae |  | 218 |
| Mollusca | Bivalvia | Myida | Teredinidae | *Nototeredo edax* | 397155 |
| Mollusca | Bivalvia | Mytilida | Mytilidae | *Brachidontes rostratus* | 505983 |
| Mollusca | Bivalvia | Mytilida | Mytilidae | *Brachidontes sp.* | 138215 |
| Mollusca | Bivalvia | Mytilida | Mytilidae | *Musculus nana* | 1030633 |
| Mollusca | Bivalvia | Mytilida | Mytilidae | *Xenostrobus inconstans* | 744581 |
| Mollusca | Bivalvia | Nuculanida | Nuculanidae | *Saccella crassa* | 866923 |
| Mollusca | Bivalvia | Ostreida | Pinnidae | *Pinna bicolor* | 207896 |
| Mollusca | Bivalvia | Ostreida | Pteriidae |  | 1775 |
| Mollusca | Bivalvia | Ostreida | Vulsellidae | *Electroma papilionacea* | 507101 |
| Mollusca | Bivalvia | Pectinida | Pectinidae | *Equichlamys bifrons* | 391844 |
| Mollusca | Bivalvia | Pectinida | Pectinidae | *Mimachlamys sp.* | 236718 |
| Mollusca | Bivalvia | Solemyida | Solemyidae | *Solemya australis* | 214549 |
| Mollusca | Bivalvia | Trigoniida | Trigoniidae | *Neotrigonia sp.* | 492172 |
| Mollusca | Bivalvia | Venerida | Chamidae | *Chama sp.* | 137775 |
| Mollusca | Bivalvia | Venerida | Mactridae |  | 230 |
| Mollusca | Bivalvia | Venerida | Mactridae | *Lutraria rhynchaena* | 216442 |
| Mollusca | Bivalvia | Venerida | Mactridae | *Mactrotoma antecedens* | 505726 |
| Mollusca | Bivalvia | Venerida | Mactridae | *Spisula trigonella* | 505773 |
| Mollusca | Bivalvia | Venerida | Mesodesmatidae | *Anapella cycladea* | 505791 |
| Mollusca | Bivalvia | Venerida | Mesodesmatidae | *Atactodea cuneata* | 505794 |
| Mollusca | Bivalvia | Venerida | Trapezidae |  | 23130 |
| Mollusca | Bivalvia | Venerida | Veneridae |  | 243 |
| Mollusca | Bivalvia | Venerida | Veneridae | *Bassina sp.* | 492471 |
| Mollusca | Bivalvia | Venerida | Veneridae | *Callista kingii* | 507403 |
| Mollusca | Bivalvia | Venerida | Veneridae | *Dosinia sp.* | 138636 |
| Mollusca | Bivalvia | Venerida | Veneridae | *Katelysia peronii* | 507722 |
| Mollusca | Bivalvia | Venerida | Veneridae | *Katelysia rhytiphora* | 826363 |
| Mollusca | Bivalvia | Venerida | Veneridae | *Katelysia scalarina* | 507723 |
| Mollusca | Bivalvia | Venerida | Veneridae | *Placamen flindersi* | 507875 |
| Mollusca | Bivalvia | Venerida | Veneridae | *Tawera lagopus* | 507919 |
| Mollusca | Bivalvia | Venerida | Veneridae | *Venerupis anomala* | 507981 |
| Mollusca | Bivalvia |  | Laternulidae | *Laternula sp.* | 138106 |
| Mollusca | Bivalvia |  | Myochamidae | *Myadora albida* | 506808 |
| Mollusca | Gastropoda | Aplysiida | Aplysiidae | *Aplysia sp.* | 137654 |
| Mollusca | Gastropoda | Caenogastropoda | Batillariidae | *Zeacumantus diemenensis* | 446441 |
| Mollusca | Gastropoda | Caenogastropoda | Batillariidae | *Zeacumantus plumbeus* | 853041 |
| Mollusca | Gastropoda | Caenogastropoda | Cerithiidae |  | 128 |
| Mollusca | Gastropoda | Caenogastropoda | Cerithiidae | *Cacozeliana granarium* | 473085 |
| Mollusca | Gastropoda | Caenogastropoda | Epitoniidae | *Epitonium tenerum* | 523995 |
| Mollusca | Gastropoda | Caenogastropoda | Turritellidae | *Gazameda iredalei* | 446529 |
| Mollusca | Gastropoda | Caenogastropoda | Turritellidae |  | 127 |
| Mollusca | Gastropoda | Cephalaspidea | Bullidae | *Bulla quoyii* | 510438 |
| Mollusca | Gastropoda | Cephalaspidea | Haminoeidae | *Liloa brevis* | 531577 |
| Mollusca | Gastropoda | Cephalaspidea | Philinidae | *Philine angasi* | 531549 |
| Mollusca | Gastropoda | Cycloneritida | Neritidae | *Nerita atramentosa* | 713884 |
| Mollusca | Gastropoda | Lepetellida | Fissurellidae |  | 111 |
| Mollusca | Gastropoda | Lepetellida | Scissurellidae | *Scissurella cyprina* | 493050 |
| Mollusca | Gastropoda | Littorinimorpha | Hipponicidae | *Sabia australis* | 598647 |
| Mollusca | Gastropoda | Littorinimorpha | Hydrobiidae |  | 120 |
| Mollusca | Gastropoda | Littorinimorpha | Littotinidae | *Bembicium nanum* | 445492 |
| Mollusca | Gastropoda | Littorinimorpha | Littotinidae | *Bembicium vittatum* | 445480 |
| Mollusca | Gastropoda | Littorinimorpha | Naticidae | *Conuber conicum* | 585289 |
| Mollusca | Gastropoda | Littorinimorpha | Naticidae | *Natica sp.* | 138240 |
| Mollusca | Gastropoda | Littorinimorpha | Naticidae | *Tanea sagittata* | 570158 |
| Mollusca | Gastropoda | Littorinimorpha | Rissoinidae | *Rissoina crassa* | 598001 |
| Mollusca | Gastropoda | Littorinimorpha | Rissoinidae | *Rissoina fasciata* | 765955 |
| Mollusca | Gastropoda | Littorinimorpha | Vitrinellidae |  | 153704 |
| Mollusca | Gastropoda | Lottioidea | Eoacmaeidae | *Eoacmaea calamus* | 458652 |
| Mollusca | Gastropoda | Neogastropoda | Buccinoidea | *Cominella lineolata* | 490925 |
| Mollusca | Gastropoda | Neogastropoda | Columbellidae | *Mitrella australis* | 511477 |
| Mollusca | Gastropoda | Neogastropoda | Columbellidae | *Mitrella lincolnensis* | 511520 |
| Mollusca | Gastropoda | Neogastropoda | Fasciolariidae | *Propefusus australis* | 1319812 |
| Mollusca | Gastropoda | Neogastropoda | Murcidae | *Bedeva paivae* | 181032 |
| Mollusca | Gastropoda | Neogastropoda | Murcidae | *Bedeva vinosa* | 869741 |
| Mollusca | Gastropoda | Neogastropoda | Muricidae | *Pterochelus triformis* | 406234 |
| Mollusca | Gastropoda | Neogastropoda | Nassariidae |  | 151 |
| Mollusca | Gastropoda | Neogastropoda | Nassariidae | *Nassarius pauperatus* | 572153 |
| Mollusca | Gastropoda | Neogastropoda | Nassariidae | *Nassarius pyrrhus* | 572154 |
| Mollusca | Gastropoda | Neogastropoda | Terebridae | *Duplicaria kieneri* | 438575 |
| Mollusca | Gastropoda | Neogastropoda | Volutomitridae | *Peculator porphyria* | 450663 |
| Mollusca | Gastropoda | Pylopulmonata | Amphibolidae | *Salinator fragilis* | 549356 |
| Mollusca | Gastropoda | Seguenziida | Chilodontidae | *Granata sp.* | 512104 |
| Mollusca | Gastropoda | Trochida | Trochidae | *Austrocochlea constricta* | 546938 |
| Mollusca | Gastropoda | Trochida | Trochidae | *Clanculus dunkeri* | 594193 |
| Mollusca | Gastropoda | Trochida | Trochidae | *Clanculus limbatus* | 594200 |
| Mollusca | Gastropoda | Trochida | Trochidae | *Clanculus philippi* | 594206 |
| Mollusca | Gastropoda | Trochida | Trochidae | *Clanculus plebejus* | 594207 |
| Mollusca | Gastropoda | Trochida | Trochidae | *Phasianotrochus eximius* | 573223 |
| Mollusca | Gastropoda |  | Lottiidae |  | 7173 |
| Mollusca | Gastropoda |  | Lottiidae | *Notoacmea flammea* | 456623 |
| Mollusca | Gastropoda |  | Nacellidae | *Cellana tramoserica* | 325467 |
| Mollusca | Gastropoda |  | Patellidae |  | 113 |
| Mollusca | Polyplacophora |  |  |  | 55 |
| Mollusca | Polyplacophora | Chitonida | Acanthochitonidae | *Acanthochitona sp.* | 137613 |
| Mollusca | Polyplacophora | Chitonida | Acanthochitonidae | *Acanthochitona sueurii* | 386519 |
| Mollusca | Polyplacophora | Chitonida | Chitonidae | *Rhyssoplax sp.* | 385600 |
| Mollusca | Polyplacophora | Chitonida | Ischnochitonidae | *Ischnochiton sp.* | 138088 |
| Mollusca | Polyplacophora | Chitonida | Ischnochitonidae | *Ischnochiton variegatus* | 848106 |
| Mollusca | Polyplacophora | Chitonida | Ischnochitonidae | *Stenochiton cymodocealis* | 386066 |
| Mollusca | Polyplacophora | Chitonida | Ischnochitonidae | *Stenochiton pilsbryanus* | 386069 |
| Mollusca | Polyplacophora | Chitonida | Ischnochitonidae | *Stenochiton sp.* | 385550 |
| Mollusca | Polyplacophora | Chitonida | Mopaliidae |  | 23074 |
| Mollusca | Scaphopoda | Dentallida | Dentaliidae | *Paradentalium intercalatum* | 344529 |
| Mollusca | Scaphopoda | Gadilia | Gadilidae | *Cadulus vincentianus* | 344307 |
| Nemertea |  |  |  |  | 152391 |
| Nemertea | Pilidophora | Heteronemertea | Lineidae | *Notospermus sp.* | 122375 |
| Porifera | Calcarea | Leucosolenida | Leucosoleniidae |  | 131616 |
| Sipuncula |  |  |  |  | 1268 |
| Sipuncula | Sipunculidea | Golfingiida | Golfingiidae | *Golfingia* sp. | 136021 |


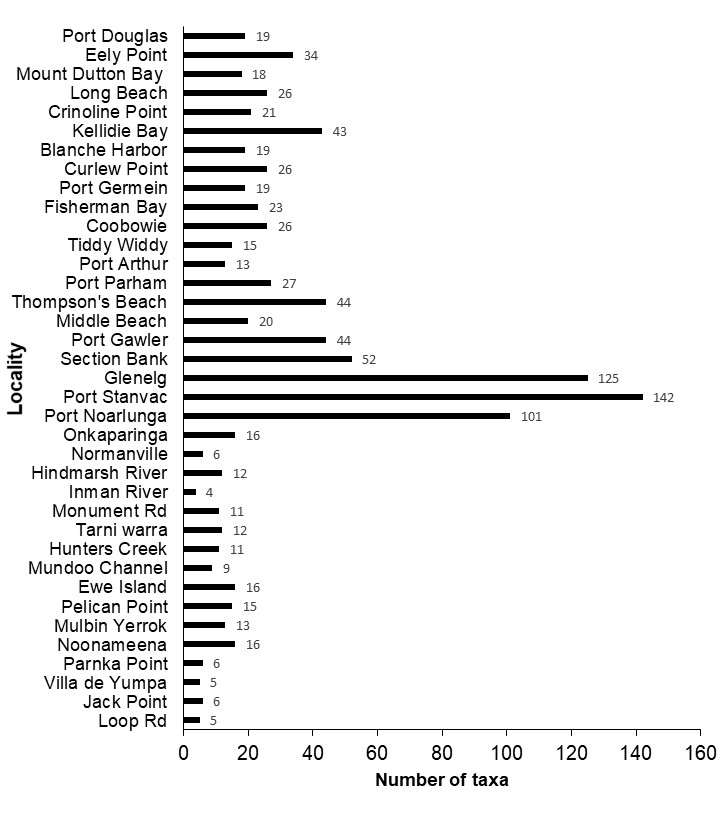


**Figure S1.** Number of taxa recorded across the 37 localities of South Australia from 2008 to 2020.

**
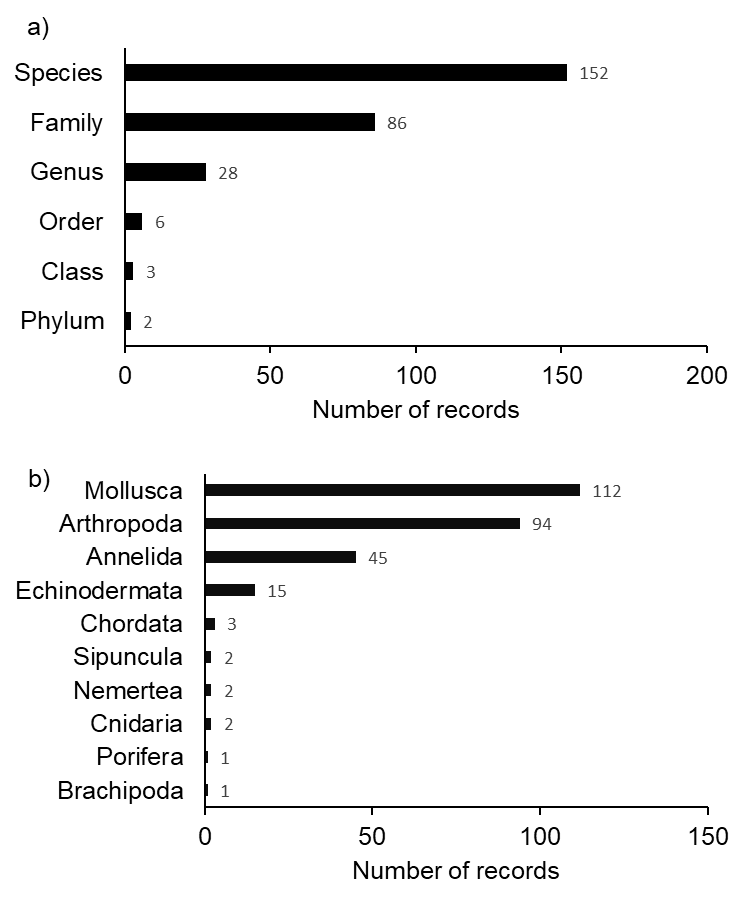
**

**Figure S2.** a) Number of taxa assessed at different levels of identification from all locations and sites from 2008 to 2020. b) Number of taxa recorded by Phylum across all locations and sites from 2008 to 2020.
