# Supplementary material for: Establishing the South Australian Macrobenthic Traits (SAMT) database: A trait classification for functional assessments
Source: Ecol Evol. 2020 Nov 27;10(24):14372–87. doi: 10.1002/ece3.7040 (PMC7771161; doi:10.1002/ece3.7040)
Supplement: Supplementary file 2 — Supplementary Material [file ECE3-10-14372-s001.pdf]

Appendix 1: Using SAMT package in R

Orlando Lam-Gordillo, Ryan Baring, Sabine Dittmann

2020-08-04

This document illustrates the features and functionality of the **SAMT** R package. **SAMT** R package was developed as an easy method to manipulate and analyse taxa and trait data of macrobenthic fauna for people with and without experience in using R software. Detailed examples on the usage of the package are provided in order to show its functionality.

Traits

Traits are all the features and properties of an organism that can be measured. Common examples of traits are life history, morphology, physiology and behaviour characteristics that organisms can exhibit. For example, the trait feeding mode that enclose different trait-modalities: deposit feeder, filter/suspension, grazer/scrapper, omnivore, predator, scavenger/opportunist, and sub-surface deposit feeder.

Trait information is always difficult to find, the collection of trait information is often considered a time-consuming task, mainly due to gaps in the knowledge of traits, deficiency on species trait information, and data accessibility. SAMT database was created to overcome these issues and to facilitate further research on functional perspectives in marine waters of southern Australia.

SAMT database

The South Australian Macrobenthic Traits (SAMT) database contains trait information of 277 macrobenthic taxa and covers 13 traits and 54 trait-modalities.

SAMT package in R

SAMT is an R package that collects functions based on/and for the analysis of the SAMT database.

How to install

There are two options for installing the package:

- Installing from GitHub

First, install or call devtools

```
install.packages("devtools")
library(devtools)
```

and then install SAMT

```
devtools::install_github("OrlandoLam/SAMT")
```

- Installing from local file (R package)

Download the SAMT_1.0.0.tar.gz package and install using R or RStudio.

Usage of SAMT

Trait information is often required when addressing functional approaches of an ecosystem, however, trait information is scarce or dispersed across multiple platforms, websites, collections, or databases. The **SAMT** package contains the trait information from 277 macrobenthic taxa of South Australia, making easy to assess functional perspectives.

The **SAMT** package contains five functions:

Three of the functions are for showing the information contained in SAMT database:

`Taxa.list`: Display a data frame with the list of taxa provided in SAMT.

`Traits.list`: Shows a data frame with the list of traits used in SAMT.

`SAMTdb`: The database designed for marine Macroinvertebrate taxa in South Australia.

And the other two for calculating trait values when adding information of sites, replicates, or/and taxa abundance:

`TVT`: Calculates the Traits Values by Taxa.

`TVSR`: Calculates the Traits Values per Site/Replicate.

Example

First load the **SAMT** package.

```
library(SAMT)
```

Then, load some data

`data(dummy_data)` *(Dataset that contains taxa abundance values by sites)*

`data(SAMTdatabase)` *(Dataset that contains trait values by taxa)*

To display the taxa list use `Taxa.list ()` , with this function you can check all taxa names that **SAMT** provide, and decide if using all or doing a selection of particular taxa for your further analyses.

To display the list of traits used in **SAMT** use `Traits.list ()` , and a data frame showing the trait list will appear. Alternatively, to show the trait-modalities use the argument `Traits` and a data frame with the list of traits and trait-modalities will be shown.

For displaying the SAMT database the function `SAMTdb()` needs to be used. A data frame with taxa and traits information will appear. This provides an opportunity to select using all traits or select some of particular interest. Alternatively, another way to display this information is using the argument `SAMTdatabse` but this argument will not provide the dataset as data frame.

The first main function of the **SAMT** package is `TVT(a,b)`, which calculates the trait value at taxa level taking into consideration the taxa abundance (Functional classification by Taxa abundance). Where **a** is a database or data matrix that contains taxa abundance per site/replicate. In all cases, taxa labels are required to match with the traits database. And **b** is `SAMTdatabase`, but it could be another database or data matrix that contains the same number of functional traits by taxa that are within SAMTdatabase. In all cases, taxa labels are required to match with the abundance database.

```
TVT(dummy_data, SAMTdatabase)
```

Results from the function `TVT(a,b)` can be stored as an object:

```
R1<-TVT(dummy_data, SAMTdatabase)
```

for further analyses within R, or exported as a .csv file:

```
write.csv(R1, file = "name.csv")
```

The second main function of **SAMT** package is `TVSR(a,b)`, which calculates the trait value by site/replicate level (Functional classification * Taxa Abundance (specific location/time)). Where **a** is a database or data matrix that contains taxa abundance per site/replicate. In all cases, taxa labels are required to match with the traits database. And **b** is `SAMTdatabase`, but it could be another database or data matrix that contains the same number of functional traits by taxa that are within SAMTdatabase. In all cases, taxa labels are required to match with the abundance database.

```
TVSR(dummy_data, SAMTdatabase)
```

Results from the function `TVSR(a,b)` can be stored as an object:

```
R2<-TVSR(dummy_data, SAMTdatabase)
```

for further analyses within R, or exported as a .csv file:

```
write.csv(R2, file = "name.csv")
```

*Note: `TVT()` and `TVSR()` are sensible to the length to the data frame or data matrix to use. If not using SAMTdatabse, please check before the length of the database using `dim()`, **b** data frame should have 54 columns, and the first column should contain the taxa names.*
